# Exploring equity in health and poverty impacts of control measures for SARS-CoV-2 in six countries

**DOI:** 10.1136/bmjgh-2021-005521

**Published:** 2021-05-01

**Authors:** Sedona Sweeney, Theo Prudencio Juhani Capeding, Rosalind Eggo, Maryam Huda, Mark Jit, Don Mudzengi, Nichola R Naylor, Simon Procter, Matthew Quaife, Lela Serebryakova, Sergio Torres-Rueda, Veronica Vargas, CHiL COVID Working Group, Fiammetta Bozzani, Nicholas G Davies, Henning Jensen, Marcus Keogh-Brown, Mishal Khan, Nichola Kitson, Nuru Saadi, Julia Shen, Anna Vassall

**Affiliations:** London School of Hygiene & Tropical Medicine, London, UK; 1 Department of Global Health and Development, London School of Hygiene & Tropical Medicine, London, UK; 2 University of the Philippines Manila, Manila, The Philippines; 3 Department of Infectious Disease Epidemiology, London School of Hygiene & Tropical Medicine, London, UK; 4 The Aga Khan University, Karachi, Pakistan; 5 University of Hong Kong School of Public Health, Hong Kong, China; 6 The Aurum Institute for Health Research, Johannesburg, South Africa; 7 National Center for Disease Control and Public Health, Tbilisi, Georgia; 8 Facultad de Economía y Negocios, Universidad Alberto Hurtado, Santiago, Chile

## Abstract

**Background:**

Policy makers need to be rapidly informed about the potential equity consequences of different COVID-19 strategies, alongside their broader health and economic impacts. While there are complex models to inform both potential health and macro-economic impact, there are few tools available to rapidly assess potential equity impacts of interventions.

**Methods:**

We created an economic model to simulate the impact of lockdown measures in Pakistan, Georgia, Chile, UK, the Philippines and South Africa. We consider impact of lockdown in terms of ability to socially distance, and income loss during lockdown, and tested the impact of assumptions on social protection coverage in a scenario analysis.

**Results:**

In all examined countries, socioeconomic status (SES) quintiles 1–3 were disproportionately more likely to experience income loss (70% of people) and inability to socially distance (68% of people) than higher SES quintiles. Improving social protection increased the percentage of the workforce able to socially distance from 48% (33%–60%) to 66% (44%–71%). We estimate the cost of this social protection would be equivalent to an average of 0.6% gross domestic product (0.1% Pakistan–1.1% Chile).

**Conclusions:**

We illustrate the potential for using publicly available data to rapidly assess the equity implications of social protection and non-pharmaceutical intervention policy. Social protection is likely to mitigate inequitable health and economic impacts of lockdown. Although social protection is usually targeted to the poorest, middle quintiles will likely also need support as they are most likely to suffer income losses and are disproportionately more exposed.

## Introduction

In the early stages of the COVID-19 pandemic, many countries around the world adopted stringent non-pharmaceutical interventions (NPIs) to slow the spread of SARS-CoV-2 and avoid exceeding hospital bed capacity.^1^ These interventions included a variety of containment and closure policies including school closures, curfews and other restrictions in movement, business closures and requirements to work from home, and are sometimes broadly termed termed ‘lockdown’.^2, 3–6^ These policies were designed to mitigate the spread of infection with SARS-CoV-2 and reduce the burden of COVID-19 on the health system, and were particularly effective in areas where they were implemented before widespread transmission.^7,8^ However, the magnitude of the social and economic impacts of lockdown policies has given rise to heated debates surrounding the net benefit of further restrictions.^9^ As the pandemic continues and until vaccines can be widely implemented, there is an ongoing need for difficult policy decisions on further NPIs.

Lessons from other pandemics inform us that the economic impacts of pandemics can be pernicious and long-lasting, and that equity must be a key consideration in considering policy response.^10–13^ The COVID-19 pandemic has brought the socioeconomic determinants, and consequences, of health into sharp relief.^14^ Disadvantaged populations have higher COVID-19 infection rates and higher death rates than their more privileged counterparts, so unmitigated epidemics could hit these populations hardest^15, 16^ Overcrowded housing, heightened stress, chronic comorbidities and inability to socially distance are major drivers of COVID-19 infection and disease severity, and often unavoid-able for those without adequate resources.^17^ It is also clear that the pandemic has exacerbated existing structural inequities where adequate economic support has not been available to lessen the blow of income loss for disadvantaged groups.^18^

In most settings where lockdown policies have been introduced, social protection interventions are integral to their implementation. As many countries are facing COVID-19-related declines in tax revenues and increasing demands on public spending, countries increasingly need better information to inform social protection policies in their response to the pandemic. This includes tools to help them rapidly assess where and how limited funds for social protection can be best mobilised, so that equity can be simultaneously considered alongside aggregate health and economic impact in the response to COVID-19. While there are complex models to inform both potential health and macro-economic impact of policy responses to COVID-19, there are few tools available to rapidly assess potential equity impacts and social protection needs.^19^ Some existing models have helped decision makers in the USA^20^ and Uruguay^21^ to understand equity and poverty trade-offs of policies. However, these models are reliant on household-level census data, which is often absent in low-income and middle-income countries.

The aim of this work is to explore the potential to use globally available data in a policy-focused model to understand the equity impact of lockdowns in various settings, in terms of risk of exposure to COVID-19 and income loss. We demonstrate a pragmatic approach to exploring inequity of COVID-19 policies alongside health impact and macro-economic modelling, comparing these risks across six countries in different income groups. We focus on inequities in socioeconomic status (SES) due to limited data availability, although there are also many other aspects of concern when considering equity—including structural discriminations pertaining to race, gender, place of residence and other forms of deprivation.

## Methods

### Model structure and data sources

We created an economic model to illustrate and simulate the equity consequences of the initial stringent lockdowns imposed to curb the spread of COVID-19 in six countries, including two lower-middle-income countries (the Philippines, Pakistan), two upper-middle-income countries (South Africa, Georgia) and two high-income countries (United Kingdom (UK), Chile).^22^ We chose these countries because they span a range of country income groups and workforce structures, and all countries introduced lockdown measures in March and April 2020.^1^ Our assumptions defining ‘full lockdown’ in each country are detailed in online supplemental file 2; in each country, lockdowns involved restriction of nonessential movement, closure of non-essential businesses and schools and encouragement of teleworking where possible. In some countries, lockdowns also included introduction of curfews, but this was not included in our model.

**Figure 1:**
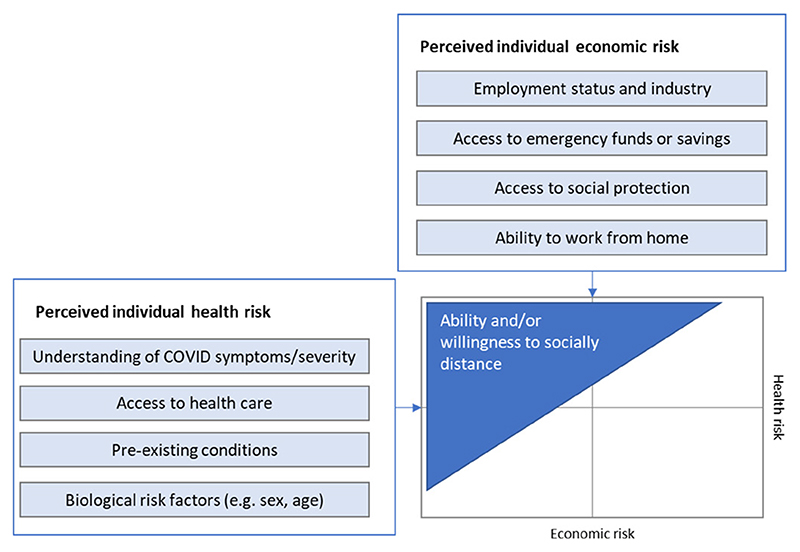
Model structure and assumptions.

Our conceptual basis for the model is illustrated in figure 1, adapted from Vassall *et al*.^23^ We consider ability and willingness to socially distance as factors of individual perception of economic and health risk associated with COVID-19. Where individual perception of health risks from COVID-19 are high and economic risk is low, people will be highly willing to self-isolate. In contrast, where the economic risks associated with self-isolating are high compared with perceived health risks (eg, because isolating will result in food insecurity or other long-term effects), people will likely be less willing to self-isolate.

Where public policies enforce physical distancing through NPIs, these trade-offs are also driven by individual employment status and the degree to which industries are impacted by NPIs. The economic risk of staying home is very low for those who can work remotely. If an individual is an essential worker, their ability to socially distance will be far lower without higher-stakes health/economic trade-offs, such as leaving their job. Where industries are closed due to lockdown, those losing income may be forced to seek other means of survival if there is no social safety net available.

We consider the impact of lockdown on two dimensions of individual economic risk. First, we estimate risk of economic hardship due to losing income during the lockdown. Next, we estimate the number of people unable to socially distance due to economic concerns. We do not consider individual perceptions of health risk or willingness to socially distance for reasons other than economic concerns, nor do we evaluate variations in access to sanitation measures to protect against COVID-19 infection. Our analysis is restricted to the first period of full lockdown in each country before any scaling back of stringency in each country and focuses only on people within the labour force (including employed and unemployed persons).

We first quantified the number of workers in each 2-digit International Standard Classification of Occupations (ISCO) occupation code (ISCO-08) by country.^24^ We used datasets on the total number of employees by occupation, and the total number of unemployed people by former occupation from the International Labour Organization (ILO).^25, 26^ These data include all people of working age in paid employment or self-employment, including informal employment and employment in the informal sector. We do not consider people outside the labour force (including students, retirees, permanently disabled individuals, homemakers and discouraged jobseekers) as there is insufficient data on sources of income for these groups. Where the most recent data used older occupation codes (ISCO-88), these were reclassified into ISCO-08 codes using crosswalks provided by the ILO.^27^ We used information on the distribution of each occupation across 20 codes from the Statistical Classification of Economic Activities in the European Community representing economic activities, or industries, to crosstabulate the total number of people in each occupation by industry.^28^ We used the most recent information available for each country, which was 2017 in Chile, 2018 in Pakistan and 2019 in South Africa, the Philippines, Georgia and the UK. We did not adjust estimates from earlier years, assuming the broad structure of employment in the population would not change substantially between 2017 and 2020.

### ‘Essential’ jobs and teleworkable jobs

For each country, we used news searches, policy documents and previous research to identify the percentage of workers in each industry that would be identified as ‘essential’ and allowed to work in-person as normal during lockdown.^2–5^ We assumed all ‘essential’ workers would continue working in-person and therefore would continue earning as normal but would be exposed to possible infection with SARS-CoV-2 during lockdown. The number of people in ‘essential’ industries varied by country, for example, in South Africa some workers in the mining sector were considered essential as public revenues depend significantly on this sector. In Chile, governmental designations of ‘essential’ industries were not enforceable, leading to many more companies continuing in-person operation.^29^

We next identified the percentage of workers in activities that were ‘subject to teleworking’, meaning people would be able to continue working if their job were tele-workable, and otherwise would not be able to continue work. We evaluated the working status of those in ‘subject to teleworking’ activities using a ‘teleworkability index’ constructed using data from two Occupational Network (O*NET) surveys conducted in the USA.^30^ The teleworkability index was based on survey responses covering work context and work activities, for example, occupations requiring specialised tools or involving the operation of vehicles or equipment were assumed not to be tele-workable. This index has been extrapolated and applied to a range of countries, assuming that the same factors would influence teleworkability in all countries.^30–32^ We compared the estimated number of ‘teleworkable’ jobs against the total number of internet users in each country to verify estimates.

### ‘Closed’ jobs, non-teleworkable jobs and unemployed people

We finally estimated the percentage of workers by occupation in activities that were ‘closed’, or restricted from continuing economic activity during lockdown, such as the hospitality industry. We assumed people in ‘closed’ activities would not continue working during lockdown, even if their job were ‘teleworkable’, as activity in the industry would be largely halted during lockdown.

We estimated the total number of people out of work during lockdown as the sum of unemployed people, people in ‘closed’ activities and people who were in a job that was not ‘essential’ and not teleworkable. We assumed that a proportion of these people would have access to social protection which would replace their lost income and allow them to stay socially distanced during lockdown. The coverage of social protection was estimated at 20% in the base case,^33^ and we assumed social protection was not targeted at any particular population or SES quintile. We further assumed that a portion of people losing income would have access to private emergency funds (eg, savings, loans or gifts from family/friends) to tide them over financially until lockdown ended in the absence of a social security umbrella or furlough scheme. We estimated access to emergency funds using data from the Global Findex Database.^34^ This global survey includes information on the percentage of survey respondents reporting that in case of an emergency it would be possible for them to come up with 1/20 of gross national income (GNI) per capita in local currency within the next month. We input into our model the percentage of those in the labour force reporting access to emergency funds by SES quintile, for each country. We assumed those with no access to emergency funds and not receiving any social support would need to continue some economic activity to survive, even if this were illegal or unsafe, and therefore would not be able to socially distance during lockdown.

### Outcomes by SES quintile

To summarise, using the above-listed assumptions and estimations, we identified two sets of non-mutually exclusive binary outcomes:

1. Income maintained versus income lost. ‘Essential’ workers and people who were able to telework continued earning income as normal; people in ‘closed’ activities and people unable to telework faced a loss of income if they were unable to access social protection.
2. Able to socially distance versus not able to socially distance. People who were able to telework, and those out of work with access to emergency funds or social protection would be able to socially distance during lockdown. ‘Essential’ workers would not be able to socially distance during lockdown, and those with no access to emergency funds or social protection would find work even if this were unsafe or illegal. We did not consider any varying degrees of protection.

To evaluate socioeconomic inequalities in the abovelisted outcomes, we generated country-specific SES quintiles. For each country, we ranked the labour force using International Socio-Economic Index (ISEI) scores as an indicator of SES by occupation,^35, 36^ and then split the labour force into five equal quintiles. ISEI scores are identified by ISCO-08 occupation group, and range from 10 (subsistence farmers) to 70 (science and engineering professionals). The process for development of the ISEI scores is described further by Ganzeboom *et al*.^35, 36^ Where ISEI scores were tied between multiple occupations, we used the Standard International Occupational Prestige Scale^37^ to break the tie and help split the population into equal groups.

Estimates for each of the four outcomes listed above are presented by SES quintile for each country. We estimated a concentration index, a measure of inequality that indicates the extent to which an outcome is concentrated among the advantaged or disadvantaged. The concentration index represents twice the area between a line of equality (a 45-degree line) and the curve representing the cumulative proportion of people experiencing the given outcome, when the population is ranked by SES (the concentration curve).^38^ It is bounded between –1 and 1, where a value less than zero indicates that the outcome is concentrated in lower quintiles, and a concentration index above zero indicates that the outcome is concentrated in higher quintiles. It is estimated as: $C=\frac{2}{\mu }\text{cov}(o,r)$ where *o* is the outcome variable (ie, income loss or inability to socially distance during lockdown), *μ* is its mean and *r* is the fractional rank in the distribution of SES.^39, 40^ We used the Stata module ‘concindc’ to calculate the concentration index and its SE for each outcome.^41^

### Scenario analysis: social protection

Finally, we conducted a scenario analysis to evaluate the potential for social protection to mitigate the impact of income loss and improve ability to socially distance. We estimated the impact of providing social protection to 20%, 50% and 80% of people losing income for each day the country spent in the most stringent tier during the first lockdowns.^1^ For each level of social protection coverage, we also estimated the average cost per person covered per day as equivalent to 1/365th of the 2020 GNI per capita for each person losing income due to lock-down.^42^

### Patient and public involvement

This analysis used only publicly available data from global data sources, in an effort to inform rapid modelling where primary data are lacking. As no data were collected for the purposes of this study, it was not relevant to involve patients or the public in the design, conduct, reporting, or dissemination plans of our research.

## Results

**Figure 2:**
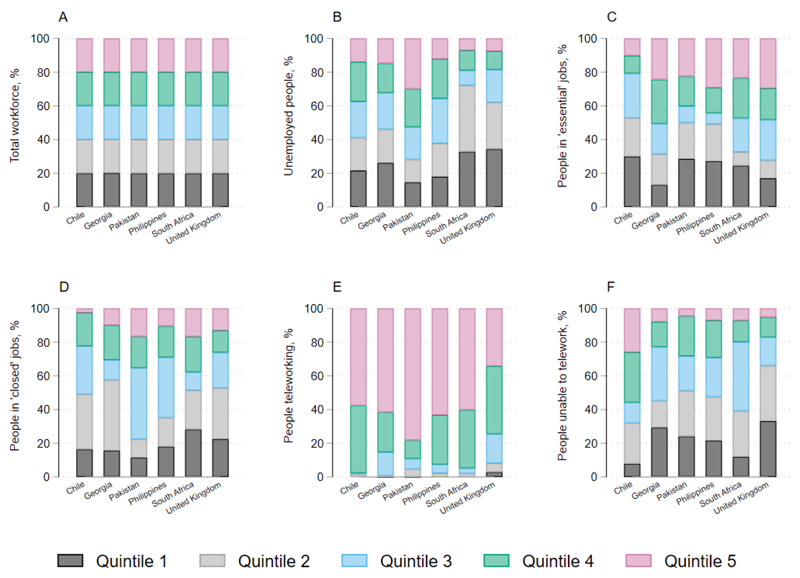
Economic impact of lockdown measures by socioeconomic status quintile.

The makeup of the workforce varied in each country, however SES quintiles contained equal segments of the population by design (figure 2, panel A). The full details of employment by occupation for each country are provided in online supplemental table 2.

### ‘Essential’ and teleworkable jobs

The proportion of the labour force that was able to telework during lockdown varied from 10% in the Philippines to 21% in the UK (online supplemental table 1). The UK has a higher number of managers and professionals, and therefore a larger proportion of the workforce can work from home. In contrast, Pakistan, the Philippines and South Africa have a larger number of workers in mining, construction, manufacturing and other elementary occupations, most of which are not conducive to working from home. People who were able to telework were largely concentrated in SES quintiles 4 and 5 (figure 2, panel E).

The overall proportion of the labour force in ‘essential’ activities, who would continue earning as normal during lockdown but would not be able to socially distance, varied from 25% in Pakistan and South Africa to 52% in Chile (online supplemental table 3). In Chile, Pakistan and the Philippines, a large portion of ‘essential’ workers were in elementary occupations, while this was mostly health professionals and health associate professionals in Georgia and the UK. In Chile, Pakistan and the Philippines, essential workers were more concentrated in lower SES quintiles, while in the UK and Georgia essential workers were more concentrated in higher SES quintiles (figure 2, panel C).

### ‘Closed’ and non-teleworkable jobs, and unemployed people

Overall, the proportion of the labour force that was unemployed, working in activities that were closed during lockdown or working in activities that were not teleworkable ranged from 31% in Chile to 64% in Pakistan (; figure 2, panels B, D, F). In all countries except South Africa, personal service workers and sales workers had the largest percentage of income losses; in South Africa, this was clerical support workers and cleaners and helpers online supplemental table 4.

Of those losing income due to lockdown, an average of 39% (ranging from 26% in South Africa to 62% in the UK) would have access to emergency funds in the event of sudden income loss. The model assumes in the base case that a further 20% of people with income loss would receive some form of social assistance, while the remainder would need to resume some form of economic activity. In our base case estimates, an average of 52% of the labour force would be unable to socially distance during lockdown due to economic concerns (ranging from 40% in the UK to 67% in Chile).

### Outcomes by quintile

**Figure 3:**
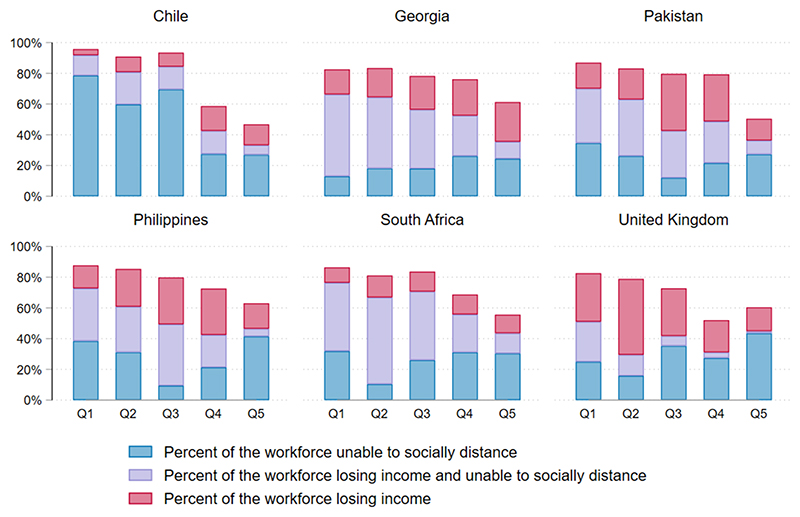
Income loss and ability to socially distance during lockdown by country.

figure 3 summarises the number of people by quintile encountering our two key outcomes: loss of income and inability to socially distance during lockdown. Chile had the smallest percentage of people losing income during lockdown (25%), but the highest proportion of people unable to socially distance during lockdown (67%); this was a result of relatively more companies continuing in-person operation as normal as governmental designations of ‘closed’ industries were non-enforceable. The UK had the lowest percentage of people unable to socially distance during lockdown (40% of the labour force) because more occupations were ‘teleworkable’. In all four middle-income countries, high numbers of people experienced both income loss and inability to socially distance due to a lack of access to emergency funds. This was especially high in the lower income quintiles. For most countries, only the highest SES quintile had a high number of people who were both earning as normal and protected from exposure to COVID-19 during lockdown.

**Figure 4:**
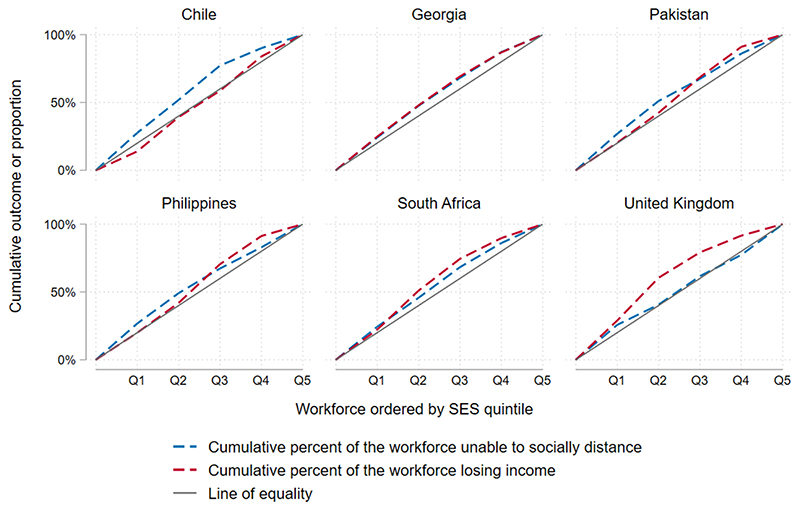
Concentration curves—socioeconomic inequalities in economic and health risk.

The potential impact of lockdown was regressive in all countries (figure 4; table 1). Disproportionately more people in lower SES quintiles were unable to socially distance during lockdown in all countries, and more experienced income loss during lockdown in all countries except Chile. Concentration indices for income loss were lowest in the UK (−0.24), while concentration indices for inability to socially distance were lowest in Chile (−0.19) and Pakistan (−0.13).

### Scenario analysis: social protection

**Figure 5:**
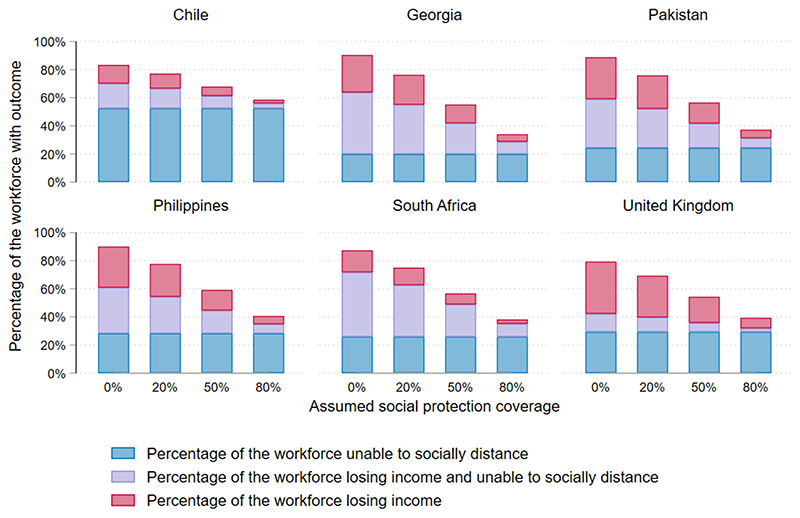
Scenario analysis.

Figure 5 shows the results of our scenario analysis looking at the impact of changes in social protection. Our analysis shows that social protection is very important for mitigating both health and economic impacts of lockdown. Increasing the assumed availability of social protection from 20% to 80% reduced the overall percentage of people losing income from an average of 46% (25%–56%) to 11% (6%–14%). It also increased the overall percentage of people able to socially distance from 48% (33%–60%) to 66% (44%–71%) (table 1).

Untargeted social protection had no impact on the concentration indices for income loss, although it reduced overall income loss across the population in the workforce. For all countries except Chile improved social protection reduced inequity in ability to socially distance. This was because lower SES quintiles had less access to emergency funds, and therefore more people in lower SES quintiles relied on social protection to enable them to socially distance when losing income. In Chile, relatively large numbers of people continued in-person work as normal, so the group of people losing income in the base case was small.

Finally, our scenario analysis finds that the cost of achieving 20% social protection coverage would be equivalent to an average of 0.6% gross domestic product (GDP) in the base case (ranging from 0.1% in Pakistan to 1.1% in Chile). On average across countries, increasing social protection from 20% to 80% coverage during the most stringent lockdown period would require increased government spending equivalent to 1.8% of 2020 GDP (moving from 0.6% to 2.4% of GDP on average).

## Discussion

Our analysis provides an example of a rapid, illustrative approach using global datasets to inform implementation of NPIs and corresponding social protection interventions, across settings. A static economic model such as the one presented cannot reflect the dynamic reality or complexity of changing economies during the COVID-19 pandemic. However, it can highlight areas of concern where further investigation and policy consideration is needed, in the absence of being to stratify more complex epidemiological and economic outcomes by socioeconomic group.

We find that the stringent lockdowns seen in the early response to the pandemic are likely to have had a significant impact on equity in all settings. The overall numbers of people losing income and/or unable to socially distance in the absence of social protection were high in all countries. Lower SES quintiles were consistently put at both greater health and/or economic risk during lockdown. Even where more equitable distributionally, the absolute impact of widespread loss of income may be catastrophic, especially in countries where incomes are lower to start with.

Overall, our estimated cost for social protection interventions to mitigate the impact of lockdown was relatively low in all settings when compared with the costs of an unmitigated COVID-19 epidemic. Providing the equivalent GNI per capita per day for each person losing income would require an average of 2% of GDP. In comparison, OECD countries usually spend an average of 19.8% GDP on social protection every year (ranging from 7% in Mexico to 31% in France).^43^ Paying people to stay home may therefore be a reasonable policy option when compared with the estimated costs of treating an unmitigated COVID-19 epidemic, which has been estimated as 8.72%–216.36% of current health spending globally, or 0.86%–10.88% GDP,^44^ excluding the intrinsic value of avoided morbidity and mortality.

We find that improved access to social protection can reduce both economic and health impact of stringent lockdowns. Our estimates of the cost of replacing lost income are approximately in line with what is known about the costs of the furlough scheme in the UK.^45^ However, without good data on the actual breadth and reach of social protection interventions in all settings, it is difficult to draw conclusions on the actual impact that social protection schemes had on outcomes in each country. While we estimated 20% access to social protection in the base case, changes in this assumption had substantial resulting changes in our outcome estimates. The ability of the model to inform implementation and targeting of social protection could be improved if better data on the accessibility and uptake of these schemes were available.

The affordability of social protection measures may, however, also change over time. The model uses simple assumptions intending to be illustrative and does not consider the complex timelines of varying stringency in movement restrictions and other NPIs over time. Our estimates also reflected only the first full period of lockdown in each country. Depending on lockdown duration and access to social protection, the impact and costs of social protection schemes might be higher. For example, in settings such as Chile, where the period of partial lockdown was relatively long, the costs of covering reduced incomes for this entire period may be much higher. Further analysis is needed to understand the current coverage of social protection schemes, and the impact of length of lockdown on social protection effectiveness and cost.

Our analysis also assumes that social protection interventions are untargeted, with equal coverage across all SES quintiles. Due to a lack of appropriate data, our model could not account for the complexity of social protection responses to COVID-19 in the analysed countries, which included a wide range of policies such as targeted and untargeted transfer schemes, improvement of employment protection, increased access to unemployment benefits or health insurance schemes and changes to banking regulations.^46^ Improved systematic data on eligibility and access to social protection schemes could improve analyses like this in future.

Targeted social protection may further improve equity and reduce the costs of covering lost income. Countries considering targeting of social protection in response to COVID-19 should consider carefully where populations with different kinds of risk need different types of interventions to manage the economic and health impacts of lockdowns. Our analysis shows that people in SES quintiles 2–3 experience disproportionate income loss and will likely need income support. In some countries, SES quintile 1 had more essential workers, and therefore fewer people losing income than quintiles 2 and 3. These people may have less need for replacement of income loss, but more need for other kinds of support to manage their risk of COVID-19 exposure. This could include measures such as provision of personal protective equipment and sanitation facilities, safer transport options, shielding policies to protect the most vulnerable or workplace adaptations to allow proper distancing, for example, by operating at reduced capacity.

This analysis also does not include people outside the labour force and does not consider outcomes at the household level. The makeup of households varies considerably across countries; and there may be increased need for social protection schemes untied to employment in countries where a large proportion of the population is outside the labour force.

This analysis focuses on workplace closures and does not consider some other important aspects of health and economic risk during the COVID-19 pandemic. We assume all employees receive full earnings if they are ‘essential’ or ‘teleworking’. We do not include the productivity loss associated with ill health, nor do we consider inequality of access to paid sick leave, which is likely to have a substantial impact. We also do not consider productivity impact of caring responsibilities, which is most likely substantial where schools are closed.^47^ In many settings, the burden of caring responsibilities falls disproportionately on women, and so further investigation into the impact of COVID-19 on gender equity is also important.^48^

These estimates could be applied to an epidemiological model linked to an economic impact model to help understand the differential infection rates across SES quintiles, and trade-offs between aggregate impact and the distribution of impact. Some further adjustment would be required for this application. For example, we do not consider any differential in exposure for those who are working outside the home, although certain occupations would likely have higher risk of infection. We also do not consider personal compliance with ‘stay at home’ orders stemming from other factors, such as trust in institutions or personal risk aversion.

In many countries, changes in the workforce and consumer behaviour have caused substantial macroeconomic changes. Consumer demand has changed due to a combination of NPIs, reduced disposable income and individual choice to limit COVID-19 exposure. Changes in private consumption will affect employment even if there is no official lockdown, for example, through reduced demand leading to business failures and unemployment. Deaths and illness due to COVID-19 have also caused reductions in available labour, and there is evidence that many people have chosen to take early retirement or otherwise leave the workforce.^49^ As such, some of the income loss that we attribute to lockdown policies may have occurred anyway given wider changes in the macro-economy.

Our analysis should be taken as indicative of potential policy implications rather than as definitive evidence of outcomes for the countries included. We used globally available summary data on the workforce in each country and used occupation as a proxy indicator for SES. These limitations highlight an urgent need for more countrylevel data on the impact of non-pharmaceutical interventions on economic and/or health risk. Where such country-level data are available, our results are in line with existing estimates. In Chile and Italy, recent analysis has found that people with lower SES were less able to socially distance during lockdown, while in the UK and Germany lower SES groups were more exposed to COVID-induced ^28, 50–52^ financial hardship. Although there is evidence of inequitable social and economic impacts of COVID-19 in several low-income and middle-income countries, loss of income and ability to socially distance are largely not quantified across income quintiles, making it challenging for policy makers in low-income and middleincome countries to explicitly evaluate trade-offs in their planning and decision-making.^53–55^ National surveys on changes in income, access to social protection, ability and willingness to socially distance would improve the research informing policy in these countries.

## Conclusion

Non-pharmaceutical interventions including national lockdowns have been used by many countries to mitigate the detrimental health and economic effects of the COVID-19 pandemic, which has fallen most heavily on the poor. These lockdown measures have necessarily been accompanied by social protection measures. Evaluating these measures in advance has relied on epidemiological and economic models, most of which are not able to evaluate distributional as well as aggregate impact, the absence of which may lead to a lack of consideration of inequities. Our analysis demonstrates a method that uses globally available data to inform policy makers on where economic risks associated with lockdown may be most critical, and where social protection can be most effectively introduced. Where social protection is unavailable, our work suggests that national lockdowns are likely to be regressive both in their impact on loss of income and in their impact on risk of exposure to COVID-19, across a range of different country settings. But data on the resulting inequity takes time to emerge.

There is substantial policy debate in many countries focusing on the trade-offs between health risk and economic risk with NPIs. Our analysis shows that different sections of the population experience these trade-offs differently, and in many cases these two risks are overlapping. Our results underscore the importance of removing barriers to access to less stringent measures such as improved access to testing and tracing, isolation and targeted screening, improved resources for shielding and vaccination. Although not replacements for broader population-level social distancing measures, these measures may help to limit the need for stringent lockdowns by slowing spread of COVID-19 without impacting individual incomes and can improve equity. Where more stringent measures are needed when COVID-19 infection rates are high, robust socioeconomic support will likely be needed to offset losses in income and enable people to socially distance.

## Supplementary Material

Supplementary Materials This content has been supplied by the author(s). It has not been vetted by BMJ Publishing Group Limited (BMJ) and may not have been peer-reviewed. Any opinions or recommendations discussed are solely those of the author(s) and are not endorsed by BMJ. BMJ disclaims all liability and responsibility arising from any reliance placed on the content. Where the content includes any translated material, BMJ does not warrant the accuracy and reliability of the translations (including but not limited to local regulations, clinical guidelines, terminology, drug names and drug dosages), and is not responsible for any error and/or omissions arising from translation and adaptation or otherwise.

## Figures and Tables

**Table 1:** Outcomes, concentration indices and costs of social protection programmes

	People losing income		People unable to socially distance		Total projected cost of social protection programmes (billions) (% GDP)
Social protection coverage	Number of people (% labour force)	Concentration index (SE)	Number of people (% labour force)	Concentration index (SE)	
20% coverage (base case)					
Chile	1.6M (24.6%)	0.02 (0.06)	4.6M (70.2%)	−0.19 (0.05)	5.06 (1.1%)
Georgia	0.3M (37.0%)	−0.12 (0.03)	0.5M (59.3%)	−0.11 (0.04)	0.37 (0.6%)
Pakistan	13.2M (50.9%)	−0.09 (0.09)	15.6M (60.0%)	−0.13 (0.03)	1.07 (0.1%)
Philippines	13.8M (49.3%)	−0.09 (0.09)	17.8M (63.6%)	−0.10 (0.02)	7.03 (0.7%)
South Africa	7.9M (49.1%)	−0.15 (0.06)	10.4M (64.8%)	−0.10 (0.03)	2.40 (0.3%)
UK	11.2M (39.9%)	−0.24 (0.06)	12.5M (44.7%)	−0.02 (0.06)	25.28 (0.8%)
50% coverage					
Chile	1.0M (15.4%)	0.02 (0.06)	4.1M (63.6%)	−0.19 (0.05)	12.64 (2.7%)
Georgia	0.2M (23.1%)	−0.12 (0.03)	0.5M (50.7%)	−0.07 (0.03)	0.92 (1.6%)
Pakistan	8.3M (31.8%)	−0.09 (0.09)	12.2M (46.8%)	−0.11 (0.02)	2.68 (0.3%)
Philippines	8.6M (30.8%)	−0.09 (0.09)	14.1M (50.4%)	−0.08 (0.04)	17.58 (1.8%)
South Africa	4.9M (30.7%)	−0.15 (0.06)	8.0M (50.2%)	−0.07 (0.03)	5.99 (0.8%)
UK	7.0M (25.0%)	−0.24 (0.06)	10.9M (39.0%)	0.03 (0.05)	63.19 (1.9%)
80% coverage					
Chile	0.4M (6.2%)	0.02 (0.06)	3.7M (56.9%)	−0.20 (0.05)	20.22 (4.2%)
Georgia	0.1M (9.2%)	−0.12 (0.03)	0.4M (42.1%)	0.01 (0.02)	1.47 (2.5%)
Pakistan	3.3M (12.7%)	−0.09 (0.09)	8.8M (33.7%)	−0.09 (0.04)	4.29 (0.4%)
Philippines	3.5M (12.3%)	−0.09 (0.09)	10.4M (37.1%)	−0.05 (0.08)	28.12 (2.8%)
South Africa	2.0M (12.3%)	−0.15 (0.06)	5.7M (35.7%)	−0.01 (0.04)	9.58 (1.3%)
UK	2.8M (10.0%)	−0.24 (0.06)	9.3M (33.2%)	0.09 (0.05)	101.11 (3.1%)

GDP, gross domestic product; M, million; SE, standard error.

## Data Availability

Data sharing not applicable as no datasets generated and/or analysed for this study. This modelling study used published or publicly available data. The data used and the sources are described in this article and in the appendix. No primary data were collected for this study. Access to the model described in the study can be requested by contacting sedona.sweeney@lshtm.ac.uk.

## Key questions

### What is already known?

► The COVID-19 pandemic is exacerbating existing structural inequalities around the world.
► There are few tools available to rapidly assess potential equity impacts and social protection needs when evaluating policy options, especially where household-level data are lacking.

### What are the new findings?

► We find the overall numbers of people losing income and/or unable to socially distance in the absence of social protection were likely to be high in all countries.
► People in lower socioeconomic status quintiles were consistently more likely to have been exposed to greater health and/or economic risk during lockdown across all countries studied.

### What do the new findings imply?

► Equity impacts of interventions can be evaluated alongside their macro-economic and epidemiological impact using publicly available data.
► Use of this model can inform further decisions on non-pharmaceutical interventions and help to identify populations in need of social protection.

## References

[1] Blavatnik School of Government Coronavirus government response tracker.

[2] Republic of the Philippines inter-agency task force for the management of emerging infectious diseases (2020). Omnibus guidelines on the implementation of the community quarantine in the Philippines.

[3] UK Ministry of Housing Communities & Local Government (2020). Closing certain businesses and venues in England.

[4] UK Cabinet Office Closing certain businesses and venues in England.

[5] Government of Georgia (2020). Report on the actions taken by the government of Georgia against COVID-19.

[6] Bravo D, Castillo E, Hughes E Estudio longitudinal Empleo-Covid19: Datos de empleo en tiempo real. Seminario Y Conferencia de Prensa.

[7] Dehning J, Zierenberg J, Spitzner FP (2020). Inferring change points in the spread of COVID-19 reveals the effectiveness of interventions. Science.

[8] Walker PGT, Whittaker C, Watson OJ (2020). The impact of COVID-19 and strategies for mitigation and suppression in low- and middleincome countries. Science.

[9] Lenzer J (2020). Covid-19: Experts debate merits of lockdowns versus ‘focused protection’. BMJ.

[10] Marmot M (2005). Social determinants of health inequalities. Lancet.

[11] Lönnroth K, Jaramillo E, Williams BG (2009). Drivers of tuberculosis epidemics: the role of risk factors and social determinants. Soc Sci Med.

[12] Ceylan RF, Ozkan B (2020). The economic effects of epidemics: from SARS and MERS to COVID-19. Res J Adv Humanit.

[13] Ambrus A, Field E, Gonzalez R (2020). Loss in the time of cholera: long-run impact of a disease epidemic on the urban landscape. Am Econ Rev.

[14] Pedrazzoli D, Wingfield T (2021). Biosocial strategies to address the socioeconomic determinants and consequences of the TB and COVID-19 pandemics. Am J Trop Med Hyg.

[15] Williamson EJ, Walker AJ, Bhaskaran K (2020). Factors associated with COVID-19-related death using OpenSAFELY. Nature.

[16] Marmot M, Allen J COVID-19: exposing and amplifying inequalities. J Epidemiol Community Health.

[17] Patel JA, Nielsen FBH, Badiani AA (2020). Poverty, inequality and COVID-19: the forgotten vulnerable. Public Health.

[18] Bonaccorsi G, Pierri F, Cinelli M (2020). Economic and social consequences of human mobility restrictions under COVID-19. Proc Natl Acad Sci U S A.

[19] Glover RE, van Schalkwyk MCI, Akl EA (2020). A framework for identifying and mitigating the equity harms of COVID-19 policy interventions. J Clin Epidemiol.

[20] Martin A, Markhvida M, Hallegatte S (2020). Socio-economic impacts of COVID-19 on household consumption and poverty. Econ Disaster Clim Chang.

[21] Brum M, De Rosa M (2021). Too little but not too late: nowcasting poverty and cash transfers’ incidence during COVID-19’s crisis. World Dev.

[22] The World Bank (2021). World bank country and lending groups.

[23] Vassall A, Sweeney S, Barasa E (2020). Integrating economic and health evidence to inform Covid-19 policy in low-and middle-income countries [version 1; peer review: 1 approved with reservations] report. Wellcome Open Research.

[24] International Labour Organization (2012). International standard classification of occupations: structure, group definitions and correspondence tables.

[25] International Labour Organization Unemployment of previously employed persons by sex and former occupation (thousands) - annual.

[26] International Labour Organization Employment by sex and occupation - ILO modelled estimates, (thousands) - annual, 2019.

[27] ISCO International standard classification of occupations part 4: correspondence tables.

[28] International Labour Organization Employees by economic activity and occupation (thousands) - Annual.

[29] Jarpa Tomás Molina (2020). Sector minero opera con cerca del 50% de su dotación presencial y actores proyectan cuánto caería actividad en 2020. https://www.emol.com/noticias/Economia/2020/06/25/990110/Sector-minero-produccion-dotacion.html.

[30] Dingel JI, Neiman B (2020). How many jobs can be done at home?. J Public Econ.

[31] Winskill P, Whittaker C, Walker P (2020). Report 22 : Equity in response to the COVID-19 pandemic : an assessment of the direct and indirect impacts on disadvantaged and vulnerable populations in low- and lower middle-income countries.

[32] Brussevich M, Dabla-Norris E, Khalid S (2020). Who will bear the brunt of lockdown policies? Evidence from tele-workability measures across countries. IMF Work Pap.

[33] Sánchez-Páramo C, Narayan A (2020). Impact of COVID-19 on households: what do phone surveys tell us?. World Bank Blogs.

[34] Demirgüç-Kunt A, Klapper L, Singer D (2020). The global Findex database 2017: measuring financial inclusion and opportunities to expand access to and use of financial services. World Bank Econ Rev.

[35] Ganzeboom HBG (2010). A new international socio-economic index [ISEI] of occupational status for the international standard classification of occupation 2008 [ISCO-08].

[36] Ganzeboom HBG, Treiman DJ (1996). Internationally comparable measures of occupational status for the 1988 international standard classification of occupations. Soc Sci Res.

[37] Treiman DJ (1977). Occupational prestige in comparative perspective.

[38] Kakwani NC (1977). Applications of Lorenz curves in economic analysis. Econometrica.

[39] Kakwani N, Wagstaff A, Van Doorslaer E (1997). Socioeconomic inequalities in health: measurement, computation, and statistical inference. J Econom.

[40] Wagstaff A, O’Donnell O, van DE (2007). Quantitative techniques for health equity analysis.

[41] Chen ZA, Chen Z CONCINDC: Stata module to calculate concentration index with both individual and grouped data.

[42] GNI per capita, PPP (current international $).

[43] OECD (2020). Social spending (indicator.

[44] Torres-Rueda S, Sweeney S, Bozzani F (2020). The health sector cost of different policy responses to COVID-19 in low- and middle-income countries. medRxiv.

[45] COVID-19 cost tracker - National Audit Office (NAO).

[46] International Labour Organization (2020). Social protection responses to the COVID-19 crisis: country responses and policy considerations.

[47] Phillips D, Paul G, Fahy M (2020). The invisible workforce during the COVID-19 pandemic: family carers at the frontline. HRB Open Res.

[48] Hupkau C, Petrongolo B (2020). Work, care and gender during the COVID-19 crisis. Fisc Stud.

[49] Coibion O, Gorodnichenko Y, Weber M (2020). Labor markets during the COVID-19 crisis: a preliminary view.

[50] Gozzi N, Tizzoni M, Chinazzi M (2021). Estimating the effect of social inequalities on the mitigation of COVID-19 across communities in Santiago de Chile. Nat Commun.

[51] Adams-Prassl A, Boneva T, Golin M (2020). Inequality in the impact of the coronavirus shock: evidence from real time surveys. J Public Econ.

[52] Witteveen D (2020). Sociodemographic inequality in exposure to COVID-19-induced economic hardship in the United Kingdom. Res Soc Stratif Mobil.

[53] Shadmi E, Chen Y, Dourado I (2020). Health equity and COVID-19: global perspectives. Int J Equity Health.

[54] Quaife M, Van Zandvoort K, Gimma A (2020). The impact of COVID-19 control measures on social contacts and transmission in Kenyan informal settlements. BMC Medicine.

[55] Understanding the impact of the COVID-19 outbreak on the Nigerian economy.

