# Supplementary material for: Exploring equity in health and poverty impacts of control measures for SARS-CoV-2 in six countries
Source: BMJ Glob Health. Author manuscript; Available in PMC 2021 Jul 27. (PMC8159665; doi:10.1136/bmjgh-2021-005521)
Supplement: Supplementary Materials — This content has been supplied by the author(s). It has not been vetted by BMJ Publishing Group Limited (BMJ) and may not have been peer-reviewed. Any opinions or recommendations discussed are solely those of the author(s) and are not endorsed by BMJ. BMJ disclaims all liability and responsibility arising from any reliance placed on the content. Where the content includes any translated material, BMJ does not warrant the accuracy and reliability of the translations (including but not limited to local regulations, clinical guidelines, terminology, drug names and drug dosages), and is not responsible for any error and/or omissions arising from translation and adaptation or otherwise. [file EMS126940-supplement-Supplementary_Materials.pdf]

Supplemental File 1 Supplementary Results Tables

Supplementary Table 1 Total people in ISCO-08 occupation codes by country

	**Quintile 1** n (thousands), %	**Quintile 2** n (thousands), %	**Quintile 3** n (thousands), %	**Quintile 4** n (thousands), %	**Quintile 5** n (thousands), %	**Total** n (thousands), %
**Chile**						
Total people in the labour force	1,293	1,293	1,292	1,293	1,291	6,463
Total unemployed people	115 (9%)	104 (8%)	114 (9%)	125 (10%)	74 (6%)	532 (8%)
Total people in 'essential' jobs	1,016 (79%)	772 (60%)	899 (70%)	355 (27%)	348 (27%)	3,391 (52%)
Total people in 'closed' jobs	93 (7%)	185 (14%)	162 (13%)	112 (9%)	14 (1%)	567 (9%)
Total people teleworking	(0%)	17 (1%)	8 (1%)	434 (34%)	622 (48%)	1,082 (17%)
Total people unable to telework	70 (5%)	215 (17%)	110 (8%)	267 (21%)	231 (18%)	893 (14%)
**Georgia**						
Total people in the labour force	336	334	334	334	333	1,671
Total unemployed people	217 (65%)	167 (50%)	179 (54%)	146 (44%)	122 (37%)	831 (50%)
Total people in 'essential' jobs	44 (13%)	61 (18%)	60 (18%)	87 (26%)	81 (24%)	333 (20%)
Total people in 'closed' jobs	30 (9%)	81 (24%)	23 (7%)	40 (12%)	19 (6%)	193 (12%)
Total people teleworking	(0%)	1 (0%)	22 (7%)	38 (11%)	98 (29%)	160 (10%)
Total people unable to telework	45 (13%)	25 (7%)	49 (15%)	23 (7%)	12 (4%)	154 (9%)
**South Africa**						
Total people in the labour force	3,222	3,221	3,222	3,221	3,223	16,108
Total unemployed people	766 (24%)	916 (28%)	209 (6%)	277 (9%)	165 (5%)	2,332 (14%)
Total people in 'essential' jobs	1,026 (32%)	335 (10%)	838 (26%)	1,002 (31%)	978 (30%)	4,179 (26%)
Total people in 'closed' jobs	917 (28%)	753 (23%)	352 (11%)	687 (21%)	541 (17%)	3,250 (20%)
Total people teleworking	(0%)	41 (1%)	64 (2%)	706 (22%)	1,228 (38%)	2,040 (13%)
Total people unable to telework	514 (16%)	1,176 (37%)	1,759 (55%)	549 (17%)	310 (10%)	4,308 (27%)
**Pakistan**						
Total people in the labour force	5,303	5,301	5,300	5,303	5,302	26,510
Total unemployed people	121 (2%)	113 (2%)	160 (3%)	189 (4%)	248 (5%)	831 (3%)
Total people in 'essential' jobs	1,836 (35%)	1,386 (26%)	633 (12%)	1,144 (22%)	1,448 (27%)	6,447 (24%)

Total people in 'closed' jobs	521 (10%)	494 (9%)	1,915 (36%)	852 (16%)	752 (14%)	4,533 (17%)
Total people teleworking	1 (0%)	137 (3%)	181 (3%)	329 (6%)	2,322 (44%)	2,971 (11%)
Total people unable to telework	2,823 (53%)	3,171 (60%)	2,411 (45%)	2,789 (53%)	532 (10%)	11,727 (44%)
**Philippines**						
Total people in the labour force	5,589	5,587	5,588	5,587	5,586	27,938
Total unemployed people	167 (3%)	185 (3%)	249 (4%)	219 (4%)	114 (2%)	934 (3%)
Total people in 'essential' jobs	2,150 (38%)	1,741 (31%)	524 (9%)	1,190 (21%)	2,314 (41%)	7,918 (28%)
Total people in 'closed' jobs	1,271 (23%)	1,203 (22%)	2,525 (45%)	1,307 (23%)	739 (13%)	7,046 (25%)
Total people teleworking	(0%)	63 (1%)	144 (3%)	817 (15%)	1,765 (32%)	2,788 (10%)
Total people unable to telework	2,001 (36%)	2,396 (43%)	2,146 (38%)	2,054 (37%)	655 (12%)	9,251 (33%)
**United Kingdom**						
Total people in the labour force	5,658	5,658	5,657	5,658	5,657	28,288
Total unemployed people	287 (5%)	231 (4%)	163 (3%)	92 (2%)	63 (1%)	837 (3%)
Total people in 'essential' jobs	1,409 (25%)	893 (16%)	1,996 (35%)	1,550 (27%)	2,461 (44%)	8,310 (29%)
Total people in 'closed' jobs	1,266 (22%)	1,711 (30%)	1,195 (21%)	735 (13%)	741 (13%)	5,648 (20%)
Total people teleworking	172 (3%)	306 (5%)	1,012 (18%)	2,365 (42%)	2,003 (35%)	5,857 (21%)
Total people unable to telework	2,524 (45%)	2,517 (44%)	1,291 (23%)	915 (16%)	389 (7%)	7,637 (27%)

Supplementary Table 2 Total people in ISCO-08 occupation codes by country

ISCO-08 Occupation code and description	ISEI Score	Total people in the workforce (including employees and unemployed)					
		Chile	Georgia	Pakistan	Philippines	South Africa	UK
**1. Managers**							
11 - Chief executives, senior officials and legislators	69	7.8K	5.1K	115.2K	497.1K	825.4K	103.0K
12 - Administrative and commercial managers	68	46.6K	91.6K	201.3K	257.9K	34.1K	905.3K
13 - Production and specialised services managers	60	42.4K	44.0K	316.6K	226.3K		1260.3K
14 - Hospitality, retail and other services managers	53			25.7K	101.0K	130.4K	871.0K
**2. Professionals**							
21 - Science and engineering professionals	70	204.8K	34.8K	121.6K	256.4K	92.1K	1130.3K
22 - Health professionals	66	109.6K	33.7K	119.0K	290.8K	351.7K	1262.3K
23 - Teaching professionals	63	301.4K	129.8K	1971.2K	1288.8K	286.7K	1403.0K
24 - Business and administration professionals	64	282.0K	91.4K	266.5K	249.7K		2059.6K
25 - Information and communications technology professionals	69			48.4K	141.6K		938.8K
26 - Legal, social and cultural professionals	66			338.8K	129.2K	327.9K	610.8K
**3. Technicians and associate professionals**							
31 - Science and engineering associate professionals	51	131.6K	39.2K	365.1K	300.9K	242.7K	528.3K
32 - Health associate professionals	47	157.9K	45.9K	474.1K	401.0K	313.8K	347.8K
33 - Business and administration associate professionals	52	66.7K	28.5K	631.6K	726.5K	459.9K	1861.5K
34 - Legal, social, cultural, related associate professionals	45	396.1K	89.1K	96.4K	158.0K		747.9K
35 - Information and communications technicians	53			150.5K	125.3K	1152.1K	220.0K
**4. Clerical support workers**							
41 - General and keyboard clerks	41	419.0K	71.4K	490.0K	945.7K	665.5K	219.6K
42 - Customer services clerks	40	246.5K	53.2K	160.8K	1072.1K		1195.2K
43 - Numerical and material recording clerks	43			184.0K	481.2K		648.5K
44 - Other clerical support workers	39			55.0K	159.3K	1909.8K	955.2K
**5. Service and sales workers**							
51 - Personal service workers	30	589.4K	150.7K	1069.5K	1253.7K	951.7K	1017.6K
52 - Sales workers	33	408.0K	80.9K	1661.6K	2525.1K		1691.2K
53 - Personal care workers	26			26.8K	414.7K		2038.9K
54 - Protective services workers	39			927.5K	995.7K		555.5K

**6. Skilled agricultural, forestry and fishery workers**							
61 - Market-oriented skilled agricultural workers	19	113.1K	37.4K	133.2K	1.1K	36.8K	128.6K
62 - Market-oriented skilled forestry, fishery, hunting workers	24	1.3K		20.1K	25.5K		20.7K
63 - Subsistence farmers, fishers, hunters and gatherers	10					686.3K	
**7. Craft and related trades workers**							
71 - Building and related trades workers, excluding electricians	34	338.0K	81.9K	1548.9K	1485.3K	779.8K	452.6K
72 - Metal, machinery and related trades workers	38	269.6K	69.6K	773.2K	575.1K	87.2K	679.3K
73 - Handicraft and printing workers	33	18.6K	7.2K	282.7K	169.9K	269.7K	98.1K
74 - Electrical and electronic trades workers	43	124.0K	63.3K	390.4K	331.7K		264.9K
75 - Food processing, wood working, garment and other craft and related trades	27			2103.6K	547.4K		251.1K
**8. Plant and machine operators, and assemblers**							
81 - Stationary plant and machine operators	29	42.8K	15.8K	1005.7K	342.5K	486.8K	319.0K
82 - Assemblers	29	128.2K	22.3K	12.0K	361.7K		120.0K
83 - Drivers and mobile plant operators	36	400.9K	128.7K	1498.5K	1709.5K	3088.3K	881.2K
**9. Elementary occupations**							
91 - Cleaners and helpers	17	915.8K	171.2K	1028.2K	1991.1K	1477.0K	580.0K
92 - Agricultural, forestry and fishery labourers	15	385.3K	16.3K	2615.1K	3246.3K	1452.8K	50.0K
93 - Labourers in mining, construction, manufacturing, transport	24	315.6K	68.1K	4714.9K	3325.8K		959.3K
94 - Food preparation assistants	20			10.6K	23.3K		23.3K
95 - Street and related sales and service workers	25			555.4K	581.4K		354.3K
96 - Refuse workers and other elementary workers	26				222.1K		534.0K

K, Thousand. ISEI, international socio-economic index.

Supplementary Table 3 Essential workers by country

ISCO-08 Occupation code and description	Chile	Georgia	Pakistan	Philippines	South Africa	UK
**1. Managers**	1.2K	1.2K	29.9K	270.3K	186.4K	25.1K
11 - Chief executives, senior officials and legislators	3.6K	21.4K	52.3K	140.2K	7.7K	220.3K
12 - Administrative and commercial managers	13.2K	10.3K	82.2K	123.1K		306.6K
13 - Production and specialised services managers			6.7K	54.9K	29.5K	211.9K
14 - Hospitality, retail and other services managers						
**2. Professionals**	37.1K	7.4K	10.9K	74.6K	20.7K	375.1K
21 - Science and engineering professionals	83.5K	22.3K	115.2K	286.2K	336.8K	1252.0K
22 - Health professionals	54.6K	16.3K	91.4K	260.8K	29.6K	309.1K
23 - Teaching professionals	51.0K	19.4K	23.9K	72.7K		683.4K
24 - Business and administration professionals			4.2K	41.0K		306.0K
25 - Information and communications technology professionals			9.8K	23.3K	27.1K	48.0K
26 - Legal, social and cultural professionals						
**3. Technicians and associate professionals**	21.5K	10.4K	164.9K	117.4K	70.4K	187.6K
31 - Science and engineering associate professionals	115.5K	26.6K	450.6K	391.4K	295.4K	340.3K
32 - Health associate professionals	10.9K	7.5K	285.4K	283.4K	133.4K	660.9K
33 - Business and administration associate professionals	64.7K	21.2K	38.9K	54.1K		208.1K
34 - Legal, social, cultural, related associate professionals			68.0K	48.9K	334.2K	78.1K
35 - Information and communications technicians						
**4. Clerical support workers**	75.2K	17.8K	211.0K	246.6K	171.4K	72.6K
41 - General and keyboard clerks	44.2K	11.5K	60.6K	257.6K		350.5K
42 - Customer services clerks			79.2K	125.5K		214.5K
43 - Numerical and material recording clerks			20.7K	38.3K	412.8K	280.2K
44 - Other clerical support workers						
**5. Service and sales workers**	226.2K	30.6K	153.3K	129.5K	62.3K	73.5K
51 - Personal service workers	201.2K	16.4K	238.2K	253.4K		122.1K
52 - Sales workers			15.8K	199.4K		807.3K
53 - Personal care workers			156.8K	117.8K		220.0K
54 - Protective services workers						
**6. Skilled agricultural, forestry and fishery workers**	95.8K	5.6K	81.8K	0.7K	34.5K	111.0K

61 - Market-oriented skilled agricultural workers	1.1K		8.5K	16.3K		5.7K
62 - Market-oriented skilled forestry, fishery, hunting workers					679.0K	
63 - Subsistence farmers, fishers, hunters and gatherers						
**7. Craft and related trades workers**	253.7K	10.9K	105.4K	69.7K	30.3K	45.8K
71 - Building and related trades workers, excluding electricians	199.8K	9.2K	52.6K	27.0K	3.4K	68.7K
72 - Metal, machinery and related trades workers	14.0K	1.0K	19.2K	8.0K	10.5K	9.9K
73 - Handicraft and printing workers	91.9K	8.5K	26.6K	15.8K		29.0K
74 - Electrical and electronic trades workers			145.7K	26.2K		25.4K
75 - Food processing, wood working, garment and other craft and related trades						
**8. Plant and machine operators, and assemblers**	33.8K	1.6K	133.6K	25.7K	60.6K	31.9K
81 - Stationary plant and machine operators	101.1K	2.3K	1.6K	27.1K		12.0K
82 - Assemblers	313.0K	20.7K	490.6K	505.8K	841.9K	221.5K
83 - Drivers and mobile plant operators						
**9. Elementary occupations**	705.9K	22.2K	346.5K	743.6K	204.6K	99.4K
91 - Cleaners and helpers	316.1K	2.1K	895.8K	1259.2K	196.6K	7.3K
92 - Agricultural, forestry and fishery labourers	260.6K	8.8K	1578.5K	1259.0K		122.5K
93 - Labourers in mining, construction, manufacturing, transport			3.6K	8.7K		4.0K
94 - Food preparation assistants			187.2K	217.1K		60.8K
95 - Street and related sales and service workers				97.3K		101.6K
96 - Refuse workers and other elementary workers	1.2K	1.2K	29.9K	270.3K	186.4K	25.1K

K, Thousand.

Supplementary Table 4 Closed jobs by country

ISCO-08 Occupation code and description	Chile	Georgia	Pakistan	Philippines	South Africa	UK
**1. Managers**	0.2K	0.5K	7.9K	93.1K	188.5K	21.8K
11 - Chief executives, senior officials and legislators	1.5K	8.7K	13.8K	48.3K	7.8K	192.0K
12 - Administrative and commercial managers	1.3K	4.2K	21.7K	42.4K		267.3K
13 - Production and specialised services managers			1.8K	18.9K	29.8K	184.7K
14 - Hospitality, retail and other services managers						
**2. Professionals**	1.9K	1.7K	15.9K	14.3K	4.3K	77.7K
21 - Science and engineering professionals	0.0B	0.0B	0.0B	0.0B	0.0B	0.0B
22 - Health professionals	2.8K	6.4K	258.3K	71.9K	13.4K	66.2K
23 - Teaching professionals	2.7K	4.5K	34.9K	13.9K		141.5K
24 - Business and administration professionals			6.3K	7.9K		64.5K
25 - Information and communications technology professionals			238.9K	70.5K	191.4K	336.4K
26 - Legal, social and cultural professionals						
**3. Technicians and associate professionals**	2.6K	3.9K	34.3K	57.3K	21.9K	85.1K
31 - Science and engineering associate professionals	0.0B	0.0B	0.0B	0.0B	0.0B	0.0B
32 - Health associate professionals	1.3K	2.9K	59.3K	138.3K	41.5K	300.0K
33 - Business and administration associate professionals	40.1K	25.1K	40.1K	78.5K		409.0K
34 - Legal, social, cultural, related associate professionals			14.1K	23.8K	104.0K	35.5K
35 - Information and communications technicians						
**4. Clerical support workers**	42.7K	10.4K	24.4K	166.2K	187.0K	38.4K
41 - General and keyboard clerks	25.1K	7.7K	8.0K	188.5K		209.1K
42 - Customer services clerks			9.2K	84.6K		113.4K
43 - Numerical and material recording clerks			2.7K	28.0K	536.5K	167.1K
44 - Other clerical support workers						
**5. Service and sales workers**	140.8K	52.2K	870.0K	1041.5K	417.1K	585.1K
51 - Personal service workers	97.5K	27.2K	876.3K	1378.1K		721.1K
52 - Sales workers			5.4K	122.7K		295.9K
53 - Personal care workers			579.8K	651.0K		187.2K
54 - Protective services workers						
**6. Skilled agricultural, forestry and fishery workers**	2.0K	0.0B	0.0B	0.0B	1.2K	0.0B

61 - Market-oriented skilled agricultural workers	0.0K		3.1K	0.0B		4.9K
62 - Market-oriented skilled forestry, fishery, hunting workers					0.0B	
63 - Subsistence farmers, fishers, hunters and gatherers						
**7. Craft and related trades workers**	33.5K	2.6K	136.1K	138.7K	87.5K	68.2K
71 - Building and related trades workers, excluding electricians	12.8K	2.2K	68.0K	53.7K	9.8K	102.3K
72 - Metal, machinery and related trades workers	2.4K	0.2K	24.8K	15.9K	30.2K	14.8K
73 - Handicraft and printing workers	2.4K	2.0K	34.3K	31.0K		39.9K
74 - Electrical and electronic trades workers			174.6K	49.9K		38.2K
75 - Food processing, wood working, garment and other craft and related trades						
**8. Plant and machine operators, and assemblers**	4.3K	0.2K	34.7K	40.9K	44.2K	49.8K
81 - Stationary plant and machine operators	12.9K	0.3K	0.4K	43.2K		18.7K
82 - Assemblers	40.4K	1.7K	51.8K	192.6K	270.9K	114.0K
83 - Drivers and mobile plant operators						
**9. Elementary occupations**	93.5K	21.8K	121.8K	465.0K	547.0K	234.8K
91 - Cleaners and helpers	0.0B	1.2K	251.8K	742.1K	516.1K	15.9K
92 - Agricultural, forestry and fishery labourers	1.7K	5.0K	453.9K	760.3K		305.9K
93 - Labourers in mining, construction, manufacturing, transport			1.0K	5.3K		7.4K
94 - Food preparation assistants			53.5K	132.9K		113.0K
95 - Street and related sales and service workers				35.1K		20.9K
96 - Refuse workers and other elementary workers	0.2K	0.5K	7.9K	93.1K	188.5K	21.8K

Philippines

The Philippines reported its first case of COVID-19 on January 30, 2020. Starting March 16, the government instituted Enhanced Community Quarantine (ECQ). ECQ, the strictest of all levels was initially planned to end on April 30, but was subsequently extended to May 15 in selected areas. During ECQ, the medical sector, agricultural sector, delivery and courier services, as well as manufacturing and supply-chain activities related to these sectors have been maintained at full capacity. Establishments related in the provision of essential goods and services such as public markets, grocery stores, and the like; as well as media establishments, are allowed to operate at 50% capacity. Financial services such as banks, other medical and dental services, water supply and sanitation services, and telecommunications and utilities, and other services were limited to a skeletal workforce.

On June 1, the government moved Metro Manila and other high-risk areas from a “modified enhanced community quarantine” to a “general community quarantine,” which allows more businesses to reopen and mass transportation to resume in phases. As of writing, Cebu City, which had been under “enhanced community quarantine” from June 16 to July 15, has transitioned to a less restrictive “modified enhanced community quarantine.

Percent of jobs that were essential during lockdown

		A. Agriculture; forestry and fishing	B. Mining and quarrying	C. Manufacturing	D. Electricity; gas, steam and air …	E. Water supply; sewerage, waste management and remediation …	F. Construction	G. Wholesale and retail trade; repair of motor vehicles and …	H. Transportation and storage	I. Accommodation and food service …	J. Information and communicat…	K. Financial and insurance activities	L. Real estate activities	M. Professional, scientific and …	N. Administrative and support …	O. Public administration and defence; …	P. Education	Q. Human health and social work activities	R. Arts, entertainment and recreation	S. Other service activities	T. Activities of households as employers; undifferentiated goods- and services-producing activities …
**Isco\ Nace**	**Occupation description**	**A**	**B**	**C**	**D**	**E**	**F**	**G**	**H**	**I**	**J**	**K**	**L**	**M**	**N**	**O**	**P**	**Q**	**R**	**S**	**T**
**11**	11 - Chief executives, senior officials and legislators	100%	10%	10%	10%	10%	0%	0%	50%	0%	25%	25%	0%	0%	0%	100%	0%	100%	0%	0%	0%
**12**	12 - Administrative and commercial managers	100%	10%	10%	10%	10%	0%	0%	50%	0%	25%	25%	0%	0%	0%	100%	0%	100%	0%	0%	0%
**13**	13 - Production and specialised services managers	100%	10%	10%	10%	10%	0%	0%	50%	0%	25%	25%	0%	0%	0%	100%	0%	100%	0%	0%	0%
**14**	14 - Hospitality, retail and other services managers	100%	10%	10%	10%	10%	0%	0%	50%	0%	25%	25%	0%	0%	0%	100%	0%	100%	0%	0%	0%
**21**	21 - Science and engineering professionals	100%	10%	10%	10%	10%	0%	0%	50%	0%	25%	25%	0%	0%	0%	100%	0%	100%	0%	0%	0%
**22**	22 - Health professionals	100%	100%	100%	100%	100%	100%	100%	100%	100%	100%	100%	100%	100%	100%	100%	100%	100%	100%	100%	100%
**23**	23 - Teaching professionals	100%	10%	10%	10%	10%	0%	0%	0%	0%	25%	25%	0%	0%	0%	100%	0%	0%	0%	0%	0%
**24**	24 - Business and administration professionals	100%	10%	10%	10%	10%	0%	0%	50%	0%	25%	25%	0%	0%	0%	100%	0%	100%	0%	0%	0%
**25**	25 - Information and communications technology professionals	100%	10%	10%	10%	10%	0%	0%	0%	0%	25%	25%	0%	0%	0%	100%	0%	100%	0%	0%	0%
**26**	26 - Legal, social and cultural professionals	100%	0%	0%	0%	0%	0%	0%	0%	0%	0%	0%	0%	0%	0%	100%	0%	0%	0%	0%	0%
**31**	31 - Science and engineering associate professionals	100%	10%	10%	10%	10%	0%	0%	50%	0%	25%	25%	0%	0%	0%	100%	0%	100%	0%	0%	0%
**32**	32 - Health associate professionals	100%	100%	100%	100%	100%	100%	100%	100%	100%	100%	100%	100%	100%	100%	100%	100%	100%	100%	100%	100%
**33**	33 - Business and administration associate professionals	100%	10%	10%	10%	10%	0%	0%	50%	0%	25%	25%	0%	0%	0%	100%	0%	100%	0%	0%	0%
**34**	34 - Legal, social, cultural and related associate professionals	100%	0%	0%	0%	0%	0%	0%	0%	0%	0%	0%	0%	0%	0%	100%	0%	100%	0%	0%	0%
**35**	35 - Information and communications technicians	100%	10%	10%	10%	10%	0%	0%	50%	0%	25%	25%	0%	0%	0%	100%	0%	100%	0%	0%	0%
**41**	41 - General and keyboard clerks	100%	10%	10%	10%	10%	0%	0%	50%	0%	25%	25%	0%	0%	0%	100%	0%	100%	0%	0%	0%
**42**	42 - Customer services clerks	100%	10%	10%	10%	10%	0%	0%	0%	0%	25%	25%	0%	0%	0%	100%	0%	100%	0%	0%	0%
**43**	43 - Numerical and material recording clerks	100%	10%	10%	10%	10%	0%	0%	50%	0%	25%	25%	0%	0%	0%	100%	0%	100%	0%	0%	0%
**44**	44 - Other clerical support workers	100%	10%	10%	10%	10%	0%	0%	0%	0%	25%	25%	0%	0%	0%	100%	0%	100%	0%	0%	0%
**51**	51 - Personal service workers	100%	0%	0%	0%	0%	0%	0%	0%	0%	25%	0%	0%	0%	0%	100%	0%	10%	0%	0%	0%
**52**	52 - Sales workers	100%	0%	0%	0%	0%	0%	0%	0%	0%	0%	0%	0%	0%	0%	100%	0%	0%	0%	0%	0%
**53**	53 - Personal care workers	100%	10%	10%	10%	10%	0%	100%	0%	0%	0%	25%	0%	0%	0%	100%	0%	100%	0%	0%	0%
**54**	54 - Protective services workers	100%	10%	10%	10%	10%	0%	0%	0%	0%	25%	25%	0%	0%	0%	100%	0%	100%	0%	0%	0%
**61**	61 - Market-oriented skilled agricultural workers	100%	10%	10%	10%	10%	0%	0%	0%	0%	25%	25%	100%	100%	100%	100%	0%	0%	100%	100%	100%
**62**	62 - Market-oriented skilled forestry, fishery and hunting workers	100%	10%	10%	10%	10%	0%	0%	0%	0%	25%	25%	0%	0%	0%	100%	0%	0%	0%	0%	0%
**63**	63 - Subsistence farmers, fishers, hunters and gatherers	100%	100%	100%	100%	100%	100%	100%	100%	100%	100%	100%	100%	100%	100%	100%	100%	100%	100%	100%	100%
**71**	71 - Building and related trades workers, excluding electricians	100%	10%	10%	10%	10%	0%	0%	0%	0%	25%	25%	0%	0%	0%	100%	0%	0%	0%	0%	0%
**72**	72 - Metal, machinery and related trades workers	100%	10%	10%	10%	10%	0%	0%	0%	0%	25%	25%	0%	0%	0%	100%	0%	0%	0%	0%	0%
**73**	73 - Handicraft and printing workers	100%	10%	10%	10%	10%	0%	0%	0%	0%	25%	25%	0%	0%	0%	100%	0%	0%	0%	0%	0%
**74**	74 - Electrical and electronic trades workers	100%	10%	10%	10%	10%	0%	0%	0%	0%	25%	25%	0%	0%	0%	100%	0%	100%	0%	0%	0%
**75**	75 - Food processing, wood working, garment and other craft and related trades workers	100%	10%	10%	10%	10%	0%	0%	0%	25%	25%	25%	0%	0%	0%	100%	0%	0%	0%	0%	0%
**81**	81 - Stationary plant and machine operators	100%	10%	10%	10%	10%	0%	0%	0%	0%	25%	25%	0%	0%	0%	100%	0%	0%	0%	0%	0%
**82**	82 - Assemblers	100%	10%	10%	10%	10%	0%	0%	0%	0%	25%	25%	0%	0%	0%	100%	0%	0%	0%	0%	0%
**83**	83 - Drivers and mobile plant operators	100%	10%	10%	10%	10%	0%	0%	50%	50%	25%	25%	0%	0%	0%	100%	0%	50%	0%	0%	0%
**91**	91 - Cleaners and helpers	100%	10%	10%	10%	10%	0%	0%	0%	0%	25%	25%	0%	0%	0%	100%	0%	100%	0%	0%	0%
**92**	92 - Agricultural, forestry and fishery labourers	100%	10%	10%	10%	10%	0%	0%	50%	0%	25%	25%	0%	0%	0%	100%	0%	0%	0%	0%	0%
**93**	93 - Labourers in mining, construction, manufacturing and transport	100%	10%	10%	10%	10%	0%	0%	25%	0%	25%	25%	0%	0%	0%	100%	0%	0%	0%	0%	0%
**94**	94 - Food preparation assistants	100%	10%	10%	10%	10%	0%	100%	25%	50%	25%	25%	0%	0%	0%	100%	0%	100%	0%	0%	0%
**95**	95 - Street and related sales and service workers	100%	10%	10%	10%	10%	0%	0%	0%	0%	25%	25%	0%	0%	0%	100%	0%	100%	0%	0%	0%
**96**	96 - Refuse workers and other elementary workers	100%	10%	10%	10%	10%	0%	0%	0%	0%	25%	25%	0%	0%	0%	100%	0%	100%	0%	0%	0%

Philippines

Percent of jobs that were closed during lockdown

Isco\ Nace	Occupation description	A. Agriculture; forestry and fishing	B. Mining and quarrying	C. Manufacturing	D. Electricity; gas, steam and air	E. Water supply; sewerage, waste management and remediation	F. Construction	G. Wholesale and retail trade; repair of motor vehicles and	H. Transportation and storage	I. Accommodation and food service	J. Information and communicat	K. Financial and insurance activities	L. Real estate activities	M. Professional, scientific and	N. Administrative and support	O. Public administration and defence;	P. Education	Q. Human health and social work activities	R. Arts, entertainment and recreation	S. Other service activities	T. Activities of households as employers; undifferentiated goods- and services-producing activities
		A	B	C	D	E	F	G	H	I	J	K	L	M	N	O	P	Q	R	S	T
11	11 - Chief executives, senior officials and legislators	0%	0%	0%	0%	0%	0%	100%	0%	100%	0%	0%	0%	0%	0%	0%	0%	0%	100%	100%	100%
12	12 - Administrative and commercial managers	0%	0%	0%	0%	0%	0%	100%	0%	100%	0%	0%	0%	0%	0%	0%	0%	0%	100%	100%	100%
13	13 - Production and specialised services managers	0%	0%	0%	0%	0%	0%	100%	0%	100%	0%	0%	0%	0%	0%	0%	0%	0%	100%	100%	100%
14	14 - Hospitality, retail and other services managers	0%	0%	0%	0%	0%	0%	100%	0%	100%	0%	0%	0%	0%	0%	0%	0%	0%	100%	100%	100%
21	21 - Science and engineering professionals	0%	0%	0%	0%	0%	0%	100%	0%	100%	0%	0%	0%	0%	0%	0%	0%	0%	100%	100%	100%
22	22 - Health professionals	0%	0%	0%	0%	0%	0%	0%	0%	0%	0%	0%	0%	0%	0%	0%	0%	0%	0%	0%	0%
23	23 - Teaching professionals	0%	0%	0%	0%	0%	0%	100%	0%	100%	0%	0%	0%	0%	0%	0%	0%	0%	100%	100%	100%
24	24 - Business and administration professionals	0%	0%	0%	0%	0%	0%	100%	0%	100%	0%	0%	0%	0%	0%	0%	0%	0%	100%	100%	100%
25	25 - Information and communications technology professionals	0%	0%	0%	0%	0%	0%	100%	0%	100%	0%	0%	0%	0%	0%	0%	0%	0%	100%	100%	100%
26	26 - Legal, social and cultural professionals	0%	75%	75%	75%	75%	75%	75%	75%	100%	75%	75%	75%	75%	75%	0%	75%	0%	100%	100%	100%
31	31 - Science and engineering associate professionals	0%	0%	0%	0%	0%	0%	100%	0%	100%	0%	0%	0%	0%	0%	0%	0%	0%	100%	100%	100%
32	32 - Health associate professionals	0%	0%	0%	0%	0%	0%	0%	0%	0%	0%	0%	0%	0%	0%	0%	0%	0%	0%	0%	0%
33	33 - Business and administration associate professionals	0%	0%	0%	0%	0%	0%	100%	0%	100%	0%	0%	0%	0%	0%	0%	0%	0%	100%	100%	100%
34	34 - Legal, social, cultural and related associate professionals	0%	75%	75%	75%	75%	75%	75%	75%	100%	75%	75%	75%	75%	75%	0%	75%	0%	100%	100%	100%
35	35 - Information and communications technicians	0%	0%	0%	0%	0%	0%	100%	0%	100%	0%	0%	0%	0%	0%	0%	0%	0%	100%	100%	100%
41	41 - General and keyboard clerks	0%	0%	0%	0%	0%	0%	100%	0%	100%	0%	0%	0%	0%	0%	0%	0%	0%	100%	100%	100%
42	42 - Customer services clerks	0%	0%	0%	0%	0%	0%	100%	0%	100%	0%	0%	0%	0%	0%	0%	0%	0%	100%	100%	100%
43	43 - Numerical and material recording clerks	0%	0%	0%	0%	0%	0%	100%	0%	100%	0%	0%	0%	0%	0%	0%	0%	0%	100%	100%	100%
44	44 - Other clerical support workers	0%	0%	0%	0%	0%	0%	100%	0%	100%	0%	0%	0%	0%	0%	0%	0%	0%	100%	100%	100%
51	51 - Personal service workers	0%	100%	100%	100%	100%	100%	100%	100%	100%	75%	100%	100%	100%	100%	0%	100%	0%	100%	100%	100%
52	52 - Sales workers	0%	75%	75%	75%	75%	75%	75%	75%	0%	0%	0%	100%	100%	100%	0%	75%	0%	100%	100%	100%
53	53 - Personal care workers	0%	0%	0%	0%	0%	0%	0%	0%	100%	100%	0%	0%	0%	0%	0%	0%	0%	100%	100%	100%
54	54 - Protective services workers	0%	0%	0%	0%	0%	0%	100%	0%	100%	0%	0%	0%	0%	0%	0%	0%	0%	100%	100%	100%
61	61 - Market-oriented skilled agricultural workers	0%	0%	0%	0%	0%	0%	100%	0%	100%	0%	0%	0%	0%	0%	0%	0%	0%	0%	0%	0%
62	62 - Market-oriented skilled forestry, fishery and hunting workers	0%	0%	0%	0%	0%	0%	100%	0%	100%	0%	0%	0%	0%	0%	0%	0%	0%	100%	100%	100%
63	63 - Subsistence farmers, fishers, hunters and gatherers	0%	0%	0%	0%	0%	0%	0%	0%	0%	0%	0%	0%	0%	0%	0%	0%	0%	0%	0%	0%
71	71 - Building and related trades workers, excluding electricians	0%	0%	0%	0%	0%	0%	100%	0%	100%	0%	0%	0%	0%	0%	0%	0%	0%	100%	100%	100%
72	72 - Metal, machinery and related trades workers	0%	0%	0%	0%	0%	0%	100%	0%	100%	0%	0%	0%	0%	0%	0%	0%	0%	100%	100%	100%
73	73 - Handicraft and printing workers	0%	0%	0%	0%	0%	0%	100%	0%	100%	0%	0%	0%	0%	0%	0%	0%	0%	100%	100%	100%
74	74 - Electrical and electronic trades workers	0%	0%	0%	0%	0%	0%	100%	0%	100%	0%	0%	0%	0%	0%	0%	0%	0%	100%	100%	100%
75	75 - Food processing, wood working, garment and other craft and related trades workers	0%	0%	0%	0%	0%	0%	100%	0%	0%	0%	0%	0%	0%	0%	0%	100%	0%	100%	100%	100%
81	81 - Stationary plant and machine operators	0%	0%	0%	0%	0%	0%	100%	0%	100%	0%	0%	0%	0%	0%	0%	0%	0%	100%	100%	100%
82	82 - Assemblers	0%	0%	0%	0%	0%	0%	100%	0%	100%	0%	0%	0%	0%	0%	0%	0%	0%	100%	100%	100%
83	83 - Drivers and mobile plant operators	0%	0%	0%	0%	0%	0%	100%	0%	0%	0%	0%	0%	0%	0%	0%	0%	0%	100%	100%	100%
91	91 - Cleaners and helpers	0%	0%	0%	0%	0%	0%	100%	0%	100%	0%	0%	0%	0%	0%	0%	100%	0%	100%	100%	100%
92	92 - Agricultural, forestry and fishery labourers	0%	0%	0%	0%	0%	0%	100%	0%	100%	0%	0%	0%	0%	0%	0%	0%	0%	100%	100%	100%
93	93 - Labourers in mining, construction, manufacturing and transport	0%	0%	0%	0%	0%	0%	100%	0%	100%	0%	0%	0%	0%	0%	0%	0%	0%	100%	100%	100%
94	94 - Food preparation assistants	0%	0%	0%	0%	0%	0%	0%	0%	0%	0%	0%	0%	0%	0%	0%	0%	0%	100%	100%	100%
95	95 - Street and related sales and service workers	0%	0%	0%	0%	0%	0%	100%	0%	100%	0%	0%	0%	0%	0%	0%	0%	0%	100%	100%	100%
96	96 - Refuse workers and other elementary workers	0%	0%	0%	0%	0%	0%	100%	0%	100%	0%	0%	0%	0%	0%	0%	0%	0%	100%	100%	100%

Philippines

Percent of jobs subject to teleworking during lockdown

		A. Agriculture; forestry and fishing	B. Mining and quarrying	C. Manufacturing	D. Electricity; gas, steam and air	E. Water supply; sewerage, waste management and remediation	F. Construction	G. Wholesale and retail trade; repair of motor vehicles and	H. Transportation and storage	I. Accommodation and food service	J. Information and communicat	K. Financial and insurance activities	L. Real estate activities	M. Professional, scientific and	N. Administrative and support	O. Public administration and defence;	P. Education	Q. Human health and social work activities	R. Arts, entertainment and recreation	S. Other service activities	T. Activities of households as employers; undifferentiated goods- and services-producing activities
**Isco\ Nace**	**Occupation description**	**A**	**B**	**C**	**D**	**E**	**F**	**G**	**H**	**I**	**J**	**K**	**L**	**M**	**N**	**O**	**P**	**Q**	**R**	**S**	**T**
**11**	11 - Chief executives, senior officials and legislators	0%	90%	90%	90%	90%	100%	0%	50%	0%	75%	75%	100%	100%	100%	0%	100%	0%	0%	0%	0%
**12**	12 - Administrative and commercial managers	0%	90%	90%	90%	90%	100%	0%	50%	0%	75%	75%	100%	100%	100%	0%	100%	0%	0%	0%	0%
**13**	13 - Production and specialised services managers	0%	90%	90%	90%	90%	100%	0%	50%	0%	75%	75%	100%	100%	100%	0%	100%	0%	0%	0%	0%
**14**	14 - Hospitality, retail and other services managers	0%	90%	90%	90%	90%	100%	0%	50%	0%	75%	75%	100%	100%	100%	0%	100%	0%	0%	0%	0%
**21**	21 - Science and engineering professionals	0%	90%	90%	90%	90%	100%	0%	50%	0%	75%	75%	100%	100%	100%	0%	100%	0%	0%	0%	0%
**22**	22 - Health professionals	0%	0%	0%	0%	0%	0%	0%	0%	0%	0%	0%	0%	0%	0%	0%	0%	0%	0%	0%	0%
**23**	23 - Teaching professionals	0%	90%	90%	90%	90%	100%	0%	100%	0%	75%	75%	100%	100%	100%	0%	100%	100%	0%	0%	0%
**24**	24 - Business and administration professionals	0%	90%	90%	90%	90%	100%	0%	50%	0%	75%	75%	100%	100%	100%	0%	100%	0%	0%	0%	0%
**25**	25 - Information and communications technology professionals	0%	90%	90%	90%	90%	100%	0%	100%	0%	75%	75%	100%	100%	100%	0%	100%	0%	0%	0%	0%
**26**	26 - Legal, social and cultural professionals	0%	25%	25%	25%	25%	25%	25%	25%	0%	25%	25%	25%	25%	25%	0%	25%	100%	0%	0%	0%
**31**	31 - Science and engineering associate professionals	0%	90%	90%	90%	90%	100%	0%	50%	0%	75%	75%	100%	100%	100%	0%	100%	0%	0%	0%	0%
**32**	32 - Health associate professionals	0%	0%	0%	0%	0%	0%	0%	0%	0%	0%	0%	0%	0%	0%	0%	0%	0%	0%	0%	0%
**33**	33 - Business and administration associate professionals	0%	90%	90%	90%	90%	100%	0%	50%	0%	75%	75%	100%	100%	100%	0%	100%	0%	0%	0%	0%
**34**	34 - Legal, social, cultural and related associate professionals	0%	25%	25%	25%	25%	25%	25%	25%	0%	25%	25%	25%	25%	25%	0%	25%	0%	0%	0%	0%
**35**	35 - Information and communications technicians	0%	90%	90%	90%	90%	100%	0%	50%	0%	75%	75%	100%	100%	100%	0%	100%	0%	0%	0%	0%
**41**	41 - General and keyboard clerks	0%	90%	90%	90%	90%	100%	0%	50%	0%	75%	75%	100%	100%	100%	0%	100%	0%	0%	0%	0%
**42**	42 - Customer services clerks	0%	90%	90%	90%	90%	100%	0%	100%	0%	75%	75%	100%	100%	100%	0%	100%	0%	0%	0%	0%
**43**	43 - Numerical and material recording clerks	0%	90%	90%	90%	90%	100%	0%	50%	0%	75%	75%	100%	100%	100%	0%	100%	0%	0%	0%	0%
**44**	44 - Other clerical support workers	0%	90%	90%	90%	90%	100%	0%	100%	0%	75%	75%	100%	100%	100%	0%	100%	0%	0%	0%	0%
**51**	51 - Personal service workers	0%	0%	0%	0%	0%	0%	0%	0%	0%	0%	0%	0%	0%	0%	0%	0%	90%	0%	0%	0%
**52**	52 - Sales workers	0%	25%	25%	25%	25%	25%	25%	25%	100%	100%	100%	0%	0%	0%	0%	25%	100%	0%	0%	0%
**53**	53 - Personal care workers	0%	90%	90%	90%	90%	100%	0%	100%	0%	0%	75%	100%	100%	100%	0%	100%	0%	0%	0%	0%
**54**	54 - Protective services workers	0%	90%	90%	90%	90%	100%	0%	100%	0%	75%	75%	100%	100%	100%	0%	100%	0%	0%	0%	0%
**61**	61 - Market-oriented skilled agricultural workers	0%	90%	90%	90%	90%	100%	0%	100%	0%	75%	75%	0%	0%	0%	0%	100%	100%	0%	0%	0%
**62**	62 - Market-oriented skilled forestry, fishery and hunting workers	0%	90%	90%	90%	90%	100%	0%	100%	0%	75%	75%	100%	100%	100%	0%	100%	100%	0%	0%	0%
**63**	63 - Subsistence farmers, fishers, hunters and gatherers	0%	0%	0%	0%	0%	0%	0%	0%	0%	0%	0%	0%	0%	0%	0%	0%	0%	0%	0%	0%
**71**	71 - Building and related trades workers, excluding electricians	0%	90%	90%	90%	90%	100%	0%	100%	0%	75%	75%	100%	100%	100%	0%	100%	100%	0%	0%	0%
**72**	72 - Metal, machinery and related trades workers	0%	90%	90%	90%	90%	100%	0%	100%	0%	75%	75%	100%	100%	100%	0%	100%	100%	0%	0%	0%
**73**	73 - Handicraft and printing workers	0%	90%	90%	90%	90%	100%	0%	100%	0%	75%	75%	100%	100%	100%	0%	100%	100%	0%	0%	0%
**74**	74 - Electrical and electronic trades workers	0%	90%	90%	90%	90%	100%	0%	100%	0%	75%	75%	100%	100%	100%	0%	100%	0%	0%	0%	0%
**75**	75 - Food processing, wood working, garment and other craft and related trades workers	0%	90%	90%	90%	90%	100%	0%	100%	75%	75%	75%	100%	100%	100%	0%	0%	100%	0%	0%	0%
**81**	81 - Stationary plant and machine operators	0%	90%	90%	90%	90%	100%	0%	100%	0%	75%	75%	100%	100%	100%	0%	100%	100%	0%	0%	0%
**82**	82 - Assemblers	0%	90%	90%	90%	90%	100%	0%	100%	0%	75%	75%	100%	100%	100%	0%	100%	100%	0%	0%	0%
**83**	83 - Drivers and mobile plant operators	0%	90%	90%	90%	90%	100%	0%	50%	50%	75%	75%	100%	100%	100%	0%	100%	50%	0%	0%	0%
**91**	91 - Cleaners and helpers	0%	90%	90%	90%	90%	100%	0%	100%	0%	75%	75%	100%	100%	100%	0%	0%	0%	0%	0%	0%
**92**	92 - Agricultural, forestry and fishery labourers	0%	90%	90%	90%	90%	100%	0%	50%	0%	75%	75%	100%	100%	100%	0%	100%	100%	0%	0%	0%
**93**	93 - Labourers in mining, construction, manufacturing and transport	0%	90%	90%	90%	90%	100%	0%	75%	0%	75%	75%	100%	100%	100%	0%	100%	100%	0%	0%	0%
**94**	94 - Food preparation assistants	0%	90%	90%	90%	90%	100%	0%	75%	50%	75%	75%	100%	100%	100%	0%	100%	0%	0%	0%	0%
**95**	95 - Street and related sales and service workers	0%	90%	90%	90%	90%	100%	0%	100%	0%	75%	75%	100%	100%	100%	0%	100%	0%	0%	0%	0%
**96**	96 - Refuse workers and other elementary workers	0%	90%	90%	90%	90%	100%	0%	100%	0%	75%	75%	100%	100%	100%	0%	100%	0%	0%	0%	0%

Chile

Chile reported its first confirmed COVID-19 case on March 3, 2020. In response to COVID-19, the authorities have implemented a range of measures, including declaration of state of catastrophe (extended till mid-September), travel restrictions, closure of schools, curfews and bans on public gatherings, and a law on teleworking. The authorities have also unveiled measures to support employment and incomes, and provide liquidity.

Percent of jobs that were essential during lockdown

		A. Agriculture; forestry and fishing	B. Mining and quarrying	C. Manufacturing	D. Electricity; gas, steam and air	E. Water supply; sewerage, waste management and remediation	F. Construction	G. Wholesale and **retail trade**; repair of motor vehicles and	H. Transportation and storage	I. Accommodation and food service	J. Information and communicat	K. Financial and insurance activities	L. Real estate activities	M. Professional, scientific and	N. Administrative and support	O. Public administration and defence;	P. Education	Q. Human health and social work activities	R. Arts, entertainment and recreation	S. Other service activities	T. Activities of households as employers; undifferentiated goods- and services-producing activities
**Isco\ Nace**	**Occupation description**	**A**	**B**	**C**	**D**	**E**	**F**	**G**	**H**	**I**	**J**	**K**	**L**	**M**	**N**	**O**	**P**	**Q**	**R**	**S**	**T**
**11**	11 - Chief executives, senior officials and legislators	20%	20%	20%	20%	20%	20%	0%	20%	20%	20%	20%	20%	20%	20%	20%	20%	20%	0%	20%	20%
**12**	12 - Administrative and commercial managers	10%	10%	10%	10%	10%	10%	0%	10%	10%	10%	10%	10%	10%	10%	10%	10%	10%	0%	10%	10%
**13**	13 - Production and specialised services managers	40%	40%	40%	40%	40%	40%	0%	40%	40%	40%	40%	40%	40%	40%	40%	40%	40%	0%	40%	40%
**14**	14 - Hospitality, retail and other services managers	20%	20%	20%	20%	20%	20%	0%	20%	20%	20%	20%	20%	20%	20%	20%	20%	20%	0%	20%	20%
**21**	21 - Science and engineering professionals	20%	20%	20%	20%	20%	20%	0%	20%	20%	20%	20%	20%	20%	20%	20%	20%	20%	0%	20%	20%
**22**	22 - Health professionals	80%	80%	80%	80%	80%	80%	80%	80%	80%	80%	80%	80%	80%	80%	80%	80%	80%	80%	80%	80%
**23**	23 - Teaching professionals	20%	20%	20%	20%	20%	20%	0%	20%	20%	20%	20%	20%	20%	20%	20%	20%	20%	0%	20%	20%
**24**	24 - Business and administration professionals	20%	20%	20%	20%	20%	20%	0%	20%	20%	20%	20%	20%	20%	20%	20%	20%	20%	0%	20%	20%
**25**	25 - Information and communications technology professionals	20%	20%	20%	20%	20%	20%	0%	20%	20%	20%	20%	20%	20%	20%	20%	20%	20%	0%	20%	20%
**26**	26 - Legal, social and cultural professionals	20%	20%	20%	20%	20%	20%	0%	20%	20%	20%	20%	20%	20%	20%	20%	20%	20%	0%	20%	20%
**31**	31 - Science and engineering associate professionals	20%	20%	20%	20%	20%	20%	0%	20%	20%	20%	20%	20%	20%	20%	20%	20%	20%	0%	20%	20%
**32**	32 - Health associate professionals	80%	80%	80%	80%	80%	80%	80%	80%	80%	80%	80%	80%	80%	80%	80%	80%	80%	80%	80%	80%
**33**	33 - Business and administration associate professionals	20%	20%	20%	20%	20%	20%	0%	20%	20%	20%	20%	20%	20%	20%	20%	20%	20%	0%	20%	20%
**34**	34 - Legal, social, cultural and related associate professionals	20%	20%	20%	20%	20%	20%	0%	20%	20%	20%	20%	20%	20%	20%	20%	20%	20%	0%	20%	20%
**35**	35 - Information and communications technicians	20%	20%	20%	20%	20%	20%	0%	20%	20%	20%	20%	20%	20%	20%	20%	20%	20%	0%	20%	20%
**41**	41 - General and keyboard clerks	20%	20%	20%	20%	20%	20%	20%	20%	20%	20%	20%	20%	20%	20%	20%	20%	20%	20%	20%	20%
**42**	42 - Customer services clerks	20%	20%	20%	20%	20%	20%	20%	20%	20%	20%	20%	20%	20%	20%	20%	20%	20%	20%	20%	20%
**43**	43 - Numerical and material recording clerks	20%	20%	20%	20%	20%	20%	20%	20%	20%	20%	20%	20%	20%	20%	20%	20%	20%	20%	20%	20%
**44**	44 - Other clerical support workers	20%	20%	20%	20%	20%	20%	20%	20%	20%	20%	20%	20%	20%	20%	20%	20%	20%	20%	20%	20%
**51**	51 - Personal service workers	50%	50%	50%	50%	50%	50%	30%	50%	50%	50%	50%	50%	50%	50%	50%	50%	50%	30%	50%	50%
**52**	52 - Sales workers	70%	70%	70%	70%	70%	70%	30%	70%	70%	70%	70%	70%	70%	70%	70%	70%	70%	30%	70%	70%
**53**	53 - Personal care workers	50%	50%	50%	50%	50%	50%	50%	50%	50%	50%	50%	50%	50%	50%	50%	50%	50%	50%	50%	50%
**54**	54 - Protective services workers	50%	50%	50%	50%	50%	50%	20%	50%	50%	50%	50%	50%	50%	50%	50%	50%	50%	20%	50%	50%
**61**	61 - Market-oriented skilled agricultural workers	90%	90%	90%	90%	90%	90%	50%	90%	90%	90%	90%	90%	90%	90%	90%	90%	90%	50%	90%	90%
**62**	62 - Market-oriented skilled forestry, fishery and hunting workers	90%	90%	90%	90%	90%	90%	50%	90%	90%	90%	90%	90%	90%	90%	90%	90%	90%	50%	90%	90%
**63**	63 - Subsistence farmers, fishers, hunters and gatherers	100%	100%	100%	100%	100%	100%	100%	100%	100%	100%	100%	100%	100%	100%	100%	100%	100%	100%	100%	100%
**71**	71 - Building and related trades workers, excluding electricians	90%	90%	90%	90%	90%	90%	30%	90%	90%	90%	90%	90%	90%	90%	90%	90%	90%	30%	90%	90%
**72**	72 - Metal, machinery and related trades workers	90%	90%	90%	90%	90%	90%	20%	90%	90%	90%	90%	90%	90%	90%	90%	90%	90%	20%	90%	90%
**73**	73 - Handicraft and printing workers	90%	90%	90%	90%	90%	90%	30%	90%	90%	90%	90%	90%	90%	90%	90%	90%	90%	30%	90%	90%
**74**	74 - Electrical and electronic trades workers	90%	90%	90%	90%	90%	90%	20%	90%	90%	90%	90%	90%	90%	90%	90%	90%	90%	20%	90%	90%
**75**	75 - Food processing, wood working, garment and other craft and related trades workers	90%	90%	90%	90%	90%	90%	30%	90%	90%	90%	90%	90%	90%	90%	90%	90%	90%	30%	90%	90%
**81**	81 - Stationary plant and machine operators	90%	90%	90%	90%	90%	90%	30%	90%	90%	90%	90%	90%	90%	90%	90%	90%	90%	30%	90%	90%
**82**	82 - Assemblers	90%	90%	90%	90%	90%	90%	30%	90%	90%	90%	90%	90%	90%	90%	90%	90%	90%	30%	90%	90%
**83**	83 - Drivers and mobile plant operators	90%	90%	90%	90%	90%	90%	20%	90%	90%	90%	90%	90%	90%	90%	90%	90%	90%	20%	90%	90%
**91**	91 - Cleaners and helpers	90%	90%	90%	90%	90%	90%	45%	90%	90%	90%	90%	90%	90%	90%	90%	90%	90%	45%	90%	90%
**92**	92 - Agricultural, forestry and fishery labourers	90%	90%	90%	90%	90%	90%	90%	90%	90%	90%	90%	90%	90%	90%	90%	90%	90%	90%	90%	90%
**93**	93 - Labourers in mining, construction, manufacturing and transport	90%	90%	90%	90%	90%	90%	95%	90%	90%	90%	90%	90%	90%	90%	90%	90%	90%	95%	90%	90%
**94**	94 - Food preparation assistants	90%	90%	90%	90%	90%	90%	95%	90%	90%	90%	90%	90%	90%	90%	90%	90%	90%	95%	90%	90%
**95**	95 - Street and related sales and service workers	90%	90%	90%	90%	90%	90%	45%	90%	90%	90%	90%	90%	90%	90%	90%	90%	90%	45%	90%	90%
**96**	96 - Refuse workers and other elementary workers	90%	90%	90%	90%	90%	90%	45%	90%	90%	90%	90%	90%	90%	90%	90%	90%	90%	45%	90%	90%

Chile

Percent of jobs that were closed during lockdown

Isco\ Nace	Occupation description	A. Agriculture; forestry and fishing<br>A	B. Mining and quarrying<br>B	C. Manufacturing<br>C	D. Electricity; gas, steam and air<br>D	E. Water supply; sewerage, waste management and remediation<br>E	F. Construction<br>F	G. Wholesale and retail trade; repair of motor vehicles and<br>G	H. Transportation and storage<br>H	I. Accommodation and food service<br>I	J. Information and communicat<br>J	K. Financial and insurance activities<br>K	L. Real estate activities<br>L	M. Professional, scientific and<br>M	N. Administrative and support<br>N	O. Public administration and defence;<br>O	P. Education<br>P	Q. Human health and social work activities<br>Q	R. Arts, entertainment and recreation<br>R	S. Other service activities<br>S	T. Activities of households as employers; undifferentiated goods- and services-producing activities<br>T
**11**	11 - Chief executives, senior officials and legislators	0%	0%	0%	0%	0%	0%	20%	0%	0%	0%	0%	0%	0%	0%	0%	0%	0%	20%	0%	0%
**12**	12 - Administrative and commercial managers	0%	0%	0%	0%	0%	0%	20%	0%	0%	0%	0%	0%	0%	0%	0%	0%	0%	20%	0%	0%
**13**	13 - Production and specialised services managers	0%	0%	0%	0%	0%	0%	20%	0%	0%	0%	0%	0%	0%	0%	0%	0%	0%	20%	0%	0%
**14**	14 - Hospitality, retail and other services managers	0%	0%	0%	0%	0%	0%	20%	0%	0%	0%	0%	0%	0%	0%	0%	0%	0%	20%	0%	0%
**21**	21 - Science and engineering professionals	0%	0%	0%	0%	0%	0%	20%	0%	0%	0%	0%	0%	0%	0%	0%	0%	0%	20%	0%	0%
**22**	22 - Health professionals	0%	0%	0%	0%	0%	0%	0%	0%	0%	0%	0%	0%	0%	0%	0%	0%	0%	0%	0%	0%
**23**	23 - Teaching professionals	0%	0%	0%	0%	0%	0%	20%	0%	0%	0%	0%	0%	0%	0%	0%	0%	0%	20%	0%	0%
**24**	24 - Business and administration professionals	0%	0%	0%	0%	0%	0%	20%	0%	0%	0%	0%	0%	0%	0%	0%	0%	0%	20%	0%	0%
**25**	25 - Information and communications technology professionals	0%	0%	0%	0%	0%	0%	20%	0%	0%	0%	0%	0%	0%	0%	0%	0%	0%	20%	0%	0%
**26**	26 - Legal, social and cultural professionals	10%	10%	10%	10%	10%	10%	20%	10%	10%	10%	10%	10%	10%	10%	10%	10%	10%	20%	10%	10%
**31**	31 - Science and engineering associate professionals	0%	0%	0%	0%	0%	0%	20%	0%	0%	0%	0%	0%	0%	0%	0%	0%	0%	20%	0%	0%
**32**	32 - Health associate professionals	0%	0%	0%	0%	0%	0%	0%	0%	0%	0%	0%	0%	0%	0%	0%	0%	0%	0%	0%	0%
**33**	33 - Business and administration associate professionals	0%	0%	0%	0%	0%	0%	20%	0%	0%	0%	0%	0%	0%	0%	0%	0%	0%	20%	0%	0%
**34**	34 - Legal, social, cultural and related associate professionals	10%	10%	10%	10%	10%	10%	20%	10%	10%	10%	10%	10%	10%	10%	10%	10%	10%	20%	10%	10%
**35**	35 - Information and communications technicians	0%	0%	0%	0%	0%	0%	20%	0%	0%	0%	0%	0%	0%	0%	0%	0%	0%	20%	0%	0%
**41**	41 - General and keyboard clerks	0%	0%	0%	0%	0%	0%	50%	0%	0%	0%	0%	0%	0%	0%	0%	0%	0%	50%	0%	0%
**42**	42 - Customer services clerks	0%	0%	0%	0%	0%	0%	50%	0%	0%	0%	0%	0%	0%	0%	0%	0%	0%	50%	0%	0%
**43**	43 - Numerical and material recording clerks	0%	0%	0%	0%	0%	0%	20%	0%	0%	0%	0%	0%	0%	0%	0%	0%	0%	20%	0%	0%
**44**	44 - Other clerical support workers	0%	0%	0%	0%	0%	0%	20%	0%	0%	0%	0%	0%	0%	0%	0%	0%	0%	20%	0%	0%
**51**	51 - Personal service workers	10%	10%	10%	10%	10%	10%	50%	10%	10%	10%	10%	10%	10%	10%	10%	10%	10%	50%	10%	10%
**52**	52 - Sales workers	10%	10%	10%	10%	10%	10%	50%	10%	10%	10%	10%	10%	10%	10%	10%	10%	10%	50%	10%	10%
**53**	53 - Personal care workers	10%	10%	10%	10%	10%	10%	50%	10%	10%	10%	10%	10%	10%	10%	10%	10%	10%	50%	10%	10%
**54**	54 - Protective services workers	10%	10%	10%	10%	10%	10%	50%	10%	10%	10%	10%	10%	10%	10%	10%	10%	10%	50%	10%	10%
**61**	61 - Market-oriented skilled agricultural workers	0%	0%	0%	0%	0%	0%	50%	0%	0%	0%	0%	0%	0%	0%	0%	0%	0%	50%	0%	0%
**62**	62 - Market-oriented skilled forestry, fishery and hunting workers	0%	0%	0%	0%	0%	0%	50%	0%	0%	0%	0%	0%	0%	0%	0%	0%	0%	50%	0%	0%
**63**	63 - Subsistence farmers, fishers, hunters and gatherers	0%	0%	0%	0%	0%	0%	0%	0%	0%	0%	0%	0%	0%	0%	0%	0%	0%	0%	0%	0%
**71**	71 - Building and related trades workers, excluding electricians	10%	10%	10%	10%	10%	10%	20%	10%	10%	10%	10%	10%	10%	10%	10%	10%	10%	20%	10%	10%
**72**	72 - Metal, machinery and related trades workers	0%	0%	0%	0%	0%	0%	50%	0%	0%	0%	0%	0%	0%	0%	0%	0%	0%	50%	0%	0%
**73**	73 - Handicraft and printing workers	10%	10%	10%	10%	10%	10%	50%	10%	10%	10%	10%	10%	10%	10%	10%	10%	10%	50%	10%	10%
**74**	74 - Electrical and electronic trades workers	0%	0%	0%	0%	0%	0%	20%	0%	0%	0%	0%	0%	0%	0%	0%	0%	0%	20%	0%	0%
**75**	75 - Food processing, wood working, garment and other craft and related trades workers	0%	0%	0%	0%	0%	0%	0%	0%	0%	0%	0%	0%	0%	0%	0%	0%	0%	0%	0%	0%
**81**	81 - Stationary plant and machine operators	10%	10%	10%	10%	10%	10%	20%	10%	10%	10%	10%	10%	10%	10%	10%	10%	10%	20%	10%	10%
**82**	82 - Assemblers	10%	10%	10%	10%	10%	10%	20%	10%	10%	10%	10%	10%	10%	10%	10%	10%	10%	20%	10%	10%
**83**	83 - Drivers and mobile plant operators	10%	10%	10%	10%	10%	10%	20%	10%	10%	10%	10%	10%	10%	10%	10%	10%	10%	20%	10%	10%
**91**	91 - Cleaners and helpers	10%	10%	10%	10%	10%	10%	20%	10%	10%	10%	10%	10%	10%	10%	10%	10%	10%	20%	10%	10%
**92**	92 - Agricultural, forestry and fishery labourers	0%	0%	0%	0%	0%	0%	0%	0%	0%	0%	0%	0%	0%	0%	0%	0%	0%	0%	0%	0%
**93**	93 - Labourers in mining, construction, manufacturing and transport	0%	0%	0%	0%	0%	0%	5%	0%	0%	0%	0%	0%	0%	0%	0%	0%	0%	5%	0%	0%
**94**	94 - Food preparation assistants	0%	0%	0%	0%	0%	0%	5%	0%	0%	0%	0%	0%	0%	0%	0%	0%	0%	5%	0%	0%
**95**	95 - Street and related sales and service workers	0%	0%	0%	0%	0%	0%	20%	0%	0%	0%	0%	0%	0%	0%	0%	0%	0%	20%	0%	0%
**96**	96 - Refuse workers and other elementary workers	0%	0%	0%	0%	0%	0%	20%	0%	0%	0%	0%	0%	0%	0%	0%	0%	0%	20%	0%	0%

Chile

Percent of jobs subject to teleworking during lockdown

Isco\ Nace	Occupation description	A. Agriculture; forestry and fishing	B. Mining and quarrying	C. Manufacturing	D. Electricity; gas, steam and air	E. Water supply; sewerage, waste management and remediation	F. Construction	G. Wholesale and retail trade; repair of motor vehicles and	H. Transportation and storage	I. Accommodation and food service	J. Information and communicat	K. Financial and insurance activities	L. Real estate activities	M. Professional, scientific and	N. Administrative and support	O. Public administration and defence;	P. Education	Q. Human health and social work activities	R. Arts, entertainment and recreation	S. Other service activities	T. Activities of households as employers; undifferentiated goods- and services-producing activities
		A	B	C	D	E	F	G	H	I	J	K	L	M	N	O	P	Q	R	S	T
11	11 - Chief executives, senior officials and legislators	80%	80%	80%	80%	80%	80%	80%	80%	80%	80%	80%	80%	80%	80%	80%	80%	80%	80%	80%	80%
12	12 - Administrative and commercial managers	90%	90%	90%	90%	90%	90%	80%	90%	90%	90%	90%	90%	90%	90%	90%	90%	90%	80%	90%	90%
13	13 - Production and specialised services managers	60%	60%	60%	60%	60%	60%	80%	60%	60%	60%	60%	60%	60%	60%	60%	60%	60%	80%	60%	60%
14	14 - Hospitality, retail and other services managers	80%	80%	80%	80%	80%	80%	80%	80%	80%	80%	80%	80%	80%	80%	80%	80%	80%	80%	80%	80%
21	21 - Science and engineering professionals	80%	80%	80%	80%	80%	80%	80%	80%	80%	80%	80%	80%	80%	80%	80%	80%	80%	80%	80%	80%
22	22 - Health professionals	20%	20%	20%	20%	20%	20%	20%	20%	20%	20%	20%	20%	20%	20%	20%	20%	20%	20%	20%	20%
23	23 - Teaching professionals	80%	80%	80%	80%	80%	80%	80%	80%	80%	80%	80%	80%	80%	80%	80%	80%	80%	80%	80%	80%
24	24 - Business and administration professionals	80%	80%	80%	80%	80%	80%	80%	80%	80%	80%	80%	80%	80%	80%	80%	80%	80%	80%	80%	80%
25	25 - Information and communications technology professionals	80%	80%	80%	80%	80%	80%	80%	80%	80%	80%	80%	80%	80%	80%	80%	80%	80%	80%	80%	80%
26	26 - Legal, social and cultural professionals	70%	70%	70%	70%	70%	70%	80%	70%	70%	70%	70%	70%	70%	70%	70%	70%	70%	80%	70%	70%
31	31 - Science and engineering associate professionals	80%	80%	80%	80%	80%	80%	80%	80%	80%	80%	80%	80%	80%	80%	80%	80%	80%	80%	80%	80%
32	32 - Health associate professionals	20%	20%	20%	20%	20%	20%	20%	20%	20%	20%	20%	20%	20%	20%	20%	20%	20%	20%	20%	20%
33	33 - Business and administration associate professionals	80%	80%	80%	80%	80%	80%	80%	80%	80%	80%	80%	80%	80%	80%	80%	80%	80%	80%	80%	80%
34	34 - Legal, social, cultural and related associate professionals	70%	70%	70%	70%	70%	70%	80%	70%	70%	70%	70%	70%	70%	70%	70%	70%	70%	80%	70%	70%
35	35 - Information and communications technicians	80%	80%	80%	80%	80%	80%	80%	80%	80%	80%	80%	80%	80%	80%	80%	80%	80%	80%	80%	80%
41	41 - General and keyboard clerks	80%	80%	80%	80%	80%	80%	30%	80%	80%	80%	80%	80%	80%	80%	80%	80%	80%	30%	80%	80%
42	42 - Customer services clerks	80%	80%	80%	80%	80%	80%	30%	80%	80%	80%	80%	80%	80%	80%	80%	80%	80%	30%	80%	80%
43	43 - Numerical and material recording clerks	80%	80%	80%	80%	80%	80%	60%	80%	80%	80%	80%	80%	80%	80%	80%	80%	80%	60%	80%	80%
44	44 - Other clerical support workers	80%	80%	80%	80%	80%	80%	60%	80%	80%	80%	80%	80%	80%	80%	80%	80%	80%	60%	80%	80%
51	51 - Personal service workers	40%	40%	40%	40%	40%	40%	20%	40%	40%	40%	40%	40%	40%	40%	40%	40%	40%	20%	40%	40%
52	52 - Sales workers	20%	20%	20%	20%	20%	20%	20%	20%	20%	20%	20%	20%	20%	20%	20%	20%	20%	20%	20%	20%
53	53 - Personal care workers	40%	40%	40%	40%	40%	40%	0%	40%	40%	40%	40%	40%	40%	40%	40%	40%	40%	0%	40%	40%
54	54 - Protective services workers	40%	40%	40%	40%	40%	40%	30%	40%	40%	40%	40%	40%	40%	40%	40%	40%	40%	30%	40%	40%
61	61 - Market-oriented skilled agricultural workers	10%	10%	10%	10%	10%	10%	0%	10%	10%	10%	10%	10%	10%	10%	10%	10%	10%	0%	10%	10%
62	62 - Market-oriented skilled forestry, fishery and hunting workers	10%	10%	10%	10%	10%	10%	0%	10%	10%	10%	10%	10%	10%	10%	10%	10%	10%	0%	10%	10%
63	63 - Subsistence farmers, fishers, hunters and gatherers	0%	0%	0%	0%	0%	0%	0%	0%	0%	0%	0%	0%	0%	0%	0%	0%	0%	0%	0%	0%
71	71 - Building and related trades workers, excluding electricians	0%	0%	0%	0%	0%	0%	50%	0%	0%	0%	0%	0%	0%	0%	0%	0%	0%	50%	0%	0%
72	72 - Metal, machinery and related trades workers	10%	10%	10%	10%	10%	10%	30%	10%	10%	10%	10%	10%	10%	10%	10%	10%	10%	30%	10%	10%
73	73 - Handicraft and printing workers	0%	0%	0%	0%	0%	0%	20%	0%	0%	0%	0%	0%	0%	0%	0%	0%	0%	20%	0%	0%
74	74 - Electrical and electronic trades workers	10%	10%	10%	10%	10%	10%	60%	10%	10%	10%	10%	10%	10%	10%	10%	10%	10%	60%	10%	10%
75	75 - Food processing, wood working, garment and other craft and related trades workers	10%	10%	10%	10%	10%	10%	70%	10%	10%	10%	10%	10%	10%	10%	10%	10%	10%	70%	10%	10%
81	81 - Stationary plant and machine operators	0%	0%	0%	0%	0%	0%	50%	0%	0%	0%	0%	0%	0%	0%	0%	0%	0%	50%	0%	0%
82	82 - Assemblers	0%	0%	0%	0%	0%	0%	50%	0%	0%	0%	0%	0%	0%	0%	0%	0%	0%	50%	0%	0%
83	83 - Drivers and mobile plant operators	0%	0%	0%	0%	0%	0%	60%	0%	0%	0%	0%	0%	0%	0%	0%	0%	0%	60%	0%	0%
91	91 - Cleaners and helpers	0%	0%	0%	0%	0%	0%	35%	0%	0%	0%	0%	0%	0%	0%	0%	0%	0%	35%	0%	0%
92	92 - Agricultural, forestry and fishery labourers	10%	10%	10%	10%	10%	10%	10%	10%	10%	10%	10%	10%	10%	10%	10%	10%	10%	10%	10%	10%
93	93 - Labourers in mining, construction, manufacturing and transport	10%	10%	10%	10%	10%	10%	0%	10%	10%	10%	10%	10%	10%	10%	10%	10%	10%	0%	10%	10%
94	94 - Food preparation assistants	10%	10%	10%	10%	10%	10%	0%	10%	10%	10%	10%	10%	10%	10%	10%	10%	10%	0%	10%	10%
95	95 - Street and related sales and service workers	10%	10%	10%	10%	10%	10%	35%	10%	10%	10%	10%	10%	10%	10%	10%	10%	10%	35%	10%	10%
96	96 - Refuse workers and other elementary workers	10%	10%	10%	10%	10%	10%	35%	10%	10%	10%	10%	10%	10%	10%	10%	10%	10%	35%	10%	10%

United Kingdom

COVID reached the United Kingdom in late January, 2020. The government introduced a range of measures to mitigate the spread of COVID-19 including travel restrictions, social distancing measures, closures of entertainment, hospitality, non-essential shops and indoor premises, and increased testing. A national lockdown was introduced on 23 March. Essential workers included health and social care, education and childcare, key public services (including those essential to the running of the justice system, religious staff, charities and workers delivering key frontline services, and journalists and broadcasters who are providing public service broadcasting), local and national government, public safety and national security, transport, utilities, communication and financial services (including staff needed for essential financial services provision, the oil, gas, electricity and water sectors, information technology and data infrastructure sector and primary industry supplies to continue during the COVID-19 response, as well as key staff working in the civil nuclear, chemicals, telecommunications, postal services and delivery, payments providers and waste disposal sectors). The government have encouraged businesses and workplaces to encourage their employees to work at home, wherever possible.

Percent of jobs that were essential during lockdown

		A. Agriculture; forestry and fishing	B. Mining and quarrying	C. Manufacturi ng	D. Electricity; gas, steam and air	E. Water supply; sewerage, waste management and remediation	F. Constructio n	G. Wholesale and retail trade; repair of motor vehicles and	H. Transportati on and storage	I. Accommoda tion and food service	J. Information and communicat	K. Financial and insurance activities	L. Real estate activities	M. Professional , scientific and	N. Administrati ve and support	O. Public administrati on and defence;	P. Education	Q. Human health and social work activities	R. Arts, entertainme nt and recreation	S. Other service activities	T. Activities of households as employers; undifferentiated goods- and services-producing activities
**Isco\ Nace**	**Occupation description**	**A**	**B**	**C**	**D**	**E**	**F**	**G**	**H**	**I**	**J**	**K**	**L**	**M**	**N**	**O**	**P**	**Q**	**R**	**S**	**T**
**11**	11 - Chief executives, senior officials and legislators	100%	10%	10%	10%	10%	0%	0%	50%	0%	25%	25%	0%	0%	0%	100%	0%	100%	0%	0%	0%
**12**	12 - Administrative and commercial managers	100%	10%	10%	10%	10%	0%	0%	50%	0%	25%	25%	0%	0%	0%	100%	0%	100%	0%	0%	0%
**13**	13 - Production and specialised services managers	100%	10%	10%	10%	10%	0%	0%	50%	0%	25%	25%	0%	0%	0%	100%	0%	100%	0%	0%	0%
**14**	14 - Hospitality, retail and other services managers	100%	10%	10%	10%	10%	0%	0%	50%	0%	25%	25%	0%	0%	0%	100%	0%	100%	0%	0%	0%
**21**	21 - Science and engineering professionals	100%	10%	10%	10%	10%	0%	0%	50%	0%	25%	25%	0%	0%	0%	100%	0%	100%	0%	0%	0%
**22**	22 - Health professionals	100%	100%	100%	100%	100%	100%	100%	100%	100%	100%	100%	100%	100%	100%	100%	100%	100%	100%	100%	100%
**23**	23 - Teaching professionals	100%	20%	20%	20%	20%	20%	0%	20%	0%	20%	20%	20%	20%	20%	100%	20%	0%	0%	20%	20%
**24**	24 - Business and administration professionals	100%	10%	10%	10%	10%	0%	0%	50%	0%	25%	25%	0%	0%	0%	100%	0%	100%	0%	0%	0%
**25**	25 - Information and communications technology professionals	100%	10%	10%	10%	10%	0%	0%	0%	0%	25%	25%	0%	0%	0%	100%	0%	100%	0%	0%	0%
**26**	26 - Legal, social and cultural professionals	100%	0%	0%	0%	0%	0%	0%	0%	0%	0%	0%	0%	0%	0%	100%	0%	0%	0%	0%	0%
**31**	31 - Science and engineering associate professionals	100%	10%	10%	10%	10%	0%	0%	50%	0%	25%	25%	0%	0%	0%	100%	0%	100%	0%	0%	0%
**32**	32 - Health associate professionals	100%	100%	100%	100%	100%	100%	100%	100%	100%	100%	100%	100%	100%	100%	100%	100%	100%	100%	100%	100%
**33**	33 - Business and administration associate professionals	100%	10%	10%	10%	10%	0%	0%	50%	0%	25%	25%	0%	0%	0%	100%	0%	100%	0%	0%	0%
**34**	34 - Legal, social, cultural and related associate professionals	100%	0%	0%	0%	0%	0%	0%	0%	0%	0%	0%	0%	0%	0%	100%	0%	100%	0%	0%	0%
**35**	35 - Information and communications technicians	100%	10%	10%	10%	10%	0%	0%	50%	0%	25%	25%	0%	0%	0%	100%	0%	100%	0%	0%	0%
**41**	41 - General and keyboard clerks	100%	10%	10%	10%	10%	0%	0%	50%	0%	25%	25%	0%	0%	0%	100%	0%	100%	0%	0%	0%
**42**	42 - Customer services clerks	100%	10%	10%	10%	10%	0%	0%	0%	0%	25%	25%	0%	0%	0%	100%	0%	100%	0%	0%	0%
**43**	43 - Numerical and material recording clerks	100%	10%	10%	10%	10%	0%	0%	50%	0%	25%	25%	0%	0%	0%	100%	0%	100%	0%	0%	0%
**44**	44 - Other clerical support workers	100%	10%	10%	10%	10%	0%	0%	0%	0%	25%	25%	0%	0%	0%	100%	0%	100%	0%	0%	0%
**51**	51 - Personal service workers	100%	0%	0%	0%	0%	0%	0%	0%	0%	0%	0%	0%	0%	0%	100%	0%	0%	0%	0%	0%
**52**	52 - Sales workers	100%	0%	0%	0%	0%	0%	0%	0%	0%	0%	0%	0%	0%	0%	100%	0%	0%	0%	0%	0%
**53**	53 - Personal care workers	100%	10%	10%	10%	10%	0%	100%	0%	0%	25%	25%	0%	0%	0%	100%	0%	100%	0%	0%	0%
**54**	54 - Protective services workers	100%	10%	10%	10%	10%	0%	0%	0%	0%	25%	25%	0%	0%	0%	100%	0%	100%	0%	0%	0%
**61**	61 - Market-oriented skilled agricultural workers	100%	10%	10%	10%	10%	0%	0%	0%	0%	25%	25%	100%	100%	100%	100%	0%	0%	100%	100%	100%
**62**	62 - Market-oriented skilled forestry, fishery and hunting workers	100%	10%	10%	10%	10%	0%	0%	0%	0%	25%	25%	0%	0%	0%	100%	0%	0%	0%	0%	0%
**63**	63 - Subsistence farmers, fishers, hunters and gatherers	100%	100%	100%	100%	100%	100%	100%	100%	100%	100%	100%	100%	100%	100%	100%	100%	100%	100%	100%	100%
**71**	71 - Building and related trades workers, excluding electricians	100%	10%	10%	10%	10%	0%	0%	0%	0%	25%	25%	0%	0%	0%	100%	0%	0%	0%	0%	0%
**72**	72 - Metal, machinery and related trades workers	100%	10%	10%	10%	10%	0%	0%	0%	0%	25%	25%	0%	0%	0%	100%	0%	0%	0%	0%	0%
**73**	73 - Handicraft and printing workers	100%	10%	10%	10%	10%	0%	0%	0%	0%	25%	25%	0%	0%	0%	100%	0%	0%	0%	0%	0%
**74**	74 - Electrical and electronic trades workers	100%	10%	10%	10%	10%	0%	0%	0%	0%	25%	25%	0%	0%	0%	100%	0%	100%	0%	0%	0%
**75**	75 - Food processing, wood working, garment and other craft and related trades workers	100%	10%	10%	10%	10%	0%	0%	0%	25%	25%	25%	0%	0%	0%	100%	0%	0%	0%	0%	0%
**81**	81 - Stationary plant and machine operators	100%	10%	10%	10%	10%	0%	0%	0%	0%	25%	25%	0%	0%	0%	100%	0%	0%	0%	0%	0%
**82**	82 - Assemblers	100%	10%	10%	10%	10%	0%	0%	0%	0%	25%	25%	0%	0%	0%	100%	0%	0%	0%	0%	0%
**83**	83 - Drivers and mobile plant operators	100%	10%	10%	10%	10%	0%	0%	50%	25%	25%	25%	0%	0%	0%	100%	0%	50%	0%	0%	0%
**91**	91 - Cleaners and helpers	100%	10%	10%	10%	10%	0%	0%	0%	0%	25%	25%	0%	0%	0%	100%	0%	100%	0%	0%	0%
**92**	92 - Agricultural, forestry and fishery labourers	100%	10%	10%	10%	10%	0%	0%	50%	0%	25%	25%	0%	0%	0%	100%	0%	0%	0%	0%	0%
**93**	93 - Labourers in mining, construction, manufacturing and transport	100%	10%	10%	10%	10%	0%	0%	25%	0%	25%	25%	0%	0%	0%	100%	0%	0%	0%	0%	0%
**94**	94 - Food preparation assistants	100%	10%	10%	10%	10%	0%	100%	25%	25%	25%	25%	0%	0%	0%	100%	0%	100%	0%	0%	0%
**95**	95 - Street and related sales and service workers	100%	10%	10%	10%	10%	0%	0%	0%	0%	25%	25%	0%	0%	0%	100%	0%	100%	0%	0%	0%
**96**	96 - Refuse workers and other elementary workers	100%	10%	10%	10%	10%	0%	0%	0%	0%	25%	25%	0%	0%	0%	100%	0%	100%	0%	0%	0%

United Kingdom

Percent of jobs that were closed during lockdown

Isco\ Nace	Occupation description	A. Agriculture; forestry and fishing	B. Mining and quarrying	C. Manufacturing	D. Electricity; gas, steam and air	E. Water supply; sewerage, waste management and remediation	F. Construction	G. Wholesale and retail trade; repair of motor vehicles and	H. Transportation and storage	I. Accommodation and food service	J. Information and communicat	K. Financial and insurance activities	L. Real estate activities	M. Professional, scientific and	N. Administrative and support	O. Public administration and defence;	P. Education	Q. Human health and social work activities	R. Arts, entertainment and recreation	S. Other service activities	T. Activities of households as employers; undifferentiated goods- and services-producing activities
Isco\ Nace	Occupation description	A	B	C	D	E	F	G	H	I	J	K	L	M	N	O	P	Q	R	S	T
11	11 - Chief executives, senior officials and legislators	0%	0%	0%	0%	0%	0%	100%	0%	100%	0%	0%	0%	0%	0%	0%	0%	0%	100%	100%	100%
12	12 - Administrative and commercial managers	0%	0%	0%	0%	0%	0%	100%	0%	100%	0%	0%	0%	0%	0%	0%	0%	0%	100%	100%	100%
13	13 - Production and specialised services managers	0%	0%	0%	0%	0%	0%	100%	0%	100%	0%	0%	0%	0%	0%	0%	0%	0%	100%	100%	100%
14	14 - Hospitality, retail and other services managers	0%	0%	0%	0%	0%	0%	100%	0%	100%	0%	0%	0%	0%	0%	0%	0%	0%	100%	100%	100%
21	21 - Science and engineering professionals	0%	0%	0%	0%	0%	0%	100%	0%	100%	0%	0%	0%	0%	0%	0%	0%	0%	100%	100%	100%
22	22 - Health professionals	0%	0%	0%	0%	0%	0%	0%	0%	0%	0%	0%	0%	0%	0%	0%	0%	0%	0%	0%	0%
23	23 - Teaching professionals	0%	0%	0%	0%	0%	0%	100%	0%	100%	0%	0%	0%	0%	0%	0%	0%	0%	100%	0%	0%
24	24 - Business and administration professionals	0%	0%	0%	0%	0%	0%	100%	0%	100%	0%	0%	0%	0%	0%	0%	0%	0%	100%	100%	100%
25	25 - Information and communications technology professionals	0%	0%	0%	0%	0%	0%	100%	0%	100%	0%	0%	0%	0%	0%	0%	0%	0%	100%	100%	100%
26	26 - Legal, social and cultural professionals	0%	75%	75%	75%	75%	75%	75%	75%	100%	75%	75%	75%	75%	75%	0%	75%	0%	100%	100%	100%
31	31 - Science and engineering associate professionals	0%	0%	0%	0%	0%	0%	100%	0%	100%	0%	0%	0%	0%	0%	0%	0%	0%	100%	100%	100%
32	32 - Health associate professionals	0%	0%	0%	0%	0%	0%	0%	0%	0%	0%	0%	0%	0%	0%	0%	0%	0%	0%	0%	0%
33	33 - Business and administration associate professionals	0%	0%	0%	0%	0%	0%	100%	0%	100%	0%	0%	0%	0%	0%	0%	0%	0%	100%	100%	100%
34	34 - Legal, social, cultural and related associate professionals	0%	75%	75%	75%	75%	75%	75%	75%	100%	75%	75%	75%	75%	75%	0%	75%	0%	100%	100%	100%
35	35 - Information and communications technicians	0%	0%	0%	0%	0%	0%	100%	0%	100%	0%	0%	0%	0%	0%	0%	0%	0%	100%	100%	100%
41	41 - General and keyboard clerks	0%	0%	0%	0%	0%	0%	100%	0%	100%	0%	0%	0%	0%	0%	0%	0%	0%	100%	100%	100%
42	42 - Customer services clerks	0%	0%	0%	0%	0%	0%	100%	0%	100%	0%	0%	0%	0%	0%	0%	0%	0%	100%	100%	100%
43	43 - Numerical and material recording clerks	0%	0%	0%	0%	0%	0%	100%	0%	100%	0%	0%	0%	0%	0%	0%	0%	0%	100%	100%	100%
44	44 - Other clerical support workers	0%	0%	0%	0%	0%	0%	100%	0%	100%	0%	0%	0%	0%	0%	0%	0%	0%	100%	100%	100%
51	51 - Personal service workers	0%	100%	100%	100%	100%	100%	100%	100%	100%	100%	100%	100%	100%	100%	0%	100%	0%	100%	100%	100%
52	52 - Sales workers	0%	75%	75%	75%	75%	75%	75%	75%	0%	0%	0%	100%	100%	100%	0%	75%	0%	100%	100%	100%
53	53 - Personal care workers	0%	0%	0%	0%	0%	0%	0%	0%	100%	0%	0%	0%	0%	0%	0%	0%	0%	100%	100%	100%
54	54 - Protective services workers	0%	0%	0%	0%	0%	0%	100%	0%	100%	0%	0%	0%	0%	0%	0%	0%	0%	100%	100%	100%
61	61 - Market-oriented skilled agricultural workers	0%	0%	0%	0%	0%	0%	100%	0%	100%	0%	0%	0%	0%	0%	0%	0%	0%	0%	0%	0%
62	62 - Market-oriented skilled forestry, fishery and hunting workers	0%	0%	0%	0%	0%	0%	100%	0%	100%	0%	0%	0%	0%	0%	0%	0%	0%	100%	100%	100%
63	63 - Subsistence farmers, fishers, hunters and gatherers	0%	0%	0%	0%	0%	0%	0%	0%	0%	0%	0%	0%	0%	0%	0%	0%	0%	0%	0%	0%
71	71 - Building and related trades workers, excluding electricians	0%	0%	0%	0%	0%	0%	100%	0%	100%	0%	0%	0%	0%	0%	0%	0%	0%	100%	100%	100%
72	72 - Metal, machinery and related trades workers	0%	0%	0%	0%	0%	0%	100%	0%	100%	0%	0%	0%	0%	0%	0%	0%	0%	100%	100%	100%
73	73 - Handicraft and printing workers	0%	0%	0%	0%	0%	0%	100%	0%	100%	0%	0%	0%	0%	0%	0%	0%	0%	100%	100%	100%
74	74 - Electrical and electronic trades workers	0%	0%	0%	0%	0%	0%	100%	0%	100%	0%	0%	0%	0%	0%	0%	0%	0%	100%	100%	100%
75	75 - Food processing, wood working, garment and other craft and related trades workers	0%	0%	0%	0%	0%	0%	100%	0%	0%	0%	0%	0%	0%	0%	0%	100%	0%	100%	100%	100%
81	81 - Stationary plant and machine operators	0%	0%	0%	0%	0%	0%	100%	0%	100%	0%	0%	0%	0%	0%	0%	0%	0%	100%	100%	100%
82	82 - Assemblers	0%	0%	0%	0%	0%	0%	100%	0%	100%	0%	0%	0%	0%	0%	0%	0%	0%	100%	100%	100%
83	83 - Drivers and mobile plant operators	0%	0%	0%	0%	0%	0%	100%	0%	0%	0%	0%	0%	0%	0%	0%	0%	0%	100%	100%	100%
91	91 - Cleaners and helpers	0%	0%	0%	0%	0%	0%	100%	0%	100%	0%	0%	0%	0%	0%	0%	100%	0%	100%	100%	100%
92	92 - Agricultural, forestry and fishery labourers	0%	0%	0%	0%	0%	0%	100%	0%	100%	0%	0%	0%	0%	0%	0%	0%	0%	100%	100%	100%
93	93 - Labourers in mining, construction, manufacturing and transport	0%	0%	0%	0%	0%	0%	100%	0%	100%	0%	0%	0%	0%	0%	0%	0%	0%	100%	100%	100%
94	94 - Food preparation assistants	0%	0%	0%	0%	0%	0%	0%	0%	0%	0%	0%	0%	0%	0%	0%	0%	0%	100%	100%	100%
95	95 - Street and related sales and service workers	0%	0%	0%	0%	0%	0%	100%	0%	100%	0%	0%	0%	0%	0%	0%	0%	0%	100%	100%	100%
96	96 - Refuse workers and other elementary workers	0%	0%	0%	0%	0%	0%	100%	0%	100%	0%	0%	0%	0%	0%	0%	0%	0%	100%	100%	100%

United Kingdom

Percent of jobs subject to teleworking during lockdown

Isco\ Nace	Occupation description	A. Agriculture; forestry and fishing	B. Mining and quarrying	C. Manufacturing	D. Electricity; gas, steam and air	E. Water supply; sewerage, waste management and remediation	F. Construction	G. Wholesale and retail trade; repair of motor vehicles and	H. Transportation and storage	I. Accommodation and food service	J. Information and communicat	K. Financial and insurance activities	L. Real estate activities	M. Professional, scientific and	N. Administrative and support	O. Public administration and defence;	P. Education	Q. Human health and social work activities	R. Arts, entertainment and recreation	S. Other service activities	T. Activities of households as employers; undifferentiated goods- and services-producing activities
		A	B	C	D	E	F	G	H	I	J	K	L	M	N	O	P	Q	R	S	T
11	11 - Chief executives, senior officials and legislators	0%	90%	90%	90%	90%	100%	0%	50%	0%	75%	75%	100%	100%	100%	0%	100%	0%	0%	0%	0%
12	12 - Administrative and commercial managers	0%	90%	90%	90%	90%	100%	0%	50%	0%	75%	75%	100%	100%	100%	0%	100%	0%	0%	0%	0%
13	13 - Production and specialised services managers	0%	90%	90%	90%	90%	100%	0%	50%	0%	75%	75%	100%	100%	100%	0%	100%	0%	0%	0%	0%
14	14 - Hospitality, retail and other services managers	0%	90%	90%	90%	90%	100%	0%	50%	0%	75%	75%	100%	100%	100%	0%	100%	0%	0%	0%	0%
21	21 - Science and engineering professionals	0%	90%	90%	90%	90%	100%	0%	50%	0%	75%	75%	100%	100%	100%	0%	100%	0%	0%	0%	0%
22	22 - Health professionals	0%	0%	0%	0%	0%	0%	0%	0%	0%	0%	0%	0%	0%	0%	0%	0%	0%	0%	0%	0%
23	23 - Teaching professionals	0%	80%	80%	80%	80%	80%	0%	80%	0%	80%	80%	80%	80%	80%	0%	80%	100%	0%	80%	80%
24	24 - Business and administration professionals	0%	90%	90%	90%	90%	100%	0%	50%	0%	75%	75%	100%	100%	100%	0%	100%	0%	0%	0%	0%
25	25 - Information and communications technology professionals	0%	90%	90%	90%	90%	100%	0%	100%	0%	75%	75%	100%	100%	100%	0%	100%	0%	0%	0%	0%
26	26 - Legal, social and cultural professionals	0%	25%	25%	25%	25%	25%	25%	25%	0%	25%	25%	25%	25%	25%	0%	25%	100%	0%	0%	0%
31	31 - Science and engineering associate professionals	0%	90%	90%	90%	90%	100%	0%	50%	0%	75%	75%	100%	100%	100%	0%	100%	0%	0%	0%	0%
32	32 - Health associate professionals	0%	0%	0%	0%	0%	0%	0%	0%	0%	0%	0%	0%	0%	0%	0%	0%	0%	0%	0%	0%
33	33 - Business and administration associate professionals	0%	90%	90%	90%	90%	100%	0%	50%	0%	75%	75%	100%	100%	100%	0%	100%	0%	0%	0%	0%
34	34 - Legal, social, cultural and related associate professionals	0%	25%	25%	25%	25%	25%	25%	25%	0%	25%	25%	25%	25%	25%	0%	25%	0%	0%	0%	0%
35	35 - Information and communications technicians	0%	90%	90%	90%	90%	100%	0%	50%	0%	75%	75%	100%	100%	100%	0%	100%	0%	0%	0%	0%
41	41 - General and keyboard clerks	0%	90%	90%	90%	90%	100%	0%	50%	0%	75%	75%	100%	100%	100%	0%	100%	0%	0%	0%	0%
42	42 - Customer services clerks	0%	90%	90%	90%	90%	100%	0%	100%	0%	75%	75%	100%	100%	100%	0%	100%	0%	0%	0%	0%
43	43 - Numerical and material recording clerks	0%	90%	90%	90%	90%	100%	0%	50%	0%	75%	75%	100%	100%	100%	0%	100%	0%	0%	0%	0%
44	44 - Other clerical support workers	0%	90%	90%	90%	90%	100%	0%	100%	0%	75%	75%	100%	100%	100%	0%	100%	0%	0%	0%	0%
51	51 - Personal service workers	0%	0%	0%	0%	0%	0%	0%	0%	0%	0%	0%	0%	0%	0%	0%	0%	100%	0%	0%	0%
52	52 - Sales workers	0%	25%	25%	25%	25%	25%	25%	25%	100%	100%	100%	0%	0%	0%	0%	25%	100%	0%	0%	0%
53	53 - Personal care workers	0%	90%	90%	90%	90%	100%	0%	100%	0%	75%	75%	100%	100%	100%	0%	100%	0%	0%	0%	0%
54	54 - Protective services workers	0%	90%	90%	90%	90%	100%	0%	100%	0%	75%	75%	100%	100%	100%	0%	100%	0%	0%	0%	0%
61	61 - Market-oriented skilled agricultural workers	0%	90%	90%	90%	90%	100%	0%	100%	0%	75%	75%	0%	0%	0%	0%	100%	100%	0%	0%	0%
62	62 - Market-oriented skilled forestry, fishery and hunting workers	0%	90%	90%	90%	90%	100%	0%	100%	0%	75%	75%	100%	100%	100%	0%	100%	100%	0%	0%	0%
63	63 - Subsistence farmers, fishers, hunters and gatherers	0%	0%	0%	0%	0%	0%	0%	0%	0%	0%	0%	0%	0%	0%	0%	0%	0%	0%	0%	0%
71	71 - Building and related trades workers, excluding electricians	0%	90%	90%	90%	90%	100%	0%	100%	0%	75%	75%	100%	100%	100%	0%	100%	100%	0%	0%	0%
72	72 - Metal, machinery and related trades workers	0%	90%	90%	90%	90%	100%	0%	100%	0%	75%	75%	100%	100%	100%	0%	100%	100%	0%	0%	0%
73	73 - Handicraft and printing workers	0%	90%	90%	90%	90%	100%	0%	100%	0%	75%	75%	100%	100%	100%	0%	100%	100%	0%	0%	0%
74	74 - Electrical and electronic trades workers	0%	90%	90%	90%	90%	100%	0%	100%	0%	75%	75%	100%	100%	100%	0%	100%	0%	0%	0%	0%
75	75 - Food processing, wood working, garment and other craft and related trades workers	0%	90%	90%	90%	90%	100%	0%	100%	75%	75%	75%	100%	100%	100%	0%	0%	100%	0%	0%	0%
81	81 - Stationary plant and machine operators	0%	90%	90%	90%	90%	100%	0%	100%	0%	75%	75%	100%	100%	100%	0%	100%	100%	0%	0%	0%
82	82 - Assemblers	0%	90%	90%	90%	90%	100%	0%	100%	0%	75%	75%	100%	100%	100%	0%	100%	100%	0%	0%	0%
83	83 - Drivers and mobile plant operators	0%	90%	90%	90%	90%	100%	0%	50%	75%	75%	75%	100%	100%	100%	0%	100%	50%	0%	0%	0%
91	91 - Cleaners and helpers	0%	90%	90%	90%	90%	100%	0%	100%	0%	75%	75%	100%	100%	100%	0%	0%	0%	0%	0%	0%
92	92 - Agricultural, forestry and fishery labourers	0%	90%	90%	90%	90%	100%	0%	50%	0%	75%	75%	100%	100%	100%	0%	100%	100%	0%	0%	0%
93	93 - Labourers in mining, construction, manufacturing and transport	0%	90%	90%	90%	90%	100%	0%	75%	0%	75%	75%	100%	100%	100%	0%	100%	100%	0%	0%	0%
94	94 - Food preparation assistants	0%	90%	90%	90%	90%	100%	0%	75%	75%	75%	75%	100%	100%	100%	0%	100%	0%	0%	0%	0%
95	95 - Street and related sales and service workers	0%	90%	90%	90%	90%	100%	0%	100%	0%	75%	75%	100%	100%	100%	0%	100%	0%	0%	0%	0%
96	96 - Refuse workers and other elementary workers	0%	90%	90%	90%	90%	100%	0%	100%	0%	75%	75%	100%	100%	100%	0%	100%	0%	0%	0%	0%

Georgia

Georgia has registered the first case of Covid-19 on 28 February 2020. However, the country started implementation of national response measures as early as in January, even before the WHO has declared it as a pandemic. Regulations started with mandatory thermo screening at the airports and suspension of flights from high-affected areas, leading to cancelation of all air travel on March 14-16 and follow up cancelation of public events, schools did not open after winter holidays and employers were urged to switch to home-based working mode, wherever possible.

Georgia declared a state of public emergency on March 21, which included cancelation of public transportation, curfews and lockdown for up to two months. Jobs included under essential list included healthcare, agriculture, critical infrastructure (roads, construction, water and sewage systems), wholesale, storage and transportation, police, defense and rescue services. The hospitality sector continued operation and they served as quarantine zones (on average, 5 000 individuals were quarantined) . Jobs that were restricted included street sale, art and entertainment , retail service other than food and pharmacy products (online sales were also restricted for small period of time) . All schools, kindergartens and universities were encouraged to switch to teleworking. All conferences, seminars and similar events were similarly encouraged to telework, as were most of the public servant positions and the financial industry (banks, insurance).

On May 22, Georgia started gradual relaxation of strict measures.

Percent of jobs that were essential during lockdown

		A. Agriculture; forestry and fishing	B. Mining and quarrying	C. Manufacturing	D. Electricity; gas, steam and air	E. Water supply; sewerage, waste management and remediation	F. Construction	G. Wholesale and retail trade; repair of motor vehicles and	H. Transportation and storage	I. Accommodation and food service	J. Information and communicat	K. Financial and insurance activities	L. Real estate activities	M. Professional, scientific and	N. Administrative and support	O. Public administration and defence;	P. Education	Q. Human health and social work activities	R. Arts, entertainment and recreation	S. Other service activities	T. Activities of households as employers; undifferentiated goods- and services-producing activities
**Isco\ Nace**	**Occupation description**	**A**	**B**	**C**	**D**	**E**	**F**	**G**	**H**	**I**	**J**	**K**	**L**	**M**	**N**	**O**	**P**	**Q**	**R**	**S**	**T**
**11**	11 - Chief executives, senior officials and legislators	100%	10%	10%	10%	100%	100%	50%	50%	10%	25%	25%	0%	0%	0%	100%	0%	100%	0%	0%	0%
**12**	12 - Administrative and commercial managers	100%	10%	10%	10%	100%	100%	50%	50%	10%	25%	25%	0%	0%	0%	100%	0%	100%	0%	0%	0%
**13**	13 - Production and specialised services managers	100%	10%	10%	10%	100%	100%	50%	50%	10%	25%	25%	0%	0%	0%	100%	0%	100%	0%	0%	0%
**14**	14 - Hospitality, retail and other services managers	100%	10%	10%	10%	100%	100%	50%	50%	10%	25%	25%	0%	0%	0%	100%	0%	100%	0%	0%	0%
**21**	21 - Science and engineering professionals	100%	10%	10%	10%	100%	100%	50%	50%	10%	25%	25%	0%	0%	0%	100%	0%	100%	0%	0%	0%
**22**	22 - Health professionals	100%	100%	100%	100%	100%	100%	100%	100%	100%	100%	100%	100%	100%	100%	100%	100%	100%	0%	100%	100%
**23**	23 - Teaching professionals	10%	0%	10%	10%	10%	100%	50%	0%	10%	25%	25%	0%	0%	0%	100%	0%	0%	0%	0%	0%
**24**	24 - Business and administration professionals	100%	10%	10%	10%	100%	100%	50%	50%	10%	25%	25%	0%	0%	0%	100%	0%	100%	0%	0%	0%
**25**	25 - Information and communications technology professionals	100%	10%	10%	10%	100%	100%	50%	0%	10%	25%	25%	0%	0%	0%	100%	0%	100%	0%	0%	0%
**26**	26 - Legal, social and cultural professionals	100%	0%	0%	0%	100%	100%	50%	0%	10%	0%	0%	0%	0%	0%	100%	0%	0%	0%	0%	0%
**31**	31 - Science and engineering associate professionals	100%	10%	10%	10%	100%	100%	50%	50%	10%	25%	25%	0%	0%	0%	100%	0%	100%	0%	0%	0%
**32**	32 - Health associate professionals	100%	100%	100%	100%	100%	100%	100%	100%	100%	100%	100%	100%	100%	100%	100%	100%	100%	100%	100%	100%
**33**	33 - Business and administration associate professionals	100%	10%	10%	10%	100%	100%	50%	50%	10%	25%	25%	0%	0%	0%	100%	0%	100%	0%	0%	0%
**34**	34 - Legal, social, cultural and related associate professionals	100%	0%	0%	0%	100%	100%	50%	0%	10%	0%	0%	0%	0%	0%	100%	0%	100%	0%	0%	0%
**35**	35 - Information and communications technicians	100%	10%	10%	10%	100%	100%	50%	50%	10%	25%	25%	0%	0%	0%	100%	0%	100%	0%	0%	0%
**41**	41 - General and keyboard clerks	100%	10%	10%	10%	100%	100%	50%	50%	10%	25%	25%	0%	0%	0%	100%	0%	100%	0%	0%	0%
**42**	42 - Customer services clerks	100%	10%	10%	10%	100%	100%	50%	0%	10%	25%	25%	0%	0%	0%	100%	0%	100%	0%	0%	0%
**43**	43 - Numerical and material recording clerks	100%	10%	10%	10%	100%	100%	50%	50%	10%	25%	25%	0%	0%	0%	100%	0%	100%	0%	0%	0%
**44**	44 - Other clerical support workers	100%	10%	10%	10%	10%	100%	50%	0%	10%	25%	25%	0%	0%	0%	100%	0%	100%	0%	0%	0%
**51**	51 - Personal service workers	100%	0%	0%	0%	0%	100%	50%	0%	10%	0%	0%	0%	0%	0%	100%	0%	0%	0%	0%	0%
**52**	52 - Sales workers	50%	0%	0%	0%	0%	100%	50%	0%	10%	0%	50%	0%	0%	0%	100%	0%	0%	0%	0%	0%
**53**	53 - Personal care workers	100%	10%	10%	10%	10%	100%	100%	0%	10%	25%	25%	0%	0%	0%	100%	0%	100%	0%	0%	0%
**54**	54 - Protective services workers	100%	10%	10%	10%	10%	100%	50%	0%	10%	25%	25%	0%	0%	0%	100%	0%	100%	0%	0%	0%
**61**	61 - Market-oriented skilled agricultural workers	100%	10%	10%	10%	10%	100%	50%	0%	10%	25%	25%	100%	100%	100%	100%	0%	0%	100%	100%	100%
**62**	62 - Market-oriented skilled forestry, fishery and hunting workers	100%	10%	10%	10%	10%	100%	50%	0%	10%	25%	25%	0%	0%	0%	100%	0%	0%	0%	0%	0%
**63**	63 - Subsistence farmers, fishers, hunters and gatherers	100%	100%	100%	100%	100%	100%	100%	100%	100%	100%	100%	100%	100%	100%	100%	100%	100%	100%	100%	100%
**71**	71 - Building and related trades workers, excluding electricians	100%	10%	10%	10%	10%	100%	50%	0%	10%	25%	25%	0%	0%	0%	100%	0%	0%	0%	0%	0%
**72**	72 - Metal, machinery and related trades workers	100%	10%	10%	10%	10%	100%	50%	0%	10%	25%	25%	0%	0%	0%	100%	0%	0%	0%	0%	0%
**73**	73 - Handicraft and printing workers	100%	10%	10%	10%	10%	100%	50%	0%	10%	25%	25%	0%	0%	0%	100%	0%	0%	0%	0%	0%
**74**	74 - Electrical and electronic trades workers	100%	10%	10%	10%	10%	100%	50%	0%	10%	25%	25%	0%	0%	0%	100%	0%	100%	0%	0%	0%
**75**	75 - Food processing, wood working, garment and other craft and related trades workers	100%	10%	10%	10%	10%	100%	50%	0%	25%	25%	25%	0%	0%	0%	100%	0%	0%	0%	0%	0%
**81**	81 - Stationary plant and machine operators	100%	10%	10%	10%	10%	100%	50%	0%	10%	25%	25%	0%	0%	0%	100%	0%	0%	0%	0%	0%
**82**	82 - Assemblers	100%	10%	10%	10%	10%	100%	50%	0%	10%	25%	25%	0%	0%	0%	100%	0%	0%	0%	0%	0%
**83**	83 - Drivers and mobile plant operators	90%	10%	10%	10%	10%	100%	50%	50%	25%	25%	25%	0%	0%	0%	100%	0%	50%	0%	0%	0%
**91**	91 - Cleaners and helpers	90%	10%	10%	10%	10%	100%	50%	0%	10%	25%	25%	0%	0%	0%	100%	0%	100%	0%	0%	0%
**92**	92 - Agricultural, forestry and fishery labourers	90%	10%	10%	10%	10%	100%	50%	50%	10%	25%	25%	0%	0%	0%	100%	0%	0%	0%	0%	0%
**93**	93 - Labourers in mining, construction, manufacturing and transport	100%	10%	10%	10%	10%	100%	50%	25%	10%	25%	25%	0%	0%	0%	100%	0%	0%	0%	0%	0%
**94**	94 - Food preparation assistants	100%	10%	10%	10%	10%	100%	100%	25%	25%	25%	25%	0%	0%	0%	100%	0%	100%	0%	0%	0%
**95**	95 - Street and related sales and service workers	0%	0%	0%	0%	0%	0%	0%	0%	0%	0%	0%	0%	0%	0%	0%	0%	0%	0%	0%	0%
**96**	96 - Refuse workers and other elementary workers	100%	10%	10%	10%	10%	100%	50%	0%	0%	25%	25%	0%	0%	0%	100%	0%	100%	0%	0%	0%

Georgia

Percent of jobs that were closed during lockdown

		A. Agriculture; forestry and fishing	B. Mining and quarrying	C. Manufacturing	D. Electricity; gas, steam and air	E. Water supply; sewerage, waste management and remediation	F. Construction	G. Wholesale and retail trade; repair of motor vehicles and	H. Transportation and storage	I. Accommodation and food service	J. Information and communicat	K. Financial and insurance activities	L. Real estate activities	M. Professional, scientific and	N. Administrative and support	O. Public administration and defence;	P. Education	Q. Human health and social work activities	R. Arts, entertainment and recreation	S. Other service activities	T. Activities of households as employers; undifferentiated goods- and services-producing activities
																		0	1		
**Isco\ Nace**	**Occupation description**	**A**	**B**	**C**	**D**	**E**	**F**	**G**	**H**	**I**	**J**	**K**	**L**	**M**	**N**	**O**	**P**	**Q**	**R**	**S**	**T**
**11**	11 - Chief executives, senior officials and legislators	0%	0%	0%	0%	0%	0%	50%	0%	90%	0%	0%	0%	0%	0%	0%	0%	0%	100%	100%	100%
**12**	12 - Administrative and commercial managers	0%	0%	0%	0%	0%	0%	50%	0%	90%	0%	0%	0%	0%	0%	0%	0%	0%	100%	100%	100%
**13**	13 - Production and specialised services managers	0%	0%	0%	0%	0%	0%	50%	0%	90%	0%	0%	0%	0%	0%	0%	0%	0%	100%	100%	100%
**14**	14 - Hospitality, retail and other services managers	0%	0%	0%	0%	0%	0%	50%	0%	90%	0%	0%	0%	0%	0%	0%	0%	0%	100%	100%	100%
**21**	21 - Science and engineering professionals	0%	0%	0%	0%	0%	0%	50%	0%	90%	0%	0%	0%	0%	0%	0%	0%	0%	100%	100%	100%
**22**	22 - Health professionals	0%	0%	0%	0%	0%	0%	0%	0%	0%	0%	0%	0%	0%	0%	0%	0%	0%	0%	0%	0%
**23**	23 - Teaching professionals	0%	0%	0%	0%	0%	0%	50%	0%	90%	0%	0%	0%	0%	0%	0%	0%	0%	100%	100%	100%
**24**	24 - Business and administration professionals	0%	0%	0%	0%	0%	0%	50%	0%	90%	0%	0%	0%	0%	0%	0%	0%	0%	100%	100%	100%
**25**	25 - Information and communications technology professionals	0%	0%	0%	0%	0%	0%	50%	0%	90%	0%	0%	0%	0%	0%	0%	0%	0%	100%	100%	100%
**26**	26 - Legal, social and cultural professionals	0%	75%	75%	75%	0%	0%	50%	75%	90%	75%	75%	75%	75%	75%	0%	75%	0%	100%	100%	100%
**31**	31 - Science and engineering associate professionals	0%	0%	0%	0%	0%	0%	50%	0%	90%	0%	0%	0%	0%	0%	0%	0%	0%	100%	100%	100%
**32**	32 - Health associate professionals	0%	0%	0%	0%	0%	0%	0%	0%	0%	0%	0%	0%	0%	0%	0%	0%	0%	0%	0%	0%
**33**	33 - Business and administration associate professionals	0%	0%	0%	0%	0%	0%	50%	0%	90%	0%	0%	0%	0%	0%	0%	0%	0%	100%	100%	100%
**34**	34 - Legal, social, cultural and related associate professionals	0%	75%	75%	75%	0%	0%	50%	75%	90%	75%	75%	75%	75%	75%	0%	75%	0%	100%	100%	100%
**35**	35 - Information and communications technicians	0%	0%	0%	0%	0%	0%	50%	0%	90%	0%	0%	0%	0%	0%	0%	0%	0%	100%	100%	100%
**41**	41 - General and keyboard clerks	0%	0%	0%	0%	0%	0%	50%	0%	90%	0%	0%	0%	0%	0%	0%	0%	0%	100%	100%	100%
**42**	42 - Customer services clerks	0%	0%	0%	0%	0%	0%	50%	0%	90%	0%	0%	0%	0%	0%	0%	0%	0%	100%	100%	100%
**43**	43 - Numerical and material recording clerks	0%	0%	0%	0%	0%	0%	50%	0%	90%	0%	0%	0%	0%	0%	0%	0%	0%	100%	100%	100%
**44**	44 - Other clerical support workers	0%	0%	0%	0%	0%	0%	50%	0%	90%	0%	0%	0%	0%	0%	0%	0%	0%	100%	100%	100%
**51**	51 - Personal service workers	0%	100%	100%	100%	100%	0%	50%	100%	90%	100%	100%	100%	100%	100%	0%	100%	0%	100%	100%	100%
**52**	52 - Sales workers	0%	75%	75%	75%	75%	0%	50%	75%	90%	0%	0%	100%	100%	100%	0%	75%	0%	100%	100%	100%
**53**	53 - Personal care workers	0%	0%	0%	0%	0%	0%	0%	0%	90%	0%	0%	0%	0%	0%	0%	0%	0%	100%	100%	100%
**54**	54 - Protective services workers	0%	0%	0%	0%	0%	0%	50%	0%	90%	0%	0%	0%	0%	0%	0%	0%	0%	100%	100%	100%
**61**	61 - Market-oriented skilled agricultural workers	0%	0%	0%	0%	0%	0%	50%	0%	90%	0%	0%	0%	0%	0%	0%	0%	0%	0%	0%	0%
**62**	62 - Market-oriented skilled forestry, fishery and hunting workers	0%	0%	0%	0%	0%	0%	50%	0%	90%	0%	0%	0%	0%	0%	0%	0%	0%	100%	100%	100%
**63**	63 - Subsistence farmers, fishers, hunters and gatherers	0%	0%	0%	0%	0%	0%	0%	0%	0%	0%	0%	0%	0%	0%	0%	0%	0%	0%	0%	0%
**71**	71 - Building and related trades workers, excluding electricians	0%	0%	0%	0%	0%	0%	50%	0%	90%	0%	0%	0%	0%	0%	0%	0%	0%	100%	100%	100%
**72**	72 - Metal, machinery and related trades workers	0%	0%	0%	0%	0%	0%	50%	0%	90%	0%	0%	0%	0%	0%	0%	0%	0%	100%	100%	100%
**73**	73 - Handicraft and printing workers	0%	0%	0%	0%	0%	0%	50%	0%	90%	0%	0%	0%	0%	0%	0%	0%	0%	100%	100%	100%
**74**	74 - Electrical and electronic trades workers	0%	0%	0%	0%	0%	0%	50%	0%	90%	0%	0%	0%	0%	0%	0%	0%	0%	100%	100%	100%
**75**	75 - Food processing, wood working, garment and other craft and related trades workers	0%	0%	0%	0%	0%	0%	50%	0%	0%	0%	0%	0%	0%	0%	0%	100%	0%	100%	100%	100%
**81**	81 - Stationary plant and machine operators	0%	0%	0%	0%	0%	0%	50%	0%	90%	0%	0%	0%	0%	0%	0%	0%	0%	100%	100%	100%
**82**	82 - Assemblers	0%	0%	0%	0%	0%	0%	50%	0%	90%	0%	0%	0%	0%	0%	0%	0%	0%	100%	100%	100%
**83**	83 - Drivers and mobile plant operators	0%	0%	0%	0%	0%	0%	50%	0%	0%	0%	0%	0%	0%	0%	0%	0%	0%	100%	100%	100%
**91**	91 - Cleaners and helpers	0%	0%	0%	0%	0%	0%	50%	0%	90%	0%	0%	0%	0%	0%	0%	100%	0%	100%	100%	100%
**92**	92 - Agricultural, forestry and fishery labourers	0%	0%	0%	0%	0%	0%	50%	0%	90%	0%	0%	0%	0%	0%	0%	0%	0%	100%	100%	100%
**93**	93 - Labourers in mining, construction, manufacturing and transport	0%	0%	0%	0%	0%	0%	50%	0%	90%	0%	0%	0%	0%	0%	0%	0%	0%	100%	100%	100%
**94**	94 - Food preparation assistants	0%	0%	0%	0%	0%	0%	0%	0%	0%	0%	0%	0%	0%	0%	0%	0%	0%	100%	100%	100%
**95**	95 - Street and related sales and service workers	100%	100%	100%	100%	100%	100%	100%	100%	100%	100%	100%	100%	100%	100%	100%	100%	100%	100%	100%	100%
**96**	96 - Refuse workers and other elementary workers	0%	0%	0%	0%	0%	0%	0%	0%	100%	0%	0%	0%	0%	0%	0%	0%	0%	100%	100%	100%

Georgia

Percent of jobs subject to teleworking during lockdown

0 1

Isco\ Nace	Occupation description	A. Agriculture; forestry and fishing	B. Mining and quarrying	C. Manufacturing	D. Electricity; gas, steam and air	E. Water supply; sewerage, waste management and remediation	F. Construction	G. Wholesale and retail trade; repair of motor vehicles and	H. Transportation and storage	I. Accommodation and food service	J. Information and communicat	K. Financial and insurance activities	L. Real estate activities	M. Professional, scientific and	N. Administrative and support	O. Public administration and defence;	P. Education	Q. Human health and social work activities	R. Arts, entertainment and recreation	S. Other service activities	T. Activities of households as employers; undifferentiated goods- and services-producing activities
		A	B	C	D	E	F	G	H	I	J	K	L	M	N	O	P	Q	R	S	T
11	11 - Chief executives, senior officials and legislators	0%	90%	90%	90%	0%	0%	0%	50%	0%	75%	75%	100%	100%	100%	0%	100%	0%	0%	0%	0%
12	12 - Administrative and commercial managers	0%	90%	90%	90%	0%	0%	0%	50%	0%	75%	75%	100%	100%	100%	0%	100%	0%	0%	0%	0%
13	13 - Production and specialised services managers	0%	90%	90%	90%	0%	0%	0%	50%	0%	75%	75%	100%	100%	100%	0%	100%	0%	0%	0%	0%
14	14 - Hospitality, retail and other services managers	0%	90%	90%	90%	0%	0%	0%	50%	0%	75%	75%	100%	100%	100%	0%	100%	0%	0%	0%	0%
21	21 - Science and engineering professionals	0%	90%	90%	90%	0%	0%	0%	50%	0%	75%	75%	100%	100%	100%	0%	100%	0%	0%	0%	0%
22	22 - Health professionals	0%	0%	0%	0%	0%	0%	0%	0%	0%	0%	0%	0%	0%	0%	0%	0%	0%	100%	0%	0%
23	23 - Teaching professionals	90%	100%	90%	90%	90%	0%	0%	100%	0%	75%	75%	100%	100%	100%	0%	100%	100%	0%	0%	0%
24	24 - Business and administration professionals	0%	90%	90%	90%	0%	0%	0%	50%	0%	75%	75%	100%	100%	100%	0%	100%	0%	0%	0%	0%
25	25 - Information and communications technology professionals	0%	90%	90%	90%	0%	0%	0%	100%	0%	75%	75%	100%	100%	100%	0%	100%	0%	0%	0%	0%
26	26 - Legal, social and cultural professionals	0%	25%	25%	25%	0%	0%	0%	25%	0%	25%	25%	25%	25%	25%	0%	25%	100%	0%	0%	0%
31	31 - Science and engineering associate professionals	0%	90%	90%	90%	0%	0%	0%	50%	0%	75%	75%	100%	100%	100%	0%	100%	0%	0%	0%	0%
32	32 - Health associate professionals	0%	0%	0%	0%	0%	0%	0%	0%	0%	0%	0%	0%	0%	0%	0%	0%	0%	0%	0%	0%
33	33 - Business and administration associate professionals	0%	90%	90%	90%	0%	0%	0%	50%	0%	75%	75%	100%	100%	100%	0%	100%	0%	0%	0%	0%
34	34 - Legal, social, cultural and related associate professionals	0%	25%	25%	25%	0%	0%	0%	25%	0%	25%	25%	25%	25%	25%	0%	25%	0%	0%	0%	0%
35	35 - Information and communications technicians	0%	90%	90%	90%	0%	0%	0%	50%	0%	75%	75%	100%	100%	100%	0%	100%	0%	0%	0%	0%
41	41 - General and keyboard clerks	0%	90%	90%	90%	0%	0%	0%	50%	0%	75%	75%	100%	100%	100%	0%	100%	0%	0%	0%	0%
42	42 - Customer services clerks	0%	90%	90%	90%	0%	0%	0%	100%	0%	75%	75%	100%	100%	100%	0%	100%	0%	0%	0%	0%
43	43 - Numerical and material recording clerks	0%	90%	90%	90%	0%	0%	0%	50%	0%	75%	75%	100%	100%	100%	0%	100%	0%	0%	0%	0%
44	44 - Other clerical support workers	0%	90%	90%	90%	90%	0%	0%	100%	0%	75%	75%	100%	100%	100%	0%	100%	0%	0%	0%	0%
51	51 - Personal service workers	0%	0%	0%	0%	0%	0%	0%	0%	0%	0%	0%	0%	0%	0%	0%	0%	100%	0%	0%	0%
52	52 - Sales workers	50%	25%	25%	25%	25%	0%	0%	25%	0%	100%	50%	0%	0%	0%	0%	25%	100%	0%	0%	0%
53	53 - Personal care workers	0%	90%	90%	90%	90%	0%	0%	100%	0%	75%	75%	100%	100%	100%	0%	100%	0%	0%	0%	0%
54	54 - Protective services workers	0%	90%	90%	90%	90%	0%	0%	100%	0%	75%	75%	100%	100%	100%	0%	100%	0%	0%	0%	0%
61	61 - Market-oriented skilled agricultural workers	0%	90%	90%	90%	90%	0%	0%	100%	0%	75%	75%	0%	0%	0%	0%	100%	100%	0%	0%	0%
62	62 - Market-oriented skilled forestry, fishery and hunting workers	0%	90%	90%	90%	90%	0%	0%	100%	0%	75%	75%	100%	100%	100%	0%	100%	100%	0%	0%	0%
63	63 - Subsistence farmers, fishers, hunters and gatherers	0%	0%	0%	0%	0%	0%	0%	0%	0%	0%	0%	0%	0%	0%	0%	0%	0%	0%	0%	0%
71	71 - Building and related trades workers, excluding electricians	0%	90%	90%	90%	90%	0%	0%	100%	0%	75%	75%	100%	100%	100%	0%	100%	100%	0%	0%	0%
72	72 - Metal, machinery and related trades workers	0%	90%	90%	90%	90%	0%	0%	100%	0%	75%	75%	100%	100%	100%	0%	100%	100%	0%	0%	0%
73	73 - Handicraft and printing workers	0%	90%	90%	90%	90%	0%	0%	100%	0%	75%	75%	100%	100%	100%	0%	100%	100%	0%	0%	0%
74	74 - Electrical and electronic trades workers	0%	90%	90%	90%	90%	0%	0%	100%	0%	75%	75%	100%	100%	100%	0%	100%	0%	0%	0%	0%
75	75 - Food processing, wood working, garment and other craft and related trades workers	0%	90%	90%	90%	90%	0%	0%	100%	75%	75%	75%	100%	100%	100%	0%	0%	100%	0%	0%	0%
81	81 - Stationary plant and machine operators	0%	90%	90%	90%	90%	0%	0%	100%	0%	75%	75%	100%	100%	100%	0%	100%	100%	0%	0%	0%
82	82 - Assemblers	0%	90%	90%	90%	90%	0%	0%	100%	0%	75%	75%	100%	100%	100%	0%	100%	100%	0%	0%	0%
83	83 - Drivers and mobile plant operators	10%	90%	90%	90%	90%	0%	0%	50%	75%	75%	75%	100%	100%	100%	0%	100%	50%	0%	0%	0%
91	91 - Cleaners and helpers	10%	90%	90%	90%	90%	0%	0%	100%	0%	75%	75%	100%	100%	100%	0%	0%	0%	0%	0%	0%
92	92 - Agricultural, forestry and fishery labourers	10%	90%	90%	90%	90%	0%	0%	50%	0%	75%	75%	100%	100%	100%	0%	100%	100%	0%	0%	0%
93	93 - Labourers in mining, construction, manufacturing and transport	0%	90%	90%	90%	90%	0%	0%	75%	0%	75%	75%	100%	100%	100%	0%	100%	100%	0%	0%	0%
94	94 - Food preparation assistants	0%	90%	90%	90%	90%	0%	0%	75%	75%	75%	75%	100%	100%	100%	0%	100%	0%	0%	0%	0%
95	95 - Street and related sales and service workers	0%	0%	0%	0%	0%	0%	0%	0%	0%	0%	0%	0%	0%	0%	0%	0%	0%	0%	0%	0%
96	96 - Refuse workers and other elementary workers	0%	90%	90%	90%	90%	0%	50%	100%	0%	75%	75%	100%	100%	100%	0%	100%	0%	0%	0%	0%

South Africa

The first COVID case was confirmed in South Africa on 5th March 2020. A nationwide lockdown was put in place from midnight March 26, with only critical workers and transport services, the banking sector, essential food and medicine production, and retail, operating. On May 1, 2020, a phased lifting of the lockdown began, allowing a few sectors to resume operation and others only partially. On May 13, a further relaxation of the lockdown was announced effective June 1. Most economic activities reopened under strict health and social distancing practices except for high-risk ones (i.e., restaurants, bars, taverns, accommodation, domestic air travel, conferences, events, entertainment, sporting activities, and personal services). Remote work is still encouraged where possible.

In May 2020, South Africa introduced a Covid-19 Temporary Employer-Employee Relief Scheme (TERS) for employers who were struggling to pay salaries due to loss of business during the lockdown. The COVID-19 TERS was aimed at preventing job losses and mass retrenchment of workers. The country further introduced a Special COVID-19 social relief of distress (SRD) grant which paid ZAR 350 (USD 20) for unemployed citizens and residents. Despite these measures, unemployment rates and poverty in South Africa still increased. A National Income Dynamics Study Coronavirus Rapid Mobile Survey conducted in June reported that more than 3 million people had lost their jobs and one a of breadwinners did not earn any income in April. Most of the job losses were among the most vulnerable low-income and grant receiving households. The South African economy was, however, already before the COVID-19 pandemic and the lockdowns only compounded the unemployment problem. Although all lockdown restrictions finally lifted on 20 September, the South African government expects a slow recovery of the economy, predicting an 8.2% contraction which will keep unemployment and poverty rates high.

Percent of jobs that were essential during lockdown

		A. Agriculture; forestry and fishing	B. Mining and quarrying	C. Manufacturing	D. Electricity; gas, steam and air	E. Water supply; sewerage, waste management and remediation	F. Construction	G. Wholesale and retail trade; repair of motor vehicles and	H. Transportation and storage	I. Accommodation and food service	J. Information and communicat	K. Financial and insurance activities	L. Real estate activities	M. Professional, scientific and	N. Administrative and support	O. Public administration and defence;	P. Education	Q. Human health and social work activities	R. Arts, entertainment and recreation	S. Other service activities	T. Activities of households as employers; undifferentiated goods- and services-producing activities
**Isco\ Nace**	**Occupation description**	**A**	**B**	**C**	**D**	**E**	**F**	**G**	**H**	**I**	**J**	**K**	**L**	**M**	**N**	**O**	**P**	**Q**	**R**	**S**	**T**
**11**	11 - Chief executives, senior officials and legislators	100%	50%	10%	10%	10%	0%	0%	50%	0%	25%	25%	0%	0%	0%	100%	0%	100%	0%	0%	0%
**12**	12 - Administrative and commercial managers	100%	50%	10%	10%	10%	0%	0%	50%	0%	25%	25%	0%	0%	0%	100%	0%	100%	0%	0%	0%
**13**	13 - Production and specialised services managers	100%	50%	10%	10%	10%	0%	0%	50%	0%	25%	25%	0%	0%	0%	100%	0%	100%	0%	0%	0%
**14**	14 - Hospitality, retail and other services managers	100%	50%	10%	10%	10%	0%	0%	50%	0%	25%	25%	0%	0%	0%	100%	0%	100%	0%	0%	0%
**21**	21 - Science and engineering professionals	100%	50%	10%	10%	10%	0%	0%	50%	0%	25%	25%	0%	0%	0%	100%	0%	100%	0%	0%	0%
**22**	22 - Health professionals	100%	100%	100%	100%	100%	100%	100%	100%	100%	100%	100%	100%	100%	100%	100%	100%	100%	100%	100%	100%
**23**	23 - Teaching professionals	100%	50%	10%	10%	10%	0%	0%	0%	0%	25%	25%	0%	0%	0%	100%	0%	0%	0%	0%	0%
**24**	24 - Business and administration professionals	100%	50%	10%	10%	10%	0%	0%	50%	0%	25%	25%	0%	0%	0%	100%	0%	100%	0%	0%	0%
**25**	25 - Information and communications technology professionals	100%	50%	10%	10%	10%	0%	0%	0%	0%	25%	25%	0%	0%	0%	100%	0%	100%	0%	0%	0%
**26**	26 - Legal, social and cultural professionals	100%	50%	0%	0%	0%	0%	0%	0%	0%	0%	0%	0%	0%	0%	100%	0%	0%	0%	0%	0%
**31**	31 - Science and engineering associate professionals	100%	50%	10%	10%	10%	0%	0%	50%	0%	25%	25%	0%	0%	0%	100%	0%	100%	0%	0%	0%
**32**	32 - Health associate professionals	100%	100%	100%	100%	100%	100%	100%	100%	100%	100%	100%	100%	100%	100%	100%	100%	100%	100%	100%	100%
**33**	33 - Business and administration associate professionals	100%	50%	10%	10%	10%	0%	0%	50%	0%	25%	25%	0%	0%	0%	100%	0%	100%	0%	0%	0%
**34**	34 - Legal, social, cultural and related associate professionals	100%	50%	0%	0%	0%	0%	0%	0%	0%	0%	0%	0%	0%	0%	100%	0%	100%	0%	0%	0%
**35**	35 - Information and communications technicians	100%	50%	10%	10%	10%	0%	0%	50%	0%	25%	25%	0%	0%	0%	100%	0%	100%	0%	0%	0%
**41**	41 - General and keyboard clerks	100%	50%	10%	10%	10%	0%	0%	50%	0%	25%	25%	0%	0%	0%	100%	0%	100%	0%	0%	0%
**42**	42 - Customer services clerks	100%	50%	10%	10%	10%	0%	0%	0%	0%	25%	25%	0%	0%	0%	100%	0%	100%	0%	0%	0%
**43**	43 - Numerical and material recording clerks	100%	50%	10%	10%	10%	0%	0%	50%	0%	25%	25%	0%	0%	0%	100%	0%	100%	0%	0%	0%
**44**	44 - Other clerical support workers	100%	50%	10%	10%	10%	0%	0%	0%	0%	25%	25%	0%	0%	0%	100%	0%	100%	0%	0%	0%
**51**	51 - Personal service workers	100%	50%	0%	0%	0%	0%	0%	0%	0%	0%	0%	0%	0%	0%	100%	0%	0%	0%	0%	0%
**52**	52 - Sales workers	100%	50%	0%	0%	0%	0%	0%	0%	0%	0%	0%	0%	0%	0%	100%	0%	0%	0%	0%	0%
**53**	53 - Personal care workers	100%	50%	10%	10%	10%	0%	100%	0%	0%	25%	25%	0%	0%	0%	100%	0%	100%	0%	0%	0%
**54**	54 - Protective services workers	100%	50%	10%	10%	10%	0%	0%	0%	0%	25%	25%	0%	0%	0%	100%	0%	100%	0%	0%	0%
**61**	61 - Market-oriented skilled agricultural workers	100%	50%	10%	10%	10%	0%	0%	0%	0%	25%	25%	100%	100%	100%	100%	0%	0%	100%	100%	100%
**62**	62 - Market-oriented skilled forestry, fishery and hunting workers	100%	50%	10%	10%	10%	0%	0%	0%	0%	25%	25%	0%	0%	0%	100%	0%	0%	0%	0%	0%
**63**	63 - Subsistence farmers, fishers, hunters and gatherers	100%	100%	100%	100%	100%	100%	100%	100%	100%	100%	100%	100%	100%	100%	100%	100%	100%	100%	100%	100%
**71**	71 - Building and related trades workers, excluding electricians	100%	50%	10%	10%	10%	0%	0%	0%	0%	25%	25%	0%	0%	0%	100%	0%	0%	0%	0%	0%
**72**	72 - Metal, machinery and related trades workers	100%	50%	10%	10%	10%	0%	0%	0%	0%	25%	25%	0%	0%	0%	100%	0%	0%	0%	0%	0%
**73**	73 - Handicraft and printing workers	100%	50%	10%	10%	10%	0%	0%	0%	0%	25%	25%	0%	0%	0%	100%	0%	0%	0%	0%	0%
**74**	74 - Electrical and electronic trades workers	100%	50%	10%	10%	10%	0%	0%	0%	0%	25%	25%	0%	0%	0%	100%	0%	100%	0%	0%	0%
**75**	75 - Food processing, wood working, garment and other craft and related trades workers	100%	50%	10%	10%	10%	0%	0%	0%	25%	25%	25%	0%	0%	0%	100%	0%	0%	0%	0%	0%
**81**	81 - Stationary plant and machine operators	100%	50%	10%	10%	10%	0%	0%	0%	0%	25%	25%	0%	0%	0%	100%	0%	0%	0%	0%	0%
**82**	82 - Assemblers	100%	50%	10%	10%	10%	0%	0%	0%	0%	25%	25%	0%	0%	0%	100%	0%	0%	0%	0%	0%
**83**	83 - Drivers and mobile plant operators	100%	50%	10%	10%	10%	0%	0%	50%	25%	25%	25%	0%	0%	0%	100%	0%	50%	0%	0%	0%
**91**	91 - Cleaners and helpers	100%	50%	10%	10%	10%	0%	0%	0%	0%	25%	25%	0%	0%	0%	100%	0%	100%	0%	0%	0%
**92**	92 - Agricultural, forestry and fishery labourers	100%	50%	10%	10%	10%	0%	0%	50%	0%	25%	25%	0%	0%	0%	100%	0%	0%	0%	0%	0%
**93**	93 - Labourers in mining, construction, manufacturing and transport	100%	50%	10%	10%	10%	0%	0%	25%	0%	25%	25%	0%	0%	0%	100%	0%	0%	0%	0%	0%
**94**	94 - Food preparation assistants	100%	50%	10%	10%	10%	0%	100%	25%	25%	25%	25%	0%	0%	0%	100%	0%	100%	0%	0%	0%
**95**	95 - Street and related sales and service workers	100%	50%	10%	10%	10%	0%	0%	0%	0%	25%	25%	0%	0%	0%	100%	0%	100%	0%	0%	0%
**96**	96 - Refuse workers and other elementary workers	100%	50%	10%	10%	10%	0%	0%	0%	0%	25%	25%	0%	0%	0%	100%	0%	100%	0%	0%	0%

South Africa

Percent of jobs that were closed during lockdown

Isco\ Nace	Occupation description	A. Agriculture; forestry and fishing	B. Mining and quarrying	C. Manufacturing	D. Electricity; gas, steam and air	E. Water supply; sewerage, waste management and remediation	F. Construction	G. Wholesale and retail trade; repair of motor vehicles and	H. Transportation and storage	I. Accommodation and food service	J. Information and communicat	K. Financial and insurance activities	L. Real estate activities	M. Professional, scientific and	N. Administrative and support	O. Public administration and defence;	P. Education	Q. Human health and social work activities	R. Arts, entertainment and recreation	S. Other service activities	T. Activities of households as employers; undifferentiated goods- and services-producing activities
		A	B	C	D	E	F	G	H	I	J	K	L	M	N	O	P	Q	R	S	T
11	11 - Chief executives, senior officials and legislators	0%	0%	0%	0%	0%	0%	100%	0%	100%	0%	0%	0%	0%	0%	0%	0%	0%	100%	100%	100%
12	12 - Administrative and commercial managers	0%	0%	0%	0%	0%	0%	100%	0%	100%	0%	0%	0%	0%	0%	0%	0%	0%	100%	100%	100%
13	13 - Production and specialised services managers	0%	0%	0%	0%	0%	0%	100%	0%	100%	0%	0%	0%	0%	0%	0%	0%	0%	100%	100%	100%
14	14 - Hospitality, retail and other services managers	0%	0%	0%	0%	0%	0%	100%	0%	100%	0%	0%	0%	0%	0%	0%	0%	0%	100%	100%	100%
21	21 - Science and engineering professionals	0%	0%	0%	0%	0%	0%	100%	0%	100%	0%	0%	0%	0%	0%	0%	0%	0%	100%	100%	100%
22	22 - Health professionals	0%	0%	0%	0%	0%	0%	0%	0%	0%	0%	0%	0%	0%	0%	0%	0%	0%	0%	0%	0%
23	23 - Teaching professionals	0%	0%	0%	0%	0%	0%	100%	0%	100%	0%	0%	0%	0%	0%	0%	0%	0%	100%	100%	100%
24	24 - Business and administration professionals	0%	0%	0%	0%	0%	0%	100%	0%	100%	0%	0%	0%	0%	0%	0%	0%	0%	100%	100%	100%
25	25 - Information and communications technology professionals	0%	0%	0%	0%	0%	0%	100%	0%	100%	0%	0%	0%	0%	0%	0%	0%	0%	100%	100%	100%
26	26 - Legal, social and cultural professionals	0%	50%	75%	75%	75%	75%	75%	75%	100%	75%	75%	75%	75%	75%	0%	75%	0%	100%	100%	100%
31	31 - Science and engineering associate professionals	0%	0%	0%	0%	0%	0%	100%	0%	100%	0%	0%	0%	0%	0%	0%	0%	0%	100%	100%	100%
32	32 - Health associate professionals	0%	0%	0%	0%	0%	0%	0%	0%	0%	0%	0%	0%	0%	0%	0%	0%	0%	0%	0%	0%
33	33 - Business and administration associate professionals	0%	0%	0%	0%	0%	0%	100%	0%	100%	0%	0%	0%	0%	0%	0%	0%	0%	100%	100%	100%
34	34 - Legal, social, cultural and related associate professionals	0%	50%	75%	75%	75%	75%	75%	75%	100%	75%	75%	75%	75%	75%	0%	75%	0%	100%	100%	100%
35	35 - Information and communications technicians	0%	0%	0%	0%	0%	0%	100%	0%	100%	0%	0%	0%	0%	0%	0%	0%	0%	100%	100%	100%
41	41 - General and keyboard clerks	0%	0%	0%	0%	0%	0%	100%	0%	100%	0%	0%	0%	0%	0%	0%	0%	0%	100%	100%	100%
42	42 - Customer services clerks	0%	0%	0%	0%	0%	0%	100%	0%	100%	0%	0%	0%	0%	0%	0%	0%	0%	100%	100%	100%
43	43 - Numerical and material recording clerks	0%	0%	0%	0%	0%	0%	100%	0%	100%	0%	0%	0%	0%	0%	0%	0%	0%	100%	100%	100%
44	44 - Other clerical support workers	0%	0%	0%	0%	0%	0%	100%	0%	100%	0%	0%	0%	0%	0%	0%	0%	0%	100%	100%	100%
51	51 - Personal service workers	0%	0%	100%	100%	100%	100%	100%	100%	100%	100%	100%	100%	100%	100%	0%	100%	0%	100%	100%	100%
52	52 - Sales workers	0%	0%	75%	75%	75%	75%	75%	75%	0%	0%	0%	100%	100%	100%	0%	75%	0%	100%	100%	100%
53	53 - Personal care workers	0%	0%	0%	0%	0%	0%	0%	0%	100%	0%	0%	0%	0%	0%	0%	0%	0%	100%	100%	100%
54	54 - Protective services workers	0%	0%	0%	0%	0%	0%	100%	0%	100%	0%	0%	0%	0%	0%	0%	0%	0%	100%	100%	100%
61	61 - Market-oriented skilled agricultural workers	0%	0%	0%	0%	0%	0%	100%	0%	100%	0%	0%	0%	0%	0%	0%	0%	0%	0%	0%	0%
62	62 - Market-oriented skilled forestry, fishery and hunting workers	0%	0%	0%	0%	0%	0%	100%	0%	100%	0%	0%	0%	0%	0%	0%	0%	0%	100%	100%	100%
63	63 - Subsistence farmers, fishers, hunters and gatherers	0%	0%	0%	0%	0%	0%	0%	0%	0%	0%	0%	0%	0%	0%	0%	0%	0%	0%	0%	0%
71	71 - Building and related trades workers, excluding electricians	0%	0%	0%	0%	0%	0%	100%	0%	100%	0%	0%	0%	0%	0%	0%	0%	0%	100%	100%	100%
72	72 - Metal, machinery and related trades workers	0%	0%	0%	0%	0%	0%	100%	0%	100%	0%	0%	0%	0%	0%	0%	0%	0%	100%	100%	100%
73	73 - Handicraft and printing workers	0%	0%	0%	0%	0%	0%	100%	0%	100%	0%	0%	0%	0%	0%	0%	0%	0%	100%	100%	100%
74	74 - Electrical and electronic trades workers	0%	0%	0%	0%	0%	0%	100%	0%	100%	0%	0%	0%	0%	0%	0%	0%	0%	100%	100%	100%
75	75 - Food processing, wood working, garment and other craft and related trades workers	0%	0%	0%	0%	0%	0%	100%	0%	0%	0%	0%	0%	0%	0%	0%	100%	0%	100%	100%	100%
81	81 - Stationary plant and machine operators	0%	0%	0%	0%	0%	0%	100%	0%	100%	0%	0%	0%	0%	0%	0%	0%	0%	100%	100%	100%
82	82 - Assemblers	0%	0%	0%	0%	0%	0%	100%	0%	100%	0%	0%	0%	0%	0%	0%	0%	0%	100%	100%	100%
83	83 - Drivers and mobile plant operators	0%	0%	0%	0%	0%	0%	100%	0%	0%	0%	0%	0%	0%	0%	0%	0%	0%	100%	100%	100%
91	91 - Cleaners and helpers	0%	0%	0%	0%	0%	0%	100%	0%	100%	0%	0%	0%	0%	0%	0%	100%	0%	100%	100%	100%
92	92 - Agricultural, forestry and fishery labourers	0%	0%	0%	0%	0%	0%	100%	0%	100%	0%	0%	0%	0%	0%	0%	0%	0%	100%	100%	100%
93	93 - Labourers in mining, construction, manufacturing and transport	0%	0%	0%	0%	0%	0%	100%	0%	100%	0%	0%	0%	0%	0%	0%	0%	0%	100%	100%	100%
94	94 - Food preparation assistants	0%	0%	0%	0%	0%	0%	0%	0%	0%	0%	0%	0%	0%	0%	0%	0%	0%	100%	100%	100%
95	95 - Street and related sales and service workers	0%	0%	0%	0%	0%	0%	100%	0%	100%	0%	0%	0%	0%	0%	0%	0%	0%	100%	100%	100%
96	96 - Refuse workers and other elementary workers	0%	0%	0%	0%	0%	0%	100%	0%	100%	0%	0%	0%	0%	0%	0%	0%	0%	100%	100%	100%

South Africa

Percent of jobs subject to teleworking during lockdown

Isco\ Nace	Occupation description	A. Agriculture; forestry and fishing	B. Mining and quarrying	C. Manufacturing	D. Electricity; gas, steam and air	E. Water supply; sewerage, waste management and remediation	F. Construction	G. Wholesale and retail trade; repair of motor vehicles and	H. Transportation and storage	I. Accommodation and food service	J. Information and communicat	K. Financial and insurance activities	L. Real estate activities	M. Professional, scientific and	N. Administrative and support	O. Public administration and defence;	P. Education	Q. Human health and social work activities	R. Arts, entertainment and recreation	S. Other service activities	T. Activities of households as employers; undifferentiated goods- and services-producing activities
		A	B	C	D	E	F	G	H	I	J	K	L	M	N	O	P	Q	R	S	T
11	11 - Chief executives, senior officials and legislators	0%	50%	90%	90%	90%	100%	0%	50%	0%	75%	75%	100%	100%	100%	0%	100%	0%	0%	0%	0%
12	12 - Administrative and commercial managers	0%	50%	90%	90%	90%	100%	0%	50%	0%	75%	75%	100%	100%	100%	0%	100%	0%	0%	0%	0%
13	13 - Production and specialised services managers	0%	50%	90%	90%	90%	100%	0%	50%	0%	75%	75%	100%	100%	100%	0%	100%	0%	0%	0%	0%
14	14 - Hospitality, retail and other services managers	0%	50%	90%	90%	90%	100%	0%	50%	0%	75%	75%	100%	100%	100%	0%	100%	0%	0%	0%	0%
21	21 - Science and engineering professionals	0%	50%	90%	90%	90%	100%	0%	50%	0%	75%	75%	100%	100%	100%	0%	100%	0%	0%	0%	0%
22	22 - Health professionals	0%	0%	0%	0%	0%	0%	0%	0%	0%	0%	0%	0%	0%	0%	0%	0%	0%	0%	0%	0%
23	23 - Teaching professionals	0%	50%	90%	90%	90%	100%	0%	100%	0%	75%	75%	100%	100%	100%	0%	100%	100%	0%	0%	0%
24	24 - Business and administration professionals	0%	50%	90%	90%	90%	100%	0%	50%	0%	75%	75%	100%	100%	100%	0%	100%	0%	0%	0%	0%
25	25 - Information and communications technology professionals	0%	50%	90%	90%	90%	100%	0%	100%	0%	75%	75%	100%	100%	100%	0%	100%	0%	0%	0%	0%
26	26 - Legal, social and cultural professionals	0%	0%	25%	25%	25%	25%	25%	25%	0%	25%	25%	25%	25%	25%	0%	25%	100%	0%	0%	0%
31	31 - Science and engineering associate professionals	0%	50%	90%	90%	90%	100%	0%	50%	0%	75%	75%	100%	100%	100%	0%	100%	0%	0%	0%	0%
32	32 - Health associate professionals	0%	0%	0%	0%	0%	0%	0%	0%	0%	0%	0%	0%	0%	0%	0%	0%	0%	0%	0%	0%
33	33 - Business and administration associate professionals	0%	50%	90%	90%	90%	100%	0%	50%	0%	75%	75%	100%	100%	100%	0%	100%	0%	0%	0%	0%
34	34 - Legal, social, cultural and related associate professionals	0%	0%	25%	25%	25%	25%	25%	25%	0%	25%	25%	25%	25%	25%	0%	25%	0%	0%	0%	0%
35	35 - Information and communications technicians	0%	50%	90%	90%	90%	100%	0%	50%	0%	75%	75%	100%	100%	100%	0%	100%	0%	0%	0%	0%
41	41 - General and keyboard clerks	0%	50%	90%	90%	90%	100%	0%	50%	0%	75%	75%	100%	100%	100%	0%	100%	0%	0%	0%	0%
42	42 - Customer services clerks	0%	50%	90%	90%	90%	100%	0%	100%	0%	75%	75%	100%	100%	100%	0%	100%	0%	0%	0%	0%
43	43 - Numerical and material recording clerks	0%	50%	90%	90%	90%	100%	0%	50%	0%	75%	75%	100%	100%	100%	0%	100%	0%	0%	0%	0%
44	44 - Other clerical support workers	0%	50%	90%	90%	90%	100%	0%	100%	0%	75%	75%	100%	100%	100%	0%	100%	0%	0%	0%	0%
51	51 - Personal service workers	0%	50%	0%	0%	0%	0%	0%	0%	0%	0%	0%	0%	0%	0%	0%	0%	100%	0%	0%	0%
52	52 - Sales workers	0%	50%	25%	25%	25%	25%	25%	25%	100%	100%	100%	0%	0%	0%	0%	25%	100%	0%	0%	0%
53	53 - Personal care workers	0%	50%	90%	90%	90%	100%	0%	100%	0%	75%	75%	100%	100%	100%	0%	100%	0%	0%	0%	0%
54	54 - Protective services workers	0%	50%	90%	90%	90%	100%	0%	100%	0%	75%	75%	100%	100%	100%	0%	100%	0%	0%	0%	0%
61	61 - Market-oriented skilled agricultural workers	0%	50%	90%	90%	90%	100%	0%	100%	0%	75%	75%	0%	0%	0%	0%	100%	100%	0%	0%	0%
62	62 - Market-oriented skilled forestry, fishery and hunting workers	0%	50%	90%	90%	90%	100%	0%	100%	0%	75%	75%	100%	100%	100%	0%	100%	100%	0%	0%	0%
63	63 - Subsistence farmers, fishers, hunters and gatherers	0%	0%	0%	0%	0%	0%	0%	0%	0%	0%	0%	0%	0%	0%	0%	0%	0%	0%	0%	0%
71	71 - Building and related trades workers, excluding electricians	0%	50%	90%	90%	90%	100%	0%	100%	0%	75%	75%	100%	100%	100%	0%	100%	100%	0%	0%	0%
72	72 - Metal, machinery and related trades workers	0%	50%	90%	90%	90%	100%	0%	100%	0%	75%	75%	100%	100%	100%	0%	100%	100%	0%	0%	0%
73	73 - Handicraft and printing workers	0%	50%	90%	90%	90%	100%	0%	100%	0%	75%	75%	100%	100%	100%	0%	100%	100%	0%	0%	0%
74	74 - Electrical and electronic trades workers	0%	50%	90%	90%	90%	100%	0%	100%	0%	75%	75%	100%	100%	100%	0%	100%	0%	0%	0%	0%
75	75 - Food processing, wood working, garment and other craft and related trades workers	0%	50%	90%	90%	90%	100%	0%	100%	75%	75%	75%	100%	100%	100%	0%	0%	100%	0%	0%	0%
81	81 - Stationary plant and machine operators	0%	50%	90%	90%	90%	100%	0%	100%	0%	75%	75%	100%	100%	100%	0%	100%	100%	0%	0%	0%
82	82 - Assemblers	0%	50%	90%	90%	90%	100%	0%	100%	0%	75%	75%	100%	100%	100%	0%	100%	100%	0%	0%	0%
83	83 - Drivers and mobile plant operators	0%	50%	90%	90%	90%	100%	0%	50%	75%	75%	75%	100%	100%	100%	0%	100%	50%	0%	0%	0%
91	91 - Cleaners and helpers	0%	50%	90%	90%	90%	100%	0%	100%	0%	75%	75%	100%	100%	100%	0%	0%	0%	0%	0%	0%
92	92 - Agricultural, forestry and fishery labourers	0%	50%	90%	90%	90%	100%	0%	50%	0%	75%	75%	100%	100%	100%	0%	100%	100%	0%	0%	0%
93	93 - Labourers in mining, construction, manufacturing and transport	0%	50%	90%	90%	90%	100%	0%	75%	0%	75%	75%	100%	100%	100%	0%	100%	100%	0%	0%	0%
94	94 - Food preparation assistants	0%	50%	90%	90%	90%	100%	0%	75%	75%	75%	75%	100%	100%	100%	0%	100%	0%	0%	0%	0%
95	95 - Street and related sales and service workers	0%	50%	90%	90%	90%	100%	0%	100%	0%	75%	75%	100%	100%	100%	0%	100%	0%	0%	0%	0%
96	96 - Refuse workers and other elementary workers	0%	50%	90%	90%	90%	100%	0%	100%	0%	75%	75%	100%	100%	100%	0%	100%	0%	0%	0%	0%

Pakistan

The first two COVID cases were recorded in Pakistan on 26th February 2020. Starting on March 23, both the federal and provincial governments implemented measures to contain and mitigate the spread of the virus. These included selective quarantines, border closures with neighbouring countries, international and domestic travel restrictions, school and university closures, banning of public events, social distancing measures, and varying levels of lockdown across the country.

Since mid-April, the federal government, in coordination with provinces, has gradually eased lockdown arrangements, by allowing 'low-risk industries' to restart operation and 'small retail shops' to reopen with newly developed Standard Operating Procedures (SOPs). In addition, restrictions on domestic and international movements have been lifted (e.g. domestic flights, train services, and international flights have resumed).

Percent of jobs that were essential during lockdown

		A. Agriculture; forestry and fishing	B. Mining and quarrying	C. Manufacturing	D. Electricity; gas, steam and air	E. Water supply; sewerage, waste management and remediation	F. Construction	G. Wholesale and retail trade; repair of motor vehicles and	H. Transportation and storage	I. Accommodation and food service	J. Information and communicat	K. Financial and insurance activities	L. Real estate activities	M. Professional, scientific and	N. Administrative and support	O. Public administration and defence;	P. Education	Q. Human health and social work activities	R. Arts, entertainment and recreation	S. Other service activities	T. Activities of households as employers; undifferentiated goods- and services-producing activities
**Isco\ Nace**	**Occupation description**	**A**	**B**	**C**	**D**	**E**	**F**	**G**	**H**	**I**	**J**	**K**	**L**	**M**	**N**	**O**	**P**	**Q**	**R**	**S**	**T**
**11**	11 - Chief executives, senior officials and legislators	100%	10%	10%	10%	10%	0%	0%	50%	0%	25%	25%	0%	0%	0%	100%	0%	100%	0%	0%	0%
**12**	12 - Administrative and commercial managers	100%	10%	10%	10%	10%	0%	0%	50%	0%	25%	25%	0%	0%	0%	100%	0%	100%	0%	0%	0%
**13**	13 - Production and specialised services managers	100%	10%	10%	10%	10%	0%	0%	50%	0%	25%	25%	0%	0%	0%	100%	0%	100%	0%	0%	0%
**14**	14 - Hospitality, retail and other services managers	100%	10%	10%	10%	10%	0%	0%	50%	0%	25%	25%	0%	0%	0%	100%	0%	100%	0%	0%	0%
**21**	21 - Science and engineering professionals	100%	10%	10%	10%	10%	0%	0%	50%	0%	25%	25%	0%	0%	0%	100%	0%	100%	0%	0%	0%
**22**	22 - Health professionals	100%	100%	100%	100%	100%	100%	100%	100%	100%	100%	100%	100%	100%	100%	100%	100%	100%	100%	100%	100%
**23**	23 - Teaching professionals	100%	10%	10%	10%	10%	0%	0%	0%	0%	25%	25%	0%	0%	0%	100%	0%	0%	0%	0%	0%
**24**	24 - Business and administration professionals	100%	10%	10%	10%	10%	0%	0%	50%	0%	25%	25%	0%	0%	0%	100%	0%	100%	0%	0%	0%
**25**	25 - Information and communications technology professionals	100%	10%	10%	10%	10%	0%	0%	0%	0%	25%	25%	0%	0%	0%	100%	0%	100%	0%	0%	0%
**26**	26 - Legal, social and cultural professionals	100%	0%	0%	0%	0%	0%	0%	0%	0%	0%	0%	0%	0%	0%	100%	0%	0%	0%	0%	0%
**31**	31 - Science and engineering associate professionals	100%	10%	10%	10%	10%	0%	0%	50%	0%	25%	25%	0%	0%	0%	100%	0%	100%	0%	0%	0%
**32**	32 - Health associate professionals	100%	100%	100%	100%	100%	100%	100%	100%	100%	100%	100%	100%	100%	100%	100%	100%	100%	100%	100%	100%
**33**	33 - Business and administration associate professionals	100%	10%	10%	10%	10%	0%	0%	50%	0%	25%	25%	0%	0%	0%	100%	0%	100%	0%	0%	0%
**34**	34 - Legal, social, cultural and related associate professionals	100%	0%	0%	0%	0%	0%	0%	0%	0%	0%	0%	0%	0%	0%	100%	0%	100%	0%	0%	0%
**35**	35 - Information and communications technicians	100%	10%	10%	10%	10%	0%	0%	50%	0%	25%	25%	0%	0%	0%	100%	0%	100%	0%	0%	0%
**41**	41 - General and keyboard clerks	100%	10%	10%	10%	10%	0%	0%	50%	0%	25%	25%	0%	0%	0%	100%	0%	100%	0%	0%	0%
**42**	42 - Customer services clerks	100%	10%	10%	10%	10%	0%	0%	0%	0%	25%	25%	0%	0%	0%	100%	0%	100%	0%	0%	0%
**43**	43 - Numerical and material recording clerks	100%	10%	10%	10%	10%	0%	0%	50%	0%	25%	25%	0%	0%	0%	100%	0%	100%	0%	0%	0%
**44**	44 - Other clerical support workers	100%	10%	10%	10%	10%	0%	0%	0%	0%	25%	25%	0%	0%	0%	100%	0%	100%	0%	0%	0%
**51**	51 - Personal service workers	100%	0%	0%	0%	0%	0%	0%	0%	0%	0%	0%	0%	0%	0%	100%	0%	0%	0%	0%	0%
**52**	52 - Sales workers	100%	0%	0%	0%	0%	0%	0%	0%	0%	0%	0%	0%	0%	0%	100%	0%	0%	0%	0%	0%
**53**	53 - Personal care workers	100%	10%	10%	10%	10%	0%	100%	0%	0%	25%	25%	0%	0%	0%	100%	0%	100%	0%	0%	0%
**54**	54 - Protective services workers	100%	10%	10%	10%	10%	0%	0%	0%	0%	25%	25%	0%	0%	0%	100%	0%	100%	0%	0%	0%
**61**	61 - Market-oriented skilled agricultural workers	100%	10%	10%	10%	10%	0%	0%	0%	0%	25%	25%	100%	100%	100%	100%	0%	0%	100%	100%	100%
**62**	62 - Market-oriented skilled forestry, fishery and hunting workers	100%	10%	10%	10%	10%	0%	0%	0%	0%	25%	25%	0%	0%	0%	100%	0%	0%	0%	0%	0%
**63**	63 - Subsistence farmers, fishers, hunters and gatherers	100%	100%	100%	100%	100%	100%	100%	100%	100%	100%	100%	100%	100%	100%	100%	100%	100%	100%	100%	100%
**71**	71 - Building and related trades workers, excluding electricians	100%	10%	10%	10%	10%	0%	0%	0%	0%	25%	25%	0%	0%	0%	100%	0%	0%	0%	0%	0%
**72**	72 - Metal, machinery and related trades workers	100%	10%	10%	10%	10%	0%	0%	0%	0%	25%	25%	0%	0%	0%	100%	0%	0%	0%	0%	0%
**73**	73 - Handicraft and printing workers	100%	10%	10%	10%	10%	0%	0%	0%	0%	25%	25%	0%	0%	0%	100%	0%	0%	0%	0%	0%
**74**	74 - Electrical and electronic trades workers	100%	10%	10%	10%	10%	0%	0%	0%	0%	25%	25%	0%	0%	0%	100%	0%	100%	0%	0%	0%
**75**	75 - Food processing, wood working, garment and other craft and related trades workers	100%	10%	10%	10%	10%	0%	0%	0%	25%	25%	25%	0%	0%	0%	100%	0%	0%	0%	0%	0%
**81**	81 - Stationary plant and machine operators	100%	10%	10%	10%	10%	0%	0%	0%	0%	25%	25%	0%	0%	0%	100%	0%	0%	0%	0%	0%
**82**	82 - Assemblers	100%	10%	10%	10%	10%	0%	0%	0%	0%	25%	25%	0%	0%	0%	100%	0%	0%	0%	0%	0%
**83**	83 - Drivers and mobile plant operators	100%	10%	10%	10%	10%	0%	0%	50%	25%	25%	25%	0%	0%	0%	100%	0%	50%	0%	0%	0%
**91**	91 - Cleaners and helpers	100%	10%	10%	10%	10%	0%	0%	0%	0%	25%	25%	0%	0%	0%	100%	0%	100%	0%	0%	0%
**92**	92 - Agricultural, forestry and fishery labourers	100%	10%	10%	10%	10%	0%	0%	50%	0%	25%	25%	0%	0%	0%	100%	0%	0%	0%	0%	0%
**93**	93 - Labourers in mining, construction, manufacturing and transport	100%	10%	10%	10%	10%	0%	0%	25%	0%	25%	25%	0%	0%	0%	100%	0%	0%	0%	0%	0%
**94**	94 - Food preparation assistants	100%	10%	10%	10%	10%	0%	100%	25%	25%	25%	25%	0%	0%	0%	100%	0%	100%	0%	0%	0%
**95**	95 - Street and related sales and service workers	100%	10%	10%	10%	10%	0%	0%	0%	0%	25%	25%	0%	0%	0%	100%	0%	100%	0%	0%	0%
**96**	96 - Refuse workers and other elementary workers	100%	10%	10%	10%	10%	0%	0%	0%	0%	25%	25%	0%	0%	0%	100%	0%	100%	0%	0%	0%

Pakistan

Percent of jobs that were closed during lockdown

		A. Agriculture; forestry and fishing	B. Mining and quarrying	C. Manufacturing	D. Electricity; gas, steam and air	E. Water supply; sewerage, waste management and remediation	F. Construction	G. Wholesale and retail trade; repair of motor vehicles and	H. Transportation and storage	I. Accommodation and food service	J. Information and communicat	K. Financial and insurance activities	L. Real estate activities	M. Professional, scientific and	N. Administrative and support	O. Public administration and defence;	P. Education	Q. Human health and social work activities	R. Arts, entertainment and recreation	S. Other service activities	T. Activities of households as employers; undifferentiated goods- and services-producing activities
**Isco\ Nace**	**Occupation description**	**A**	**B**	**C**	**D**	**E**	**F**	**G**	**H**	**I**	**J**	**K**	**L**	**M**	**N**	**O**	**P**	**Q**	**R**	**S**	**T**
**11**	11 - Chief executives, senior officials and legislators	0%	0%	0%	0%	0%	0%	100%	0%	100%	0%	0%	0%	0%	0%	0%	0%	0%	100%	100%	100%
**12**	12 - Administrative and commercial managers	0%	0%	0%	0%	0%	0%	100%	0%	100%	0%	0%	0%	0%	0%	0%	0%	0%	100%	100%	100%
**13**	13 - Production and specialised services managers	0%	0%	0%	0%	0%	0%	100%	0%	100%	0%	0%	0%	0%	0%	0%	0%	0%	100%	100%	100%
**14**	14 - Hospitality, retail and other services managers	0%	0%	0%	0%	0%	0%	100%	0%	100%	0%	0%	0%	0%	0%	0%	0%	0%	100%	100%	100%
**21**	21 - Science and engineering professionals	0%	0%	0%	0%	0%	0%	100%	0%	100%	0%	0%	0%	0%	0%	0%	0%	0%	100%	100%	100%
**22**	22 - Health professionals	0%	0%	0%	0%	0%	0%	0%	0%	0%	0%	0%	0%	0%	0%	0%	0%	0%	0%	0%	0%
**23**	23 - Teaching professionals	0%	0%	0%	0%	0%	0%	100%	0%	100%	0%	0%	0%	0%	0%	0%	0%	0%	100%	100%	100%
**24**	24 - Business and administration professionals	0%	0%	0%	0%	0%	0%	100%	0%	100%	0%	0%	0%	0%	0%	0%	0%	0%	100%	100%	100%
**25**	25 - Information and communications technology professionals	0%	0%	0%	0%	0%	0%	100%	0%	100%	0%	0%	0%	0%	0%	0%	0%	0%	100%	100%	100%
**26**	26 - Legal, social and cultural professionals	0%	75%	75%	75%	75%	75%	75%	75%	100%	75%	75%	75%	75%	75%	0%	75%	0%	100%	100%	100%
**31**	31 - Science and engineering associate professionals	0%	0%	0%	0%	0%	0%	100%	0%	100%	0%	0%	0%	0%	0%	0%	0%	0%	100%	100%	100%
**32**	32 - Health associate professionals	0%	0%	0%	0%	0%	0%	0%	0%	0%	0%	0%	0%	0%	0%	0%	0%	0%	0%	0%	0%
**33**	33 - Business and administration associate professionals	0%	0%	0%	0%	0%	0%	100%	0%	100%	0%	0%	0%	0%	0%	0%	0%	0%	100%	100%	100%
**34**	34 - Legal, social, cultural and related associate professionals	0%	75%	75%	75%	75%	75%	75%	75%	100%	75%	75%	75%	75%	75%	0%	75%	0%	100%	100%	100%
**35**	35 - Information and communications technicians	0%	0%	0%	0%	0%	0%	100%	0%	100%	0%	0%	0%	0%	0%	0%	0%	0%	100%	100%	100%
**41**	41 - General and keyboard clerks	0%	0%	0%	0%	0%	0%	100%	0%	100%	0%	0%	0%	0%	0%	0%	0%	0%	100%	100%	100%
**42**	42 - Customer services clerks	0%	0%	0%	0%	0%	0%	100%	0%	100%	0%	0%	0%	0%	0%	0%	0%	0%	100%	100%	100%
**43**	43 - Numerical and material recording clerks	0%	0%	0%	0%	0%	0%	100%	0%	100%	0%	0%	0%	0%	0%	0%	0%	0%	100%	100%	100%
**44**	44 - Other clerical support workers	0%	0%	0%	0%	0%	0%	100%	0%	100%	0%	0%	0%	0%	0%	0%	0%	0%	100%	100%	100%
**51**	51 - Personal service workers	0%	100%	100%	100%	100%	100%	100%	100%	100%	100%	100%	100%	100%	100%	0%	100%	0%	100%	100%	100%
**52**	52 - Sales workers	0%	75%	75%	75%	75%	75%	75%	75%	0%	0%	0%	100%	100%	100%	0%	75%	0%	100%	100%	100%
**53**	53 - Personal care workers	0%	0%	0%	0%	0%	0%	0%	0%	100%	0%	0%	0%	0%	0%	0%	0%	0%	100%	100%	100%
**54**	54 - Protective services workers	0%	0%	0%	0%	0%	0%	100%	0%	100%	0%	0%	0%	0%	0%	0%	0%	0%	100%	100%	100%
**61**	61 - Market-oriented skilled agricultural workers	0%	0%	0%	0%	0%	0%	100%	0%	100%	0%	0%	0%	0%	0%	0%	0%	0%	0%	0%	0%
**62**	62 - Market-oriented skilled forestry, fishery and hunting workers	0%	0%	0%	0%	0%	0%	100%	0%	100%	0%	0%	0%	0%	0%	0%	0%	0%	100%	100%	100%
**63**	63 - Subsistence farmers, fishers, hunters and gatherers	0%	0%	0%	0%	0%	0%	0%	0%	0%	0%	0%	0%	0%	0%	0%	0%	0%	0%	0%	0%
**71**	71 - Building and related trades workers, excluding electricians	0%	0%	0%	0%	0%	0%	100%	0%	100%	0%	0%	0%	0%	0%	0%	0%	0%	100%	100%	100%
**72**	72 - Metal, machinery and related trades workers	0%	0%	0%	0%	0%	0%	100%	0%	100%	0%	0%	0%	0%	0%	0%	0%	0%	100%	100%	100%
**73**	73 - Handicraft and printing workers	0%	0%	0%	0%	0%	0%	100%	0%	100%	0%	0%	0%	0%	0%	0%	0%	0%	100%	100%	100%
**74**	74 - Electrical and electronic trades workers	0%	0%	0%	0%	0%	0%	100%	0%	100%	0%	0%	0%	0%	0%	0%	0%	0%	100%	100%	100%
**75**	75 - Food processing, wood working, garment and other craft and related trades workers	0%	0%	0%	0%	0%	0%	100%	0%	0%	0%	0%	0%	0%	0%	0%	100%	0%	100%	100%	100%
**81**	81 - Stationary plant and machine operators	0%	0%	0%	0%	0%	0%	100%	0%	100%	0%	0%	0%	0%	0%	0%	0%	0%	100%	100%	100%
**82**	82 - Assemblers	0%	0%	0%	0%	0%	0%	100%	0%	100%	0%	0%	0%	0%	0%	0%	0%	0%	100%	100%	100%
**83**	83 - Drivers and mobile plant operators	0%	0%	0%	0%	0%	0%	100%	0%	0%	0%	0%	0%	0%	0%	0%	0%	0%	100%	100%	100%
**91**	91 - Cleaners and helpers	0%	0%	0%	0%	0%	0%	100%	0%	100%	0%	0%	0%	0%	0%	0%	100%	0%	100%	100%	100%
**92**	92 - Agricultural, forestry and fishery labourers	0%	0%	0%	0%	0%	0%	100%	0%	100%	0%	0%	0%	0%	0%	0%	0%	0%	100%	100%	100%
**93**	93 - Labourers in mining, construction, manufacturing and transport	0%	0%	0%	0%	0%	0%	100%	0%	100%	0%	0%	0%	0%	0%	0%	0%	0%	100%	100%	100%
**94**	94 - Food preparation assistants	0%	0%	0%	0%	0%	0%	0%	0%	0%	0%	0%	0%	0%	0%	0%	0%	0%	100%	100%	100%
**95**	95 - Street and related sales and service workers	0%	0%	0%	0%	0%	0%	100%	0%	100%	0%	0%	0%	0%	0%	0%	0%	0%	100%	100%	100%
**96**	96 - Refuse workers and other elementary workers	0%	0%	0%	0%	0%	0%	100%	0%	100%	0%	0%	0%	0%	0%	0%	0%	0%	100%	100%	100%

Pakistan

Percent of jobs subject to teleworking during lockdown

		A. Agriculture; forestry and fishing	B. Mining and quarrying	C. Manufacturing	D. Electricity; gas, steam and air	E. Water supply; sewerage, waste management and remediation	F. Construction	G. Wholesale and retail trade; repair of motor vehicles and	H. Transportation and storage	I. Accommodation and food service	J. Information and communicat	K. Financial and insurance activities	L. Real estate activities	M. Professional, scientific and	N. Administrative and support	O. Public administration and defence;	P. Education	Q. Human health and social work activities	R. Arts, entertainment and recreation	S. Other service activities	T. Activities of households as employers; undifferentiated goods- and services-producing activities
**Isco\ Nace**	**Occupation description**	**A**	**B**	**C**	**D**	**E**	**F**	**G**	**H**	**I**	**J**	**K**	**L**	**M**	**N**	**O**	**P**	**Q**	**R**	**S**	**T**
**11**	11 - Chief executives, senior officials and legislators	0%	90%	90%	90%	90%	100%	0%	50%	0%	75%	75%	100%	100%	100%	0%	100%	0%	0%	0%	0%
**12**	12 - Administrative and commercial managers	0%	90%	90%	90%	90%	100%	0%	50%	0%	75%	75%	100%	100%	100%	0%	100%	0%	0%	0%	0%
**13**	13 - Production and specialised services managers	0%	90%	90%	90%	90%	100%	0%	50%	0%	75%	75%	100%	100%	100%	0%	100%	0%	0%	0%	0%
**14**	14 - Hospitality, retail and other services managers	0%	90%	90%	90%	90%	100%	0%	50%	0%	75%	75%	100%	100%	100%	0%	100%	0%	0%	0%	0%
**21**	21 - Science and engineering professionals	0%	90%	90%	90%	90%	100%	0%	50%	0%	75%	75%	100%	100%	100%	0%	100%	0%	0%	0%	0%
**22**	22 - Health professionals	0%	0%	0%	0%	0%	0%	0%	0%	0%	0%	0%	0%	0%	0%	0%	0%	0%	0%	0%	0%
**23**	23 - Teaching professionals	0%	90%	90%	90%	90%	100%	0%	100%	0%	75%	75%	100%	100%	100%	0%	100%	100%	0%	0%	0%
**24**	24 - Business and administration professionals	0%	90%	90%	90%	90%	100%	0%	50%	0%	75%	75%	100%	100%	100%	0%	100%	0%	0%	0%	0%
**25**	25 - Information and communications technology professionals	0%	90%	90%	90%	90%	100%	0%	100%	0%	75%	75%	100%	100%	100%	0%	100%	0%	0%	0%	0%
**26**	26 - Legal, social and cultural professionals	0%	25%	25%	25%	25%	25%	25%	25%	0%	25%	25%	25%	25%	25%	0%	25%	100%	0%	0%	0%
**31**	31 - Science and engineering associate professionals	0%	90%	90%	90%	90%	100%	0%	50%	0%	75%	75%	100%	100%	100%	0%	100%	0%	0%	0%	0%
**32**	32 - Health associate professionals	0%	0%	0%	0%	0%	0%	0%	0%	0%	0%	0%	0%	0%	0%	0%	0%	0%	0%	0%	0%
**33**	33 - Business and administration associate professionals	0%	90%	90%	90%	90%	100%	0%	50%	0%	75%	75%	100%	100%	100%	0%	100%	0%	0%	0%	0%
**34**	34 - Legal, social, cultural and related associate professionals	0%	25%	25%	25%	25%	25%	25%	25%	0%	25%	25%	25%	25%	25%	0%	25%	0%	0%	0%	0%
**35**	35 - Information and communications technicians	0%	90%	90%	90%	90%	100%	0%	50%	0%	75%	75%	100%	100%	100%	0%	100%	0%	0%	0%	0%
**41**	41 - General and keyboard clerks	0%	90%	90%	90%	90%	100%	0%	50%	0%	75%	75%	100%	100%	100%	0%	100%	0%	0%	0%	0%
**42**	42 - Customer services clerks	0%	90%	90%	90%	90%	100%	0%	100%	0%	75%	75%	100%	100%	100%	0%	100%	0%	0%	0%	0%
**43**	43 - Numerical and material recording clerks	0%	90%	90%	90%	90%	100%	0%	50%	0%	75%	75%	100%	100%	100%	0%	100%	0%	0%	0%	0%
**44**	44 - Other clerical support workers	0%	90%	90%	90%	90%	100%	0%	100%	0%	75%	75%	100%	100%	100%	0%	100%	0%	0%	0%	0%
**51**	51 - Personal service workers	0%	0%	0%	0%	0%	0%	0%	0%	0%	0%	0%	0%	0%	0%	0%	0%	100%	0%	0%	0%
**52**	52 - Sales workers	0%	25%	25%	25%	25%	25%	25%	25%	100%	100%	100%	0%	0%	0%	0%	25%	100%	0%	0%	0%
**53**	53 - Personal care workers	0%	90%	90%	90%	90%	100%	0%	100%	0%	75%	75%	100%	100%	100%	0%	100%	0%	0%	0%	0%
**54**	54 - Protective services workers	0%	90%	90%	90%	90%	100%	0%	100%	0%	75%	75%	100%	100%	100%	0%	100%	0%	0%	0%	0%
**61**	61 - Market-oriented skilled agricultural workers	0%	90%	90%	90%	90%	100%	0%	100%	0%	75%	75%	0%	0%	0%	0%	100%	100%	0%	0%	0%
**62**	62 - Market-oriented skilled forestry, fishery and hunting workers	0%	90%	90%	90%	90%	100%	0%	100%	0%	75%	75%	100%	100%	100%	0%	100%	100%	0%	0%	0%
**63**	63 - Subsistence farmers, fishers, hunters and gatherers	0%	0%	0%	0%	0%	0%	0%	0%	0%	0%	0%	0%	0%	0%	0%	0%	0%	0%	0%	0%
**71**	71 - Building and related trades workers, excluding electricians	0%	90%	90%	90%	90%	100%	0%	100%	0%	75%	75%	100%	100%	100%	0%	100%	100%	0%	0%	0%
**72**	72 - Metal, machinery and related trades workers	0%	90%	90%	90%	90%	100%	0%	100%	0%	75%	75%	100%	100%	100%	0%	100%	100%	0%	0%	0%
**73**	73 - Handicraft and printing workers	0%	90%	90%	90%	90%	100%	0%	100%	0%	75%	75%	100%	100%	100%	0%	100%	100%	0%	0%	0%
**74**	74 - Electrical and electronic trades workers	0%	90%	90%	90%	90%	100%	0%	100%	0%	75%	75%	100%	100%	100%	0%	100%	0%	0%	0%	0%
**75**	75 - Food processing, wood working, garment and other craft and related trades workers	0%	90%	90%	90%	90%	100%	0%	100%	75%	75%	75%	100%	100%	100%	0%	0%	100%	0%	0%	0%
**81**	81 - Stationary plant and machine operators	0%	90%	90%	90%	90%	100%	0%	100%	0%	75%	75%	100%	100%	100%	0%	100%	100%	0%	0%	0%
**82**	82 - Assemblers	0%	90%	90%	90%	90%	100%	0%	100%	0%	75%	75%	100%	100%	100%	0%	100%	100%	0%	0%	0%
**83**	83 - Drivers and mobile plant operators	0%	90%	90%	90%	90%	100%	0%	50%	75%	75%	75%	100%	100%	100%	0%	100%	50%	0%	0%	0%
**91**	91 - Cleaners and helpers	0%	90%	90%	90%	90%	100%	0%	100%	0%	75%	75%	100%	100%	100%	0%	0%	0%	0%	0%	0%
**92**	92 - Agricultural, forestry and fishery labourers	0%	90%	90%	90%	90%	100%	0%	50%	0%	75%	75%	100%	100%	100%	0%	100%	100%	0%	0%	0%
**93**	93 - Labourers in mining, construction, manufacturing and transport	0%	90%	90%	90%	90%	100%	0%	75%	0%	75%	75%	100%	100%	100%	0%	100%	100%	0%	0%	0%
**94**	94 - Food preparation assistants	0%	90%	90%	90%	90%	100%	0%	75%	75%	75%	75%	100%	100%	100%	0%	100%	0%	0%	0%	0%
**95**	95 - Street and related sales and service workers	0%	90%	90%	90%	90%	100%	0%	100%	0%	75%	75%	100%	100%	100%	0%	100%	0%	0%	0%	0%
**96**	96 - Refuse workers and other elementary workers	0%	90%	90%	90%	90%	100%	0%	100%	0%	75%	75%	100%	100%	100%	0%	100%	0%	0%	0%	0%
