# Supplementary material for: Exploring equity in health and poverty impacts of control measures for SARS-CoV-2 in six countries
Source: BMJ Glob Health. 2021 May 26;6(5):e005521. doi: 10.1136/bmjgh-2021-005521 (PMC8159665; doi:10.1136/bmjgh-2021-005521)
Supplement: Supplementary file 1 [file bmjgh-2021-005521supp001.pdf]

Supplemental File 1 Supplementary Results Tables

Supplementary Table 1 Total people in ISCO-08 occupation codes by country

	**Quintile 1**	**Quintile 2**	**Quintile 3**	**Quintile 4**	**Quintile 5**	**Total**
	n (thousands), %	n (thousands), %	n (thousands), %	n (thousands), %	n (thousands), %	n (thousands), %
**Chile**						
Total people in the labour force	1,293	1,293	1,292	1,293	1,291	6,463
Total unemployed people	115 (9%)	104 (8%)	114 (9%)	125 (10%)	74 (6%)	532 (8%)
Total people in 'essential' jobs	1,016 (79%)	772 (60%)	899 (70%)	355 (27%)	348 (27%)	3,391 (52%)
Total people in 'closed' jobs	93 (7%)	185 (14%)	162 (13%)	112 (9%)	14 (1%)	567 (9%)
Total people teleworking	(0%)	17 (1%)	8 (1%)	434 (34%)	622 (48%)	1,082 (17%)
Total people unable to telework	70 (5%)	215 (17%)	110 (8%)	267 (21%)	231 (18%)	893 (14%)
**Georgia**						
Total people in the labour force	336	334	334	334	333	1,671
Total unemployed people	217 (65%)	167 (50%)	179 (54%)	146 (44%)	122 (37%)	831 (50%)
Total people in 'essential' jobs	44 (13%)	61 (18%)	60 (18%)	87 (26%)	81 (24%)	333 (20%)
Total people in 'closed' jobs	30 (9%)	81 (24%)	23 (7%)	40 (12%)	19 (6%)	193 (12%)
Total people teleworking	(0%)	1 (0%)	22 (7%)	38 (11%)	98 (29%)	160 (10%)
Total people unable to telework	45 (13%)	25 (7%)	49 (15%)	23 (7%)	12 (4%)	154 (9%)
**South Africa**						
Total people in the labour force	3,222	3,221	3,222	3,221	3,223	16,108
Total unemployed people	766 (24%)	916 (28%)	209 (6%)	277 (9%)	165 (5%)	2,332 (14%)
Total people in 'essential' jobs	1,026 (32%)	335 (10%)	838 (26%)	1,002 (31%)	978 (30%)	4,179 (26%)
Total people in 'closed' jobs	917 (28%)	753 (23%)	352 (11%)	687 (21%)	541 (17%)	3,250 (20%)
Total people teleworking	(0%)	41 (1%)	64 (2%)	706 (22%)	1,228 (38%)	2,040 (13%)
Total people unable to telework	514 (16%)	1,176 (37%)	1,759 (55%)	549 (17%)	310 (10%)	4,308 (27%)
**Pakistan**						
Total people in the labour force	5,303	5,301	5,300	5,303	5,302	26,510
Total unemployed people	121 (2%)	113 (2%)	160 (3%)	189 (4%)	248 (5%)	831 (3%)
Total people in 'essential' jobs	1,836 (35%)	1,386 (26%)	633 (12%)	1,144 (22%)	1,448 (27%)	6,447 (24%)

Total people in 'closed' jobs	521 (10%)	494 (9%)	1,915 (36%)	852 (16%)	752 (14%)	4,533 (17%)
Total people teleworking	1 (0%)	137 (3%)	181 (3%)	329 (6%)	2,322 (44%)	2,971 (11%)
Total people unable to telework	2,823 (53%)	3,171 (60%)	2,411 (45%)	2,789 (53%)	532 (10%)	11,727 (44%)
**Philippines**						
Total people in the labour force	5,589	5,587	5,588	5,587	5,586	27,938
Total unemployed people	167 (3%)	185 (3%)	249 (4%)	219 (4%)	114 (2%)	934 (3%)
Total people in 'essential' jobs	2,150 (38%)	1,741 (31%)	524 (9%)	1,190 (21%)	2,314 (41%)	7,918 (28%)
Total people in 'closed' jobs	1,271 (23%)	1,203 (22%)	2,525 (45%)	1,307 (23%)	739 (13%)	7,046 (25%)
Total people teleworking	(0%)	63 (1%)	144 (3%)	817 (15%)	1,765 (32%)	2,788 (10%)
Total people unable to telework	2,001 (36%)	2,396 (43%)	2,146 (38%)	2,054 (37%)	655 (12%)	9,251 (33%)
**United Kingdom**						
Total people in the labour force	5,658	5,658	5,657	5,658	5,657	28,288
Total unemployed people	287 (5%)	231 (4%)	163 (3%)	92 (2%)	63 (1%)	837 (3%)
Total people in 'essential' jobs	1,409 (25%)	893 (16%)	1,996 (35%)	1,550 (27%)	2,461 (44%)	8,310 (29%)
Total people in 'closed' jobs	1,266 (22%)	1,711 (30%)	1,195 (21%)	735 (13%)	741 (13%)	5,648 (20%)
Total people teleworking	172 (3%)	306 (5%)	1,012 (18%)	2,365 (42%)	2,003 (35%)	5,857 (21%)
Total people unable to telework	2,524 (45%)	2,517 (44%)	1,291 (23%)	915 (16%)	389 (7%)	7,637 (27%)

Supplementary Table 2 Total people in ISCO-08 occupation codes by country

ISCO-08 Occupation code and description	ISEI Score	Total people in the workforce (including employees and unemployed)					
		Chile	Georgia	Pakistan	Philippines	South Africa	UK
**1. Managers**							
11 - Chief executives, senior officials and legislators	69	7.8K	5.1K	115.2K	497.1K	825.4K	103.0K
12 - Administrative and commercial managers	68	46.6K	91.6K	201.3K	257.9K	34.1K	905.3K
13 - Production and specialised services managers	60	42.4K	44.0K	316.6K	226.3K		1260.3K
14 - Hospitality, retail and other services managers	53			25.7K	101.0K	130.4K	871.0K
**2. Professionals**							
21 - Science and engineering professionals	70	204.8K	34.8K	121.6K	256.4K	92.1K	1130.3K
22 - Health professionals	66	109.6K	33.7K	119.0K	290.8K	351.7K	1262.3K
23 - Teaching professionals	63	301.4K	129.8K	1971.2K	1288.8K	286.7K	1403.0K
24 - Business and administration professionals	64	282.0K	91.4K	266.5K	249.7K		2059.6K
25 - Information and communications technology professionals	69			48.4K	141.6K		938.8K
26 - Legal, social and cultural professionals	66			338.8K	129.2K	327.9K	610.8K
**3. Technicians and associate professionals**							
31 - Science and engineering associate professionals	51	131.6K	39.2K	365.1K	300.9K	242.7K	528.3K
32 - Health associate professionals	47	157.9K	45.9K	474.1K	401.0K	313.8K	347.8K
33 - Business and administration associate professionals	52	66.7K	28.5K	631.6K	726.5K	459.9K	1861.5K
34 - Legal, social, cultural, related associate professionals	45	396.1K	89.1K	96.4K	158.0K		747.9K
35 - Information and communications technicians	53			150.5K	125.3K	1152.1K	220.0K
**4. Clerical support workers**							
41 - General and keyboard clerks	41	419.0K	71.4K	490.0K	945.7K	665.5K	219.6K
42 - Customer services clerks	40	246.5K	53.2K	160.8K	1072.1K		1195.2K
43 - Numerical and material recording clerks	43			184.0K	481.2K		648.5K
44 - Other clerical support workers	39			55.0K	159.3K	1909.8K	955.2K
**5. Service and sales workers**							
51 - Personal service workers	30	589.4K	150.7K	1069.5K	1253.7K	951.7K	1017.6K
52 - Sales workers	33	408.0K	80.9K	1661.6K	2525.1K		1691.2K
53 - Personal care workers	26			26.8K	414.7K		2038.9K
54 - Protective services workers	39			927.5K	995.7K		555.5K

**6. Skilled agricultural, forestry and fishery workers**							
61 - Market-oriented skilled agricultural workers	19	113.1K	37.4K	133.2K	1.1K	36.8K	128.6K
62 - Market-oriented skilled forestry, fishery, hunting workers	24	1.3K		20.1K	25.5K		20.7K
63 - Subsistence farmers, fishers, hunters and gatherers	10					686.3K	
**7. Craft and related trades workers**							
71 - Building and related trades workers, excluding electricians	34	338.0K	81.9K	1548.9K	1485.3K	779.8K	452.6K
72 - Metal, machinery and related trades workers	38	269.6K	69.6K	773.2K	575.1K	87.2K	679.3K
73 - Handicraft and printing workers	33	18.6K	7.2K	282.7K	169.9K	269.7K	98.1K
74 - Electrical and electronic trades workers	43	124.0K	63.3K	390.4K	331.7K		264.9K
75 - Food processing, wood working, garment and other craft and related trades	27			2103.6K	547.4K		251.1K
**8. Plant and machine operators, and assemblers**							
81 - Stationary plant and machine operators	29	42.8K	15.8K	1005.7K	342.5K	486.8K	319.0K
82 - Assemblers	29	128.2K	22.3K	12.0K	361.7K		120.0K
83 - Drivers and mobile plant operators	36	400.9K	128.7K	1498.5K	1709.5K	3088.3K	881.2K
**9. Elementary occupations**							
91 - Cleaners and helpers	17	915.8K	171.2K	1028.2K	1991.1K	1477.0K	580.0K
92 - Agricultural, forestry and fishery labourers	15	385.3K	16.3K	2615.1K	3246.3K	1452.8K	50.0K
93 - Labourers in mining, construction, manufacturing, transport	24	315.6K	68.1K	4714.9K	3325.8K		959.3K
94 - Food preparation assistants	20			10.6K	23.3K		23.3K
95 - Street and related sales and service workers	25			555.4K	581.4K		354.3K
96 - Refuse workers and other elementary workers	26				222.1K		534.0K

K, Thousand. ISEI, international socio-economic index.

Supplementary Table 3 Essential workers by country

ISCO-08 Occupation code and description	Chile	Georgia	Pakistan	Philippines	South Africa	UK
**1. Managers**	1.2K	1.2K	29.9K	270.3K	186.4K	25.1K
11 - Chief executives, senior officials and legislators	3.6K	21.4K	52.3K	140.2K	7.7K	220.3K
12 - Administrative and commercial managers	13.2K	10.3K	82.2K	123.1K		306.6K
13 - Production and specialised services managers			6.7K	54.9K	29.5K	211.9K
14 - Hospitality, retail and other services managers						
**2. Professionals**	37.1K	7.4K	10.9K	74.6K	20.7K	375.1K
21 - Science and engineering professionals	83.5K	22.3K	115.2K	286.2K	336.8K	1252.0K
22 - Health professionals	54.6K	16.3K	91.4K	260.8K	29.6K	309.1K
23 - Teaching professionals	51.0K	19.4K	23.9K	72.7K		683.4K
24 - Business and administration professionals			4.2K	41.0K		306.0K
25 - Information and communications technology professionals			9.8K	23.3K	27.1K	48.0K
26 - Legal, social and cultural professionals						
**3. Technicians and associate professionals**	21.5K	10.4K	164.9K	117.4K	70.4K	187.6K
31 - Science and engineering associate professionals	115.5K	26.6K	450.6K	391.4K	295.4K	340.3K
32 - Health associate professionals	10.9K	7.5K	285.4K	283.4K	133.4K	660.9K
33 - Business and administration associate professionals	64.7K	21.2K	38.9K	54.1K		208.1K
34 - Legal, social, cultural, related associate professionals			68.0K	48.9K	334.2K	78.1K
35 - Information and communications technicians						
**4. Clerical support workers**	75.2K	17.8K	211.0K	246.6K	171.4K	72.6K
41 - General and keyboard clerks	44.2K	11.5K	60.6K	257.6K		350.5K
42 - Customer services clerks			79.2K	125.5K		214.5K
43 - Numerical and material recording clerks			20.7K	38.3K	412.8K	280.2K
44 - Other clerical support workers						
**5. Service and sales workers**	226.2K	30.6K	153.3K	129.5K	62.3K	73.5K
51 - Personal service workers	201.2K	16.4K	238.2K	253.4K		122.1K
52 - Sales workers			15.8K	199.4K		807.3K
53 - Personal care workers			156.8K	117.8K		220.0K
54 - Protective services workers						
**6. Skilled agricultural, forestry and fishery workers**	95.8K	5.6K	81.8K	0.7K	34.5K	111.0K

61 - Market-oriented skilled agricultural workers	1.1K		8.5K	16.3K		5.7K
62 - Market-oriented skilled forestry, fishery, hunting workers					679.0K	
63 - Subsistence farmers, fishers, hunters and gatherers						
**7. Craft and related trades workers**	253.7K	10.9K	105.4K	69.7K	30.3K	45.8K
71 - Building and related trades workers, excluding electricians	199.8K	9.2K	52.6K	27.0K	3.4K	68.7K
72 - Metal, machinery and related trades workers	14.0K	1.0K	19.2K	8.0K	10.5K	9.9K
73 - Handicraft and printing workers	91.9K	8.5K	26.6K	15.8K		29.0K
74 - Electrical and electronic trades workers			145.7K	26.2K		25.4K
75 - Food processing, wood working, garment and other craft and related trades						
**8. Plant and machine operators, and assemblers**	33.8K	1.6K	133.6K	25.7K	60.6K	31.9K
81 - Stationary plant and machine operators	101.1K	2.3K	1.6K	27.1K		12.0K
82 - Assemblers	313.0K	20.7K	490.6K	505.8K	841.9K	221.5K
83 - Drivers and mobile plant operators						
**9. Elementary occupations**	705.9K	22.2K	346.5K	743.6K	204.6K	99.4K
91 - Cleaners and helpers	316.1K	2.1K	895.8K	1259.2K	196.6K	7.3K
92 - Agricultural, forestry and fishery labourers	260.6K	8.8K	1578.5K	1259.0K		122.5K
93 - Labourers in mining, construction, manufacturing, transport			3.6K	8.7K		4.0K
94 - Food preparation assistants			187.2K	217.1K		60.8K
95 - Street and related sales and service workers				97.3K		101.6K
96 - Refuse workers and other elementary workers	1.2K	1.2K	29.9K	270.3K	186.4K	25.1K

K, Thousand.

Supplementary Table 4 Closed jobs by country

ISCO-08 Occupation code and description	Chile	Georgia	Pakistan	Philippines	South Africa	UK
**1. Managers**	0.2K	0.5K	7.9K	93.1K	188.5K	21.8K
11 - Chief executives, senior officials and legislators	1.5K	8.7K	13.8K	48.3K	7.8K	192.0K
12 - Administrative and commercial managers	1.3K	4.2K	21.7K	42.4K		267.3K
13 - Production and specialised services managers			1.8K	18.9K	29.8K	184.7K
14 - Hospitality, retail and other services managers						
**2. Professionals**	1.9K	1.7K	15.9K	14.3K	4.3K	77.7K
21 - Science and engineering professionals	0.0B	0.0B	0.0B	0.0B	0.0B	0.0B
22 - Health professionals	2.8K	6.4K	258.3K	71.9K	13.4K	66.2K
23 - Teaching professionals	2.7K	4.5K	34.9K	13.9K		141.5K
24 - Business and administration professionals			6.3K	7.9K		64.5K
25 - Information and communications technology professionals			238.9K	70.5K	191.4K	336.4K
26 - Legal, social and cultural professionals						
**3. Technicians and associate professionals**	2.6K	3.9K	34.3K	57.3K	21.9K	85.1K
31 - Science and engineering associate professionals	0.0B	0.0B	0.0B	0.0B	0.0B	0.0B
32 - Health associate professionals	1.3K	2.9K	59.3K	138.3K	41.5K	300.0K
33 - Business and administration associate professionals	40.1K	25.1K	40.1K	78.5K		409.0K
34 - Legal, social, cultural, related associate professionals			14.1K	23.8K	104.0K	35.5K
35 - Information and communications technicians						
**4. Clerical support workers**	42.7K	10.4K	24.4K	166.2K	187.0K	38.4K
41 - General and keyboard clerks	25.1K	7.7K	8.0K	188.5K		209.1K
42 - Customer services clerks			9.2K	84.6K		113.4K
43 - Numerical and material recording clerks			2.7K	28.0K	536.5K	167.1K
44 - Other clerical support workers						
**5. Service and sales workers**	140.8K	52.2K	870.0K	1041.5K	417.1K	585.1K
51 - Personal service workers	97.5K	27.2K	876.3K	1378.1K		721.1K
52 - Sales workers			5.4K	122.7K		295.9K
53 - Personal care workers			579.8K	651.0K		187.2K
54 - Protective services workers						
**6. Skilled agricultural, forestry and fishery workers**	2.0K	0.0B	0.0B	0.0B	1.2K	0.0B

61 - Market-oriented skilled agricultural workers	0.0K		3.1K	0.0B		4.9K
62 - Market-oriented skilled forestry, fishery, hunting workers					0.0B	
63 - Subsistence farmers, fishers, hunters and gatherers						
**7. Craft and related trades workers**	33.5K	2.6K	136.1K	138.7K	87.5K	68.2K
71 - Building and related trades workers, excluding electricians	12.8K	2.2K	68.0K	53.7K	9.8K	102.3K
72 - Metal, machinery and related trades workers	2.4K	0.2K	24.8K	15.9K	30.2K	14.8K
73 - Handicraft and printing workers	2.4K	2.0K	34.3K	31.0K		39.9K
74 - Electrical and electronic trades workers			174.6K	49.9K		38.2K
75 - Food processing, wood working, garment and other craft and related trades						
**8. Plant and machine operators, and assemblers**	4.3K	0.2K	34.7K	40.9K	44.2K	49.8K
81 - Stationary plant and machine operators	12.9K	0.3K	0.4K	43.2K		18.7K
82 - Assemblers	40.4K	1.7K	51.8K	192.6K	270.9K	114.0K
83 - Drivers and mobile plant operators						
**9. Elementary occupations**	93.5K	21.8K	121.8K	465.0K	547.0K	234.8K
91 - Cleaners and helpers	0.0B	1.2K	251.8K	742.1K	516.1K	15.9K
92 - Agricultural, forestry and fishery labourers	1.7K	5.0K	453.9K	760.3K		305.9K
93 - Labourers in mining, construction, manufacturing, transport			1.0K	5.3K		7.4K
94 - Food preparation assistants			53.5K	132.9K		113.0K
95 - Street and related sales and service workers				35.1K		20.9K
96 - Refuse workers and other elementary workers	0.2K	0.5K	7.9K	93.1K	188.5K	21.8K
